# Supplementary figures and images for: Wolbachia endosymbionts manipulate the self-renewal and differentiation of germline stem cells to reinforce fertility of their fruit fly host
Source: PLoS Biol. 2023 Oct 24;21(10):e3002335. doi: 10.1371/journal.pbio.3002335 (PMC10597519; doi:10.1371/journal.pbio.3002335)

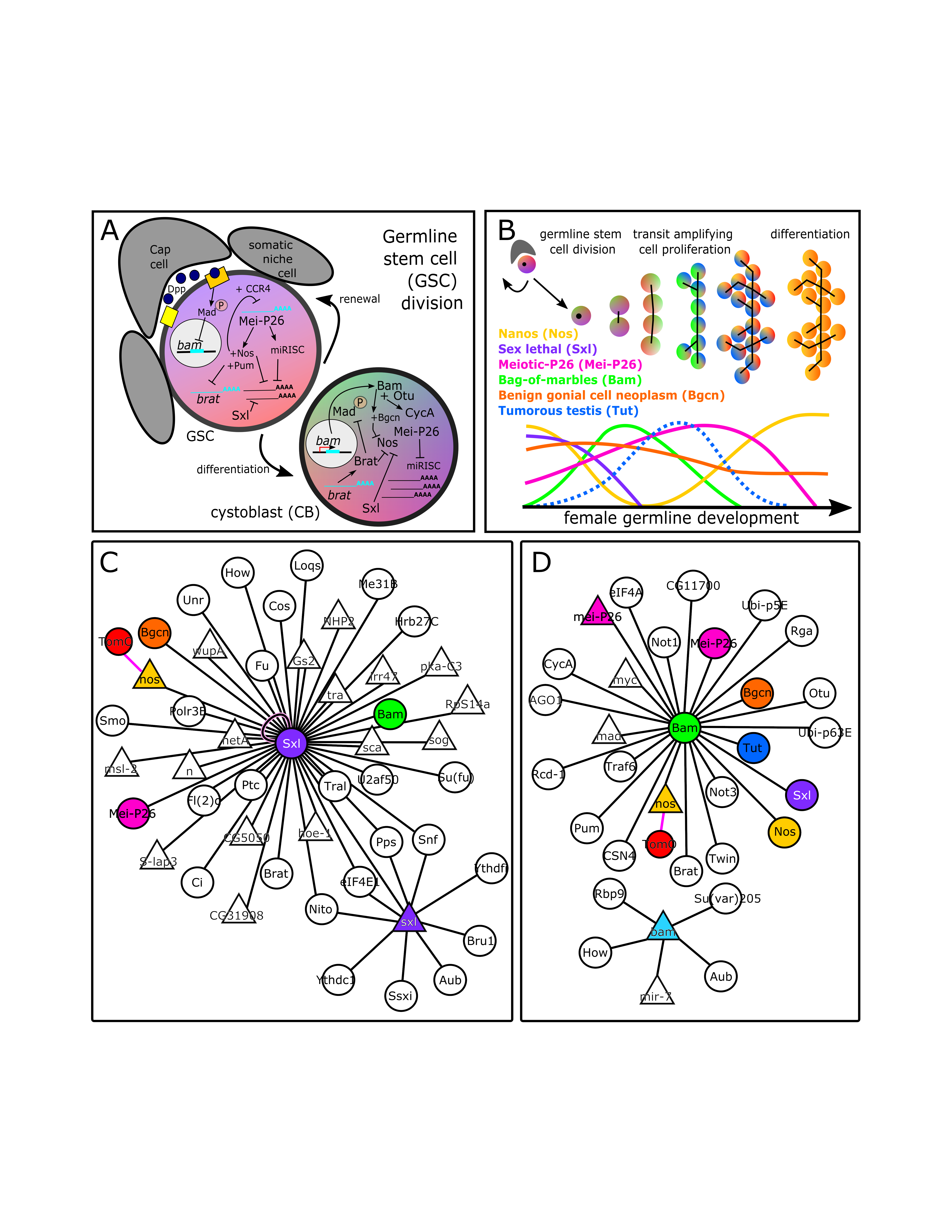

Supplement: S1 Fig — (A) Diagram of the GSC niche illustrating a subset of the genes that shift in expression in the cystoblast following GSC mitosis. (B) Model of the relative levels of protein expression during germline cyst development. (C, D) esyN interaction networks for (C) sxl and (D) bam gene products (supporting references in S1 Table). (TIF) [file pbio.3002335.s001.tif]

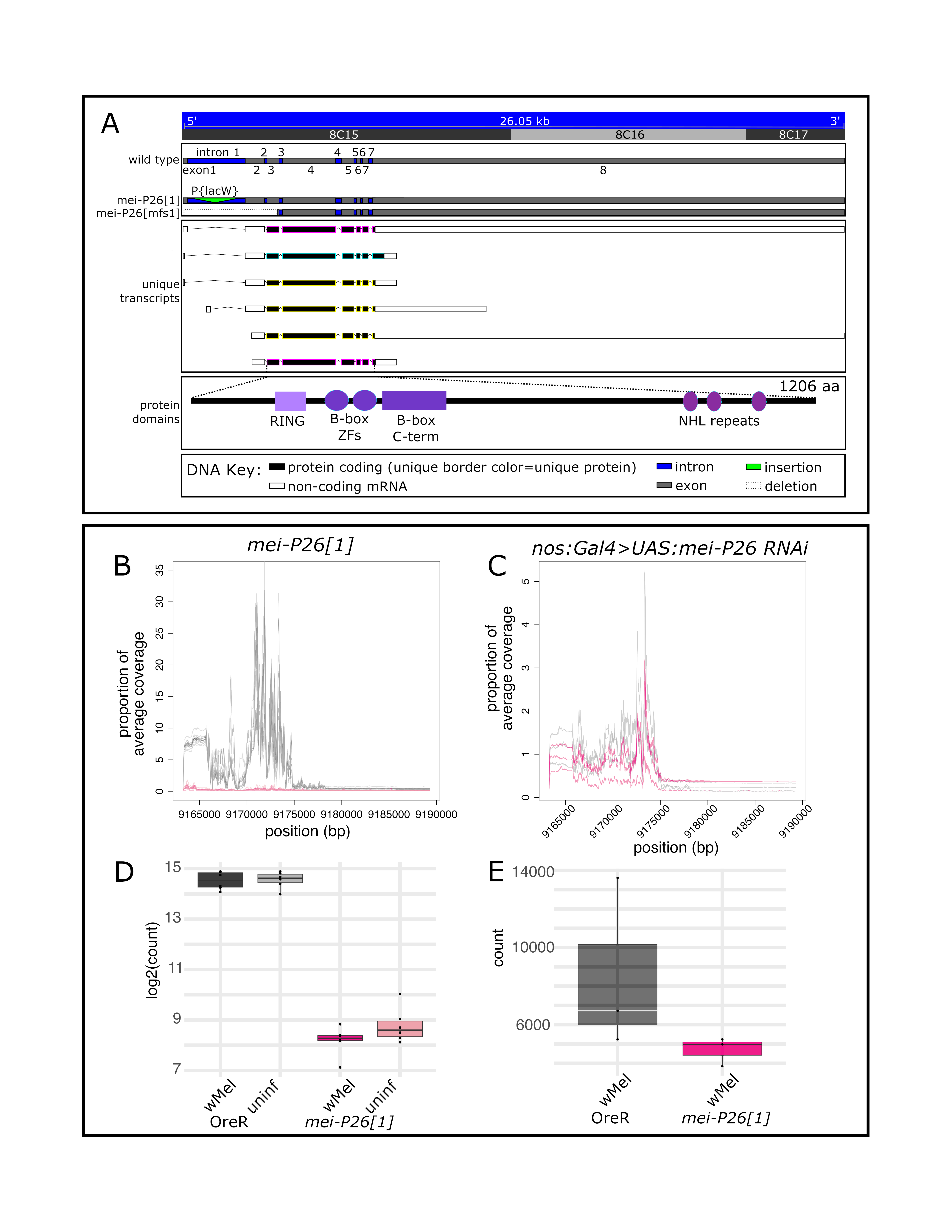

Supplement: S2 Fig — (A) Genomic map and gene model for mei-P26 and the studied alleles. The insertion of a P{lacW} transposon in the first intron of mei-P26[1] impacts the RING domain. The mei-P26[mfs1] allele was generated by deletion of this insertion and 0.7–1.6 kb of DNA flanking each side of the insertion site. (B, C) mei-P26 transcript coverage and (D, E) Kallisto Kallisto normalized transcript counts for D. melanogaster mei-P26 transcripts from (B, D) mei-P26[1] and OreR wMel-infected vs. uninfected ovaries and (C, E) nos:Gal4>UAS:meiP26RNAi vs. OreR wMel-infected ovaries. The data underlying this figure can be found at NCBI, under BioProject number PRJNA1007602. (TIF) [file pbio.3002335.s002.tif]

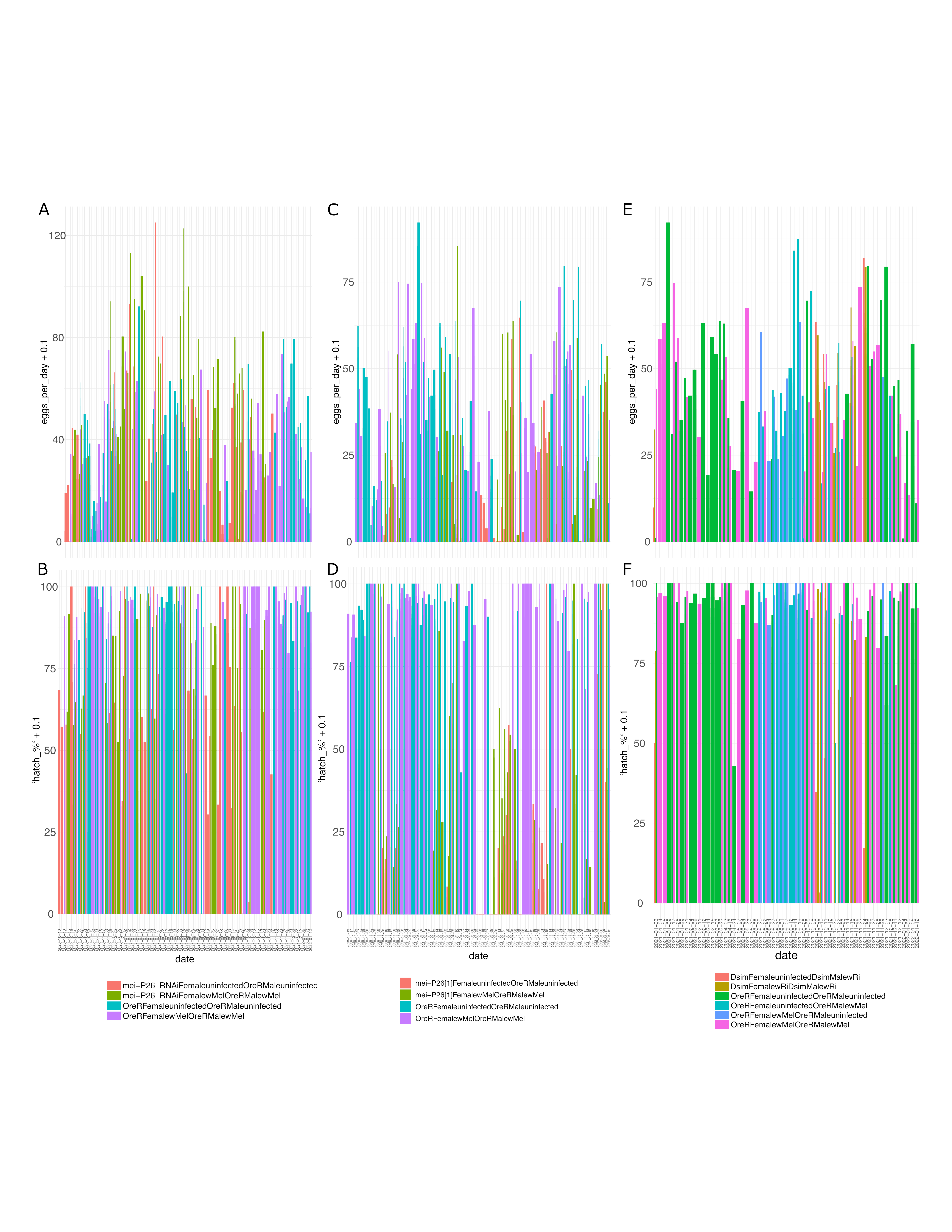

Supplement: S3 Fig — (A, B) Female mei-P26 RNAi, (C, D) Female mei-P26[1], and (E, F) CI assays. Both (A, C, E) egg lay rates and (B, D, F) hatch rates were consistent over time, across genetic crosses, and across fecundity crosses. A factor of 0.1 was added to the y-axis values as an offset to see zero-lay and zero-percent hatch data points. (TIF) [file pbio.3002335.s003.tif]

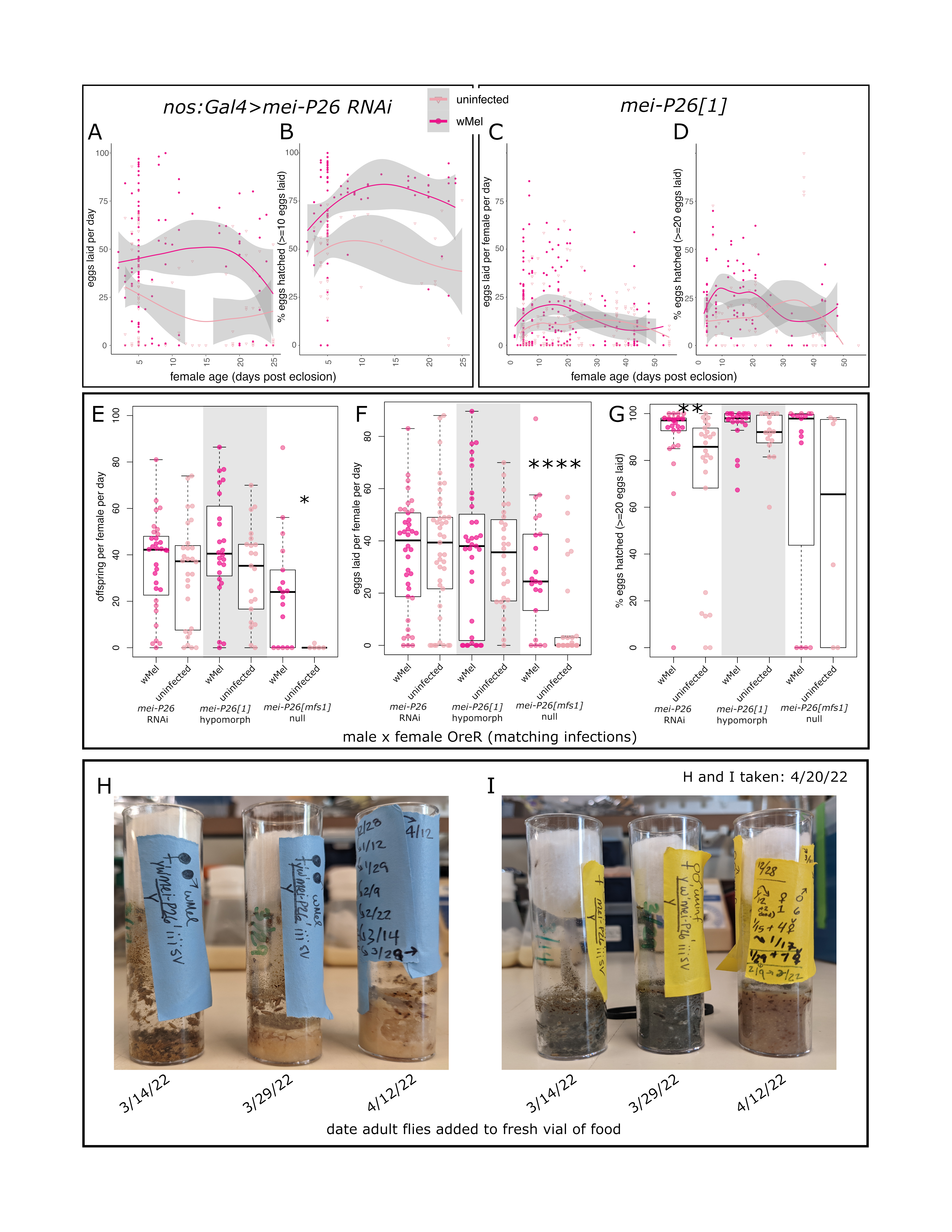

Supplement: S4 Fig — (A, B) Hypomorphic mei-P26[1] and (C, D) nos:Gal4>mei-P26RNAi D. melanogaster female fecundity vs. age, fit with a local polynomial regression (dark gray bounds = 95% confidence intervals). Infection with wMel elevates offspring production across the female lifespan through increasing the number of eggs laid and the proportion of those eggs that hatch. (E–G) Male mei-P26 rescue: wMel infection produced significantly higher rates of (D) overall offspring production, broken into (F) egg lay and (F) egg hatch, in RNAi, hypomorphic, and null mei-P26[1] knockdown male flies mated to wild-type females of the same age and infection status. Wilcoxon rank sum * = p < 0.05, ** = 0.01, **** = 1e-4. The data underlying this figure can be found on Dryad at doi.org/10.7291/D1DT2C. (H, I) Homozygous hypomorphic mei-P26[1] stocks (H) infected with wMel Wolbachia or (I) uninfected. Mold growth (green food vs. tan/brown food) is uninhibited in the uninfected stocks due to embryo and larval death, which both feeds and fails to stop mold. Infection enables stable robust stock persistence because larval production outruns mold growth. Both stocks were started at the same time (see 12/28 on the label). The wMel-infected stock never needed any adults added, whereas the uninfected stock produced too few offspring and had to be supplemented at every vial flip to keep the stock going artificially. We ended this after a few months and the uninfected stock fully died out. (TIFF) [file pbio.3002335.s004.tiff]

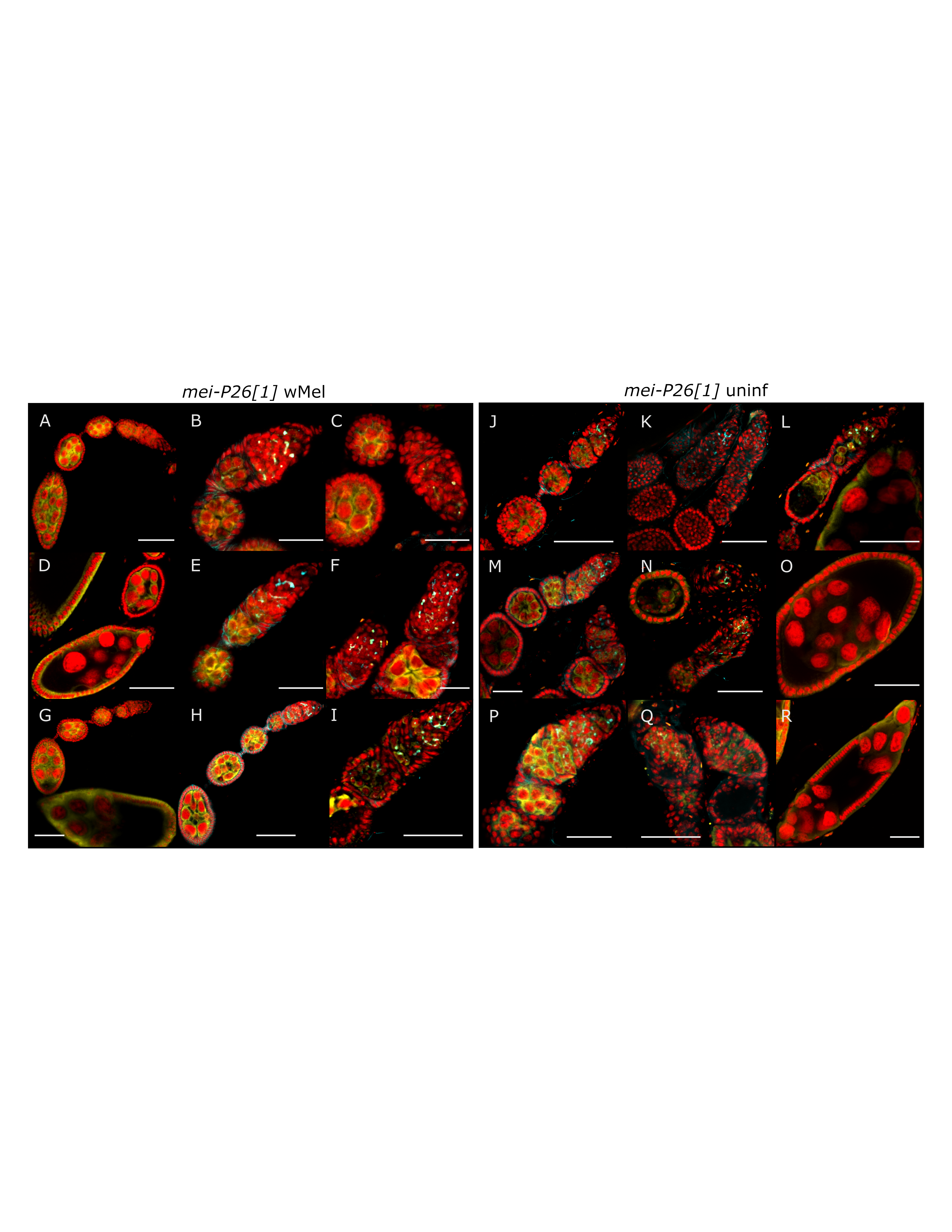

Supplement: S5 Fig — Red = PI DNA staining, yellow = anti-Vas staining, and cyan = anti-Hts staining. Scale bars A, D, G, H, I, J–L, N, O, Q, R = 50 μm; B, C, E, F, M, P = 25 μm. (TIF) [file pbio.3002335.s005.tif]

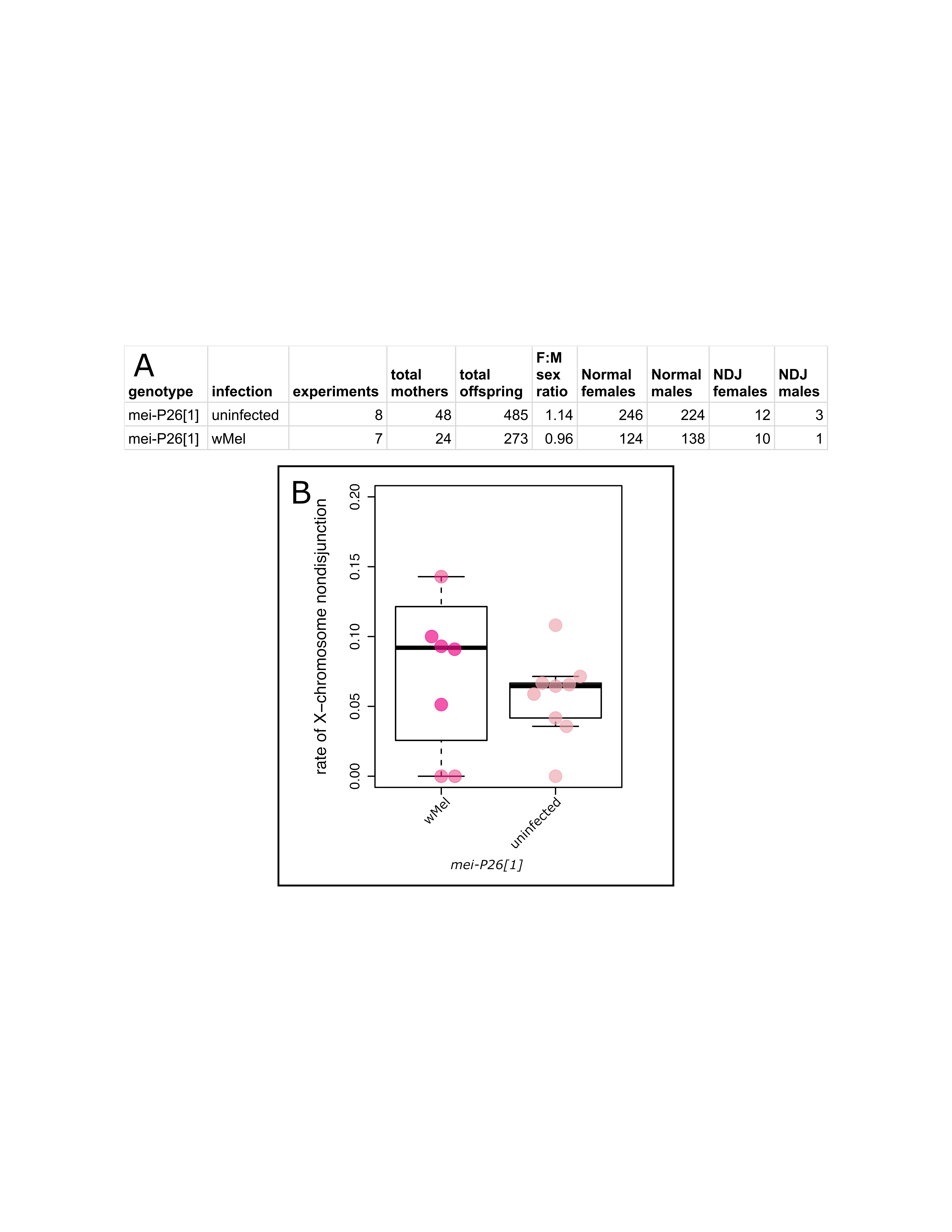

Supplement: S6 Fig — (A) Table containing X-chromosome nondisjunction experimental data. (B) Beeswarm boxplot of the rate of X-chromosome non-disjunction (NDJ) in each experiment. There was no significant difference between infected and uninfected mei-P26[1] females. (TIF) [file pbio.3002335.s006.tif]

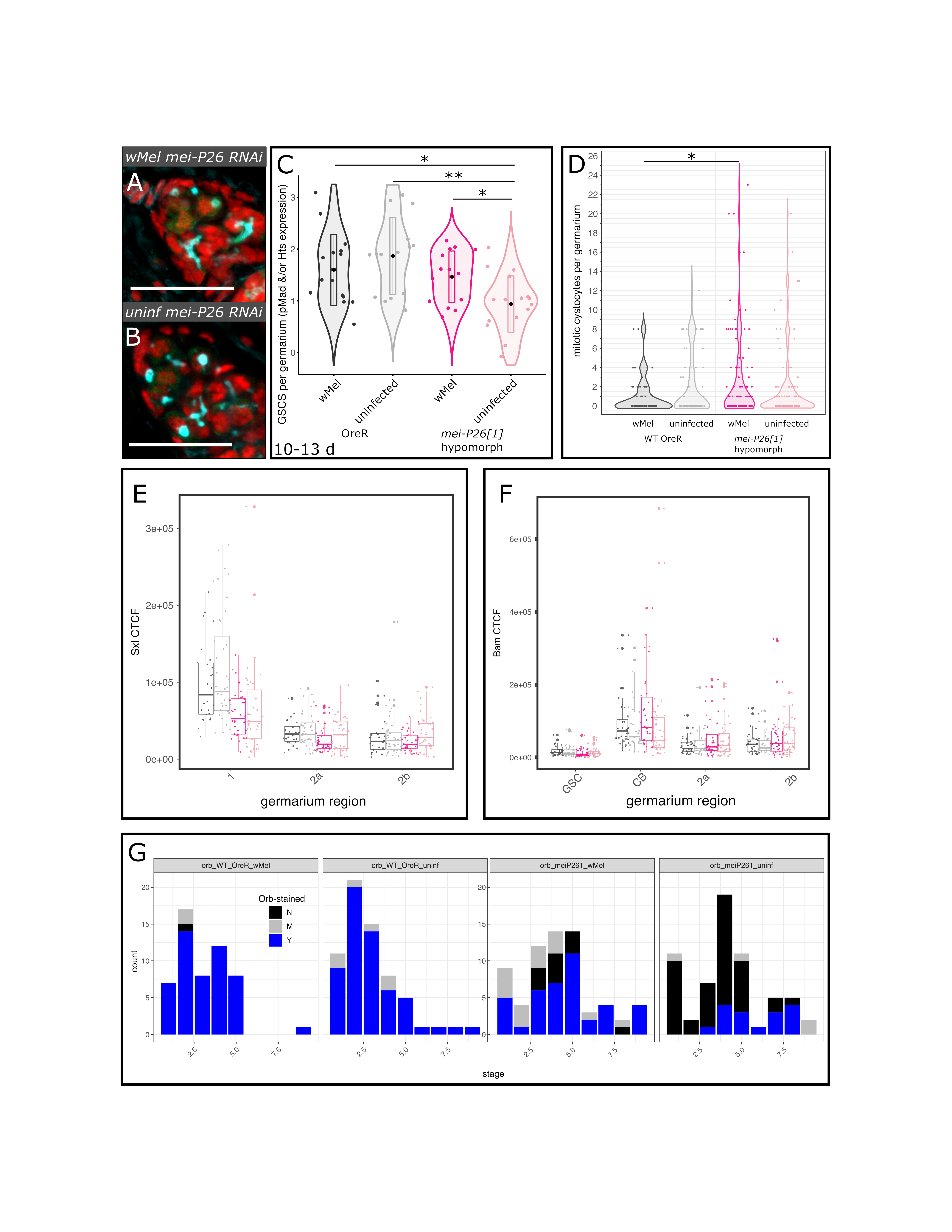

Supplement: S7 Fig — (A, B) Confocal mean projections of D. melanogaster germaria stained with antibodies against Hts and pMad. RNAi knockdown of mei-P26 does not affect GSC maintenance (Fig 2E). (C) Violin plots of the number of GSCs per germarium in 10- to 13-day-old females. As fully functional GSCs express pMad and have Hts-labeled spectrosomes, each was weighted by half and allows for partial scores. Wilcoxon rank sum * = p < 0.05, ** = 0.01. (D) F-K) Violin plots of the number of mitotic cystocytes per germarium detected by pH3 expression. (E, F) Bar-scatter plots of total (E) Sxl and (F) Bam fluorescence expression levels across the germarium, by region. (G) 1D barplots of oocyte-specific Orb staining among germline cysts, distributed across cyst developmental stages. The data underlying this figure can be found on Dryad at doi.org/10.7291/D1DT2C. (TIF) [file pbio.3002335.s007.tif]

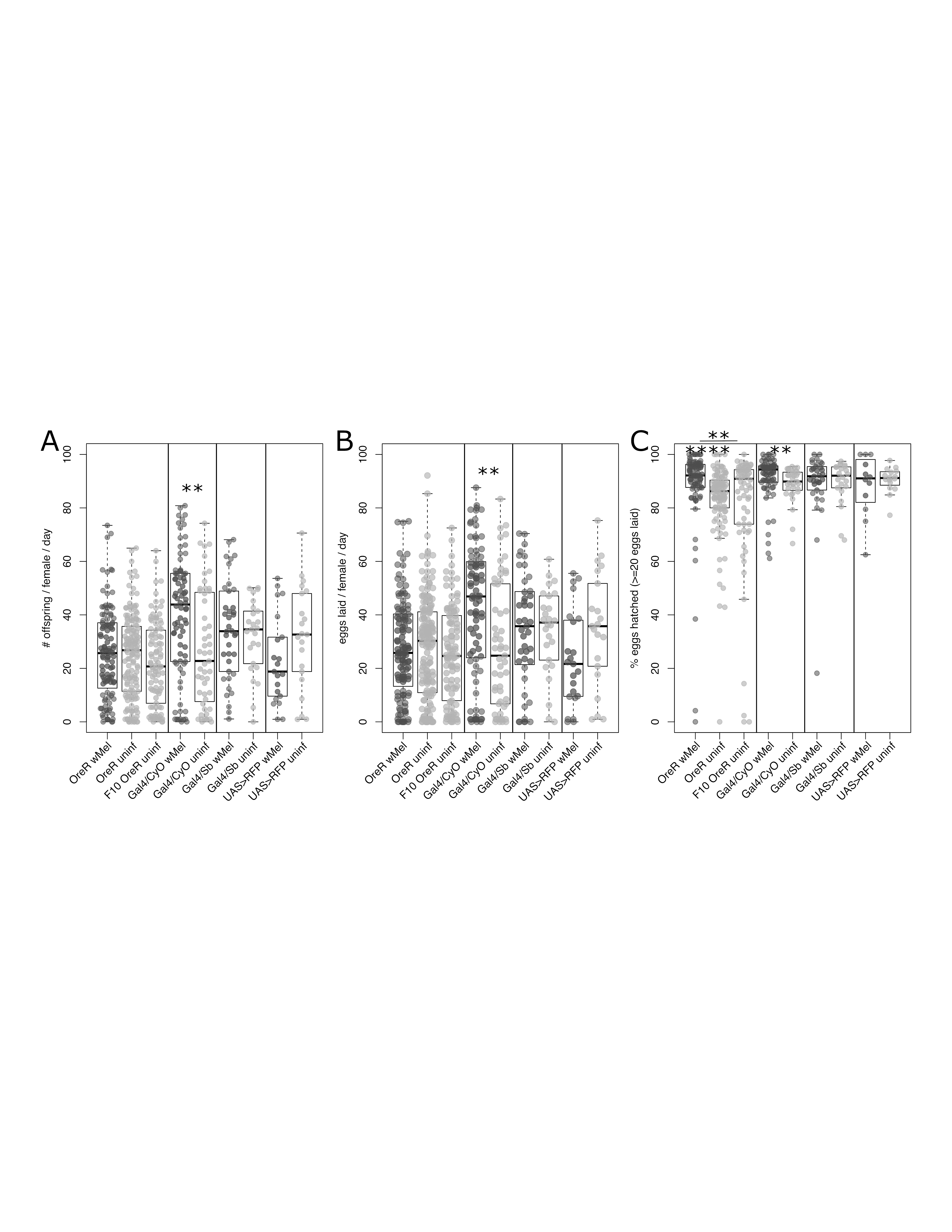

Supplement: S8 Fig — (A–C) Beeswarm boxplots showing that wMel infection elevates wild-type D. melanogaster fertility relative to uninfected flies of the same genotype. (A) Overall offspring production, (B) egg lay, and (C) egg hatch were variably impacted in different “wild-type” genotypes. (D) D. melanogaster eggs laid per female per day plot against female age, fit with a local polynomial regression (dark gray bounds = 95% confidence intervals). The data underlying this figure can be found on Dryad at doi.org/10.7291/D1DT2C. (TIF) [file pbio.3002335.s008.tif]

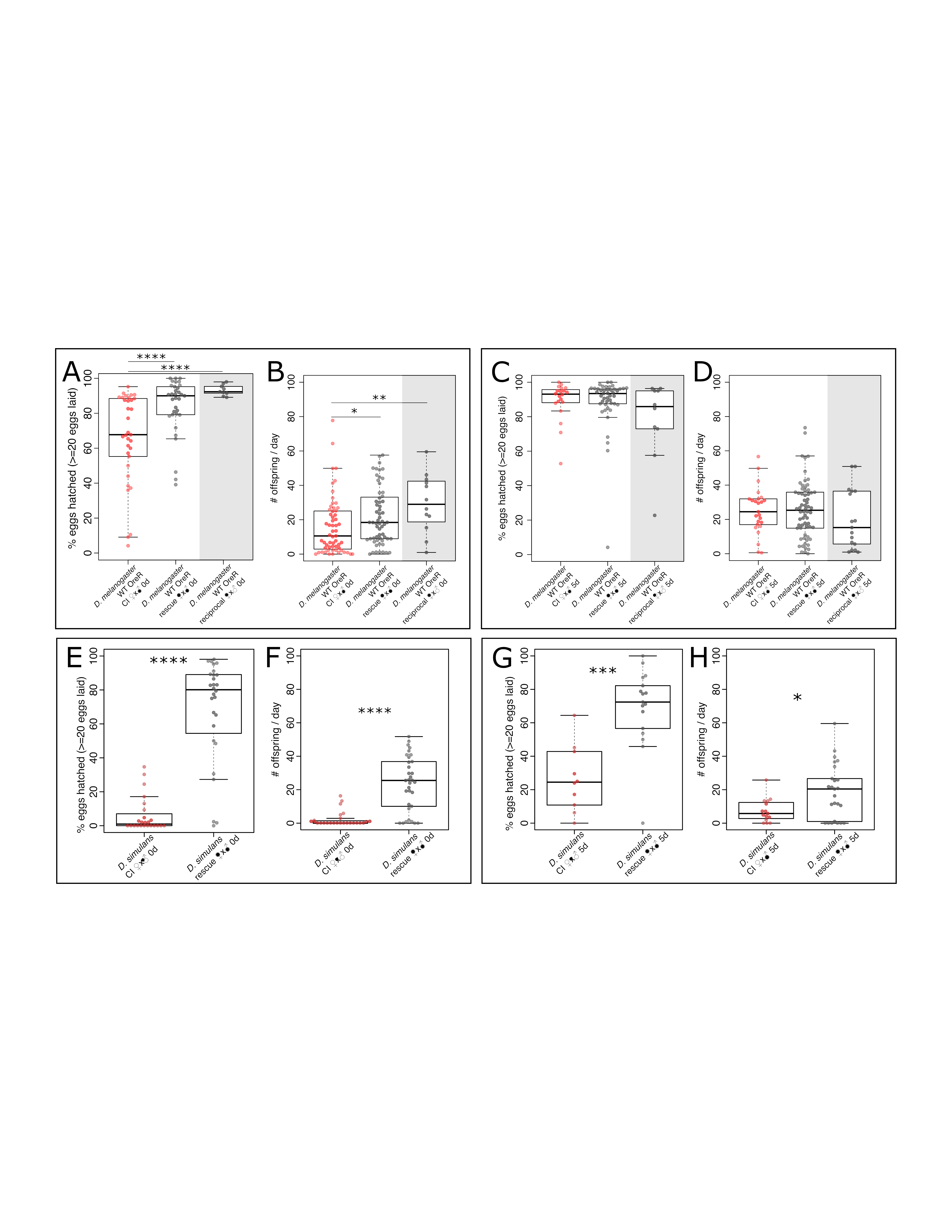

Supplement: S9 Fig — (A–D) Beeswarm box plots of (A, C) egg hatch rate and (B, D) offspring production of uninfected and wMel-infected D. melanogaster OreR females mated to (A, B) zero-day-old and (C, D) 5-day-old wMel-infected males. (E–H) Beeswarm box plots of (E, G) egg hatch rate and (F, G) offspring production in uninfected and wRi-infected D. simulans females mated to (A, B) zero-day-old and (C, D) 5-day-old wRi-infected males. Wilcoxon rank sum * = p < 0.05, ** = 0.01, *** = 0.001, **** = 1e-4. The data underlying this figure can be found on Dryad at doi.org/10.7291/D1DT2C. (TIF) [file pbio.3002335.s009.tif]

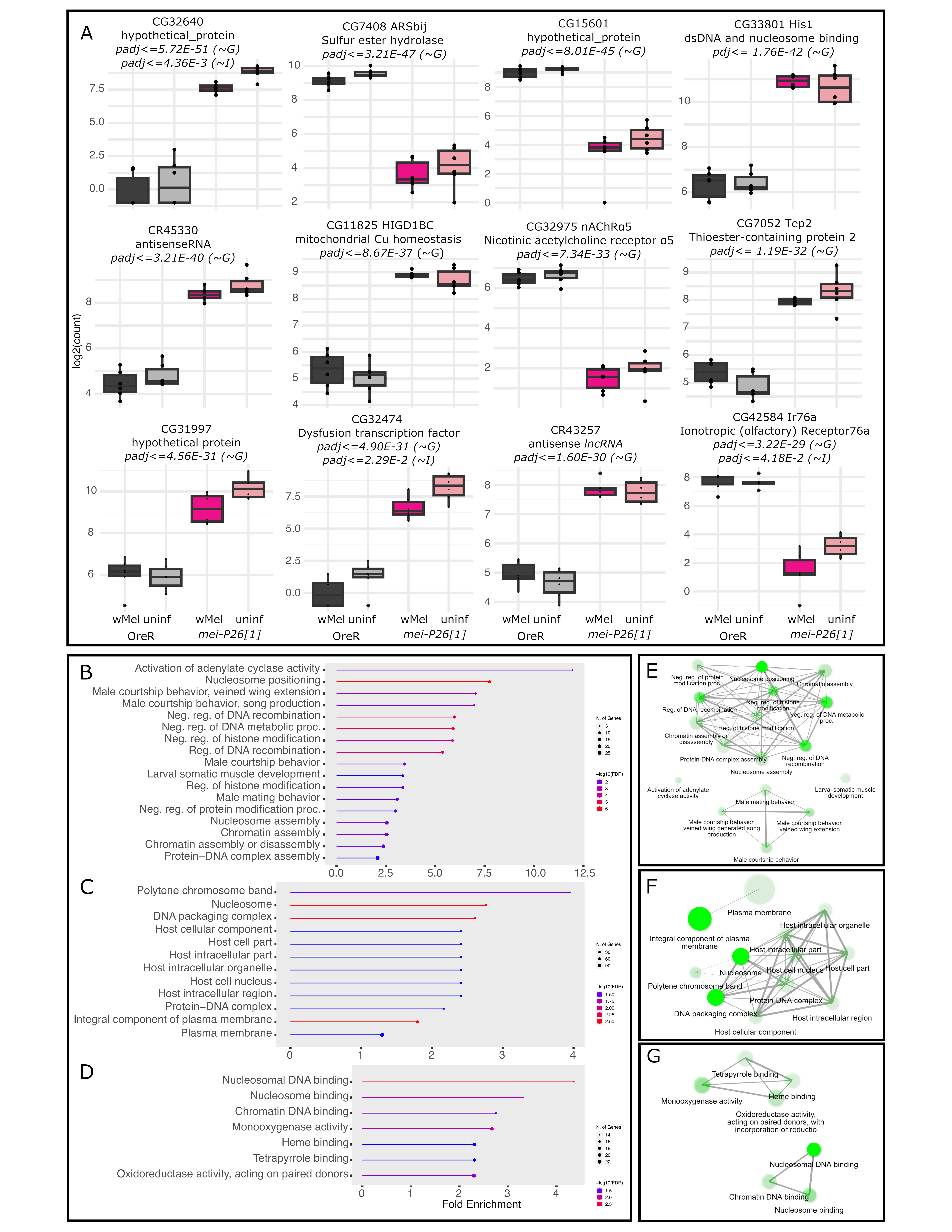

Supplement: S10 Fig — (A) Kallisto normalized transcript counts for D. melanogaster genes (top 15 hits (including Fig 7E) padj< = 2.0E-30; see Figs 7 and S2 for other plots). Barplots are colored by group: dark gray = wMel-infected OreR, light gray = uninfected OreR, dark pink = wMel-infected mei-P26[1], light pink = uninfected mei-P26[1]. (B–G) GO analysis for mei-P26-associated DE genes reveal an enrichment for processes involving chromatin, recombination, protein–protein interactions, and muscle cell differentiation. GO enrichment (B–D) category plots and (E–G) term interaction networks for the categories of (B, E) biological process, (C, F) cellular component, and (D, G) molecular function. The data underlying this figure can be found at NCBI, under BioProject number PRJNA1007602. (TIF) [file pbio.3002335.s010.tif]

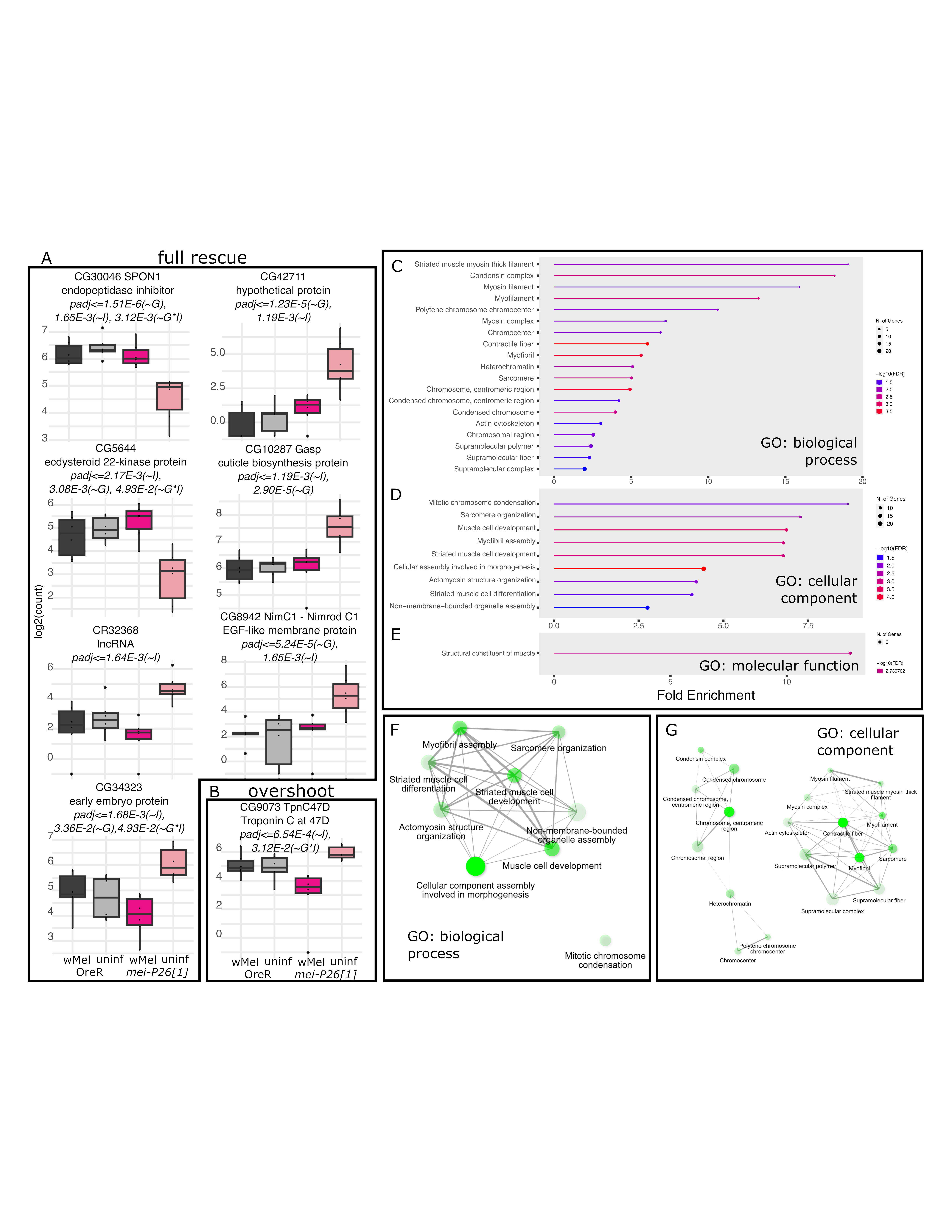

Supplement: S11 Fig — (A, B) Kallisto normalized transcript counts for D. melanogaster genes exhibiting (A) rescue and (B) overshoot of OreR expression levels (padj< = 0.002; see Fig 7 for other plots). Barplots are colored by group: dark gray = wMel-infected OreR, light gray = uninfected OreR, dark pink = wMel-infected mei-P26[1], light pink = uninfected mei-P26[1]. (C–G) GO analysis for infection DE genes reveal an abundance of cytoskeletal and chromatin components. GO enrichment (C–E) category plots and (F, G) term interaction networks for the categories of (C, F) biological process, (D, G) cellular component, and (E) molecular function (no network for a single term). The data underlying this figure can be found at NCBI, under BioProject number PRJNA1007602. (TIF) [file pbio.3002335.s011.tif]

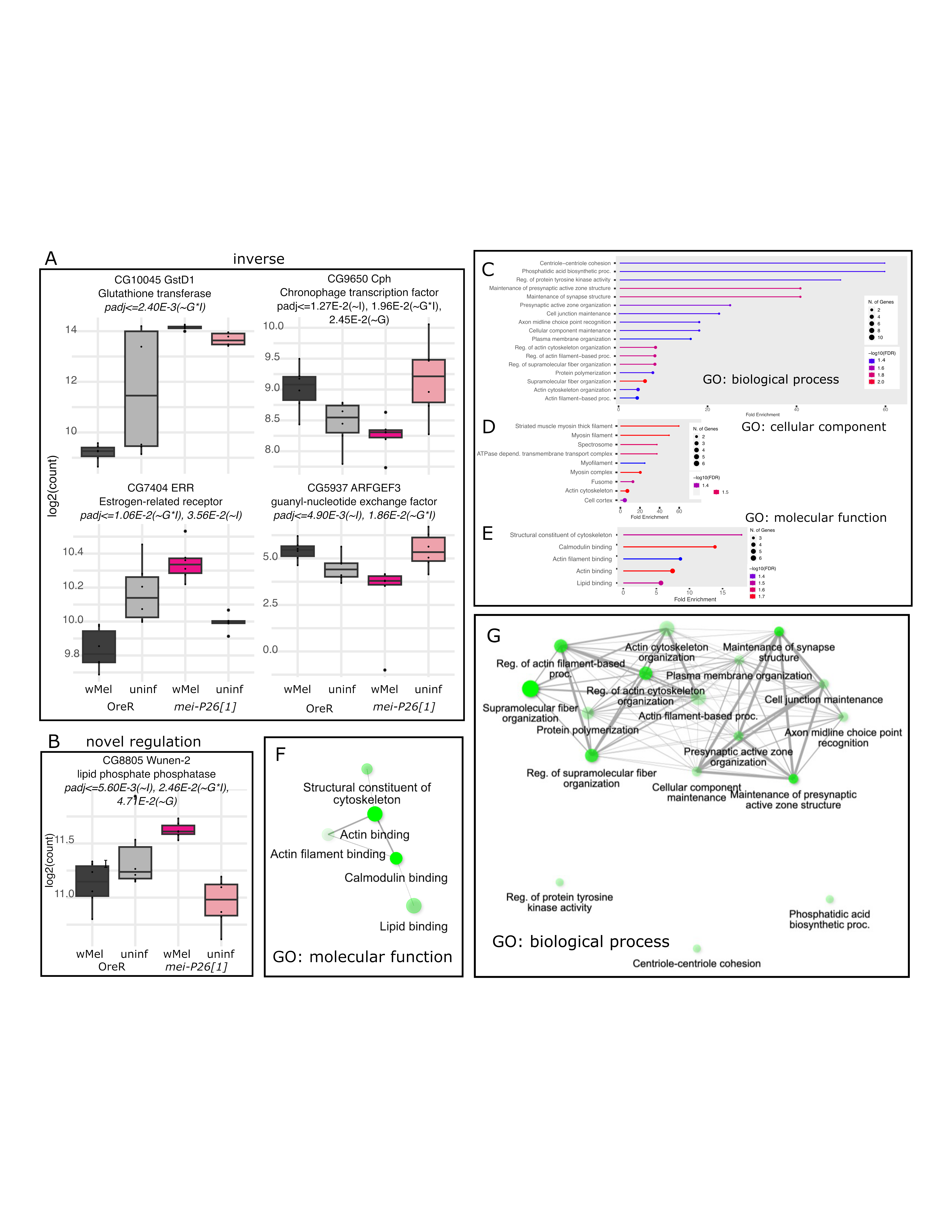

Supplement: S12 Fig — (A–C) Kallisto normalized transcript counts for D. melanogaster genes exhibiting (A) inverse, (B) undershoot, and (C) novel regulation of OreR expression levels (padj< = 0.02; see Fig 7 for other plots). Barplots are colored by group: dark gray = wMel-infected OreR, light gray = uninfected OreR, dark pink = wMel-infected mei-P26[1], light pink = uninfected mei-P26[1]. (D–G) GO analysis for infection DE genes reveal cytoskeletal and membrane factors. GO enrichment (C–E) category plots and (F, G) term interaction networks for the categories of (C, G) biological process, (D) cellular component (see Fig 8D for network), and (E, F) molecular function. The data underlying this figure can be found at NCBI, under BioProject number PRJNA1007602. (TIF) [file pbio.3002335.s012.tif]

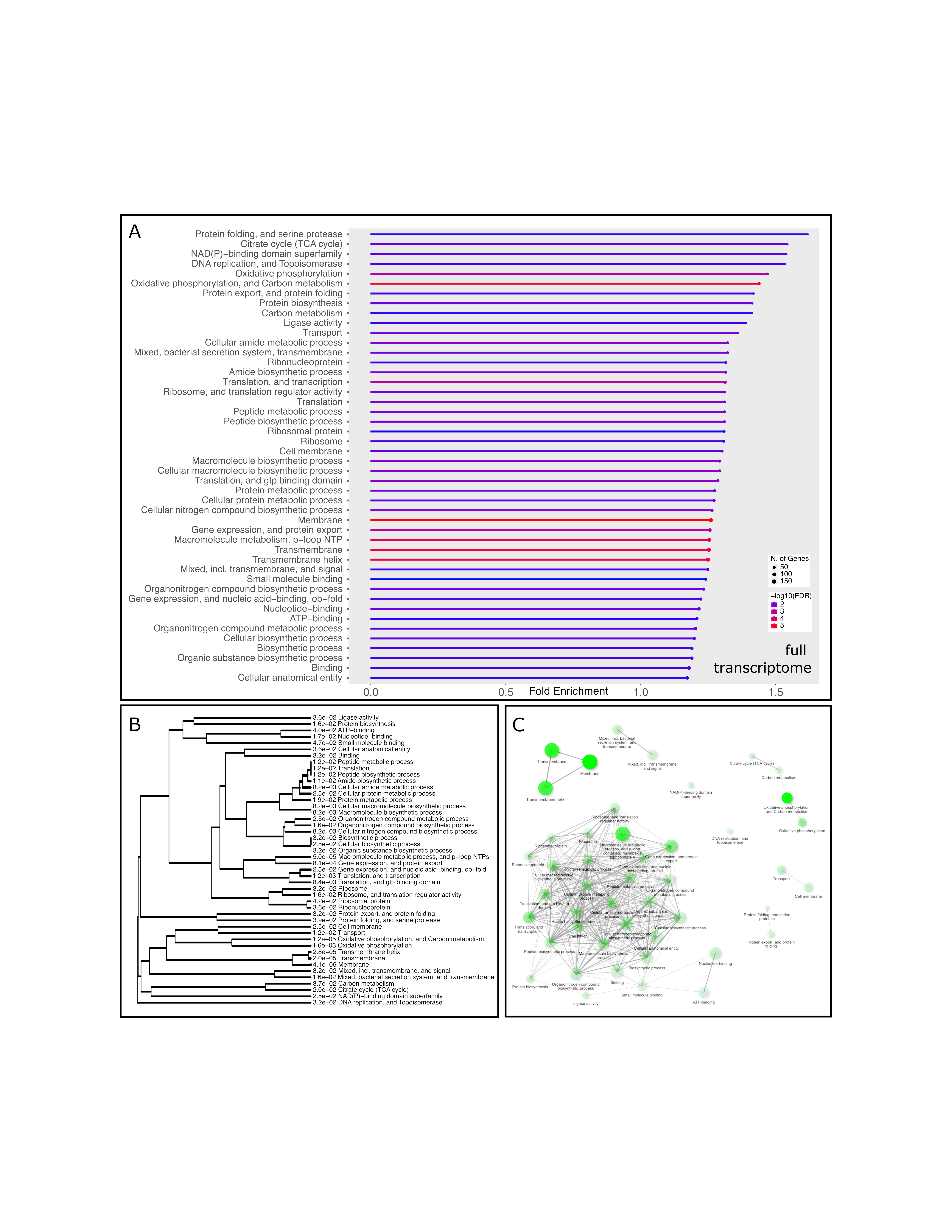

Supplement: S13 Fig — GO enrichment (A) category plot, (B) hierarchical clustering tree, and (C) term interaction network the biological process category. The data underlying this figure can be found at NCBI, under BioProject number PRJNA1007602. (TIFF) [file pbio.3002335.s013.tiff]

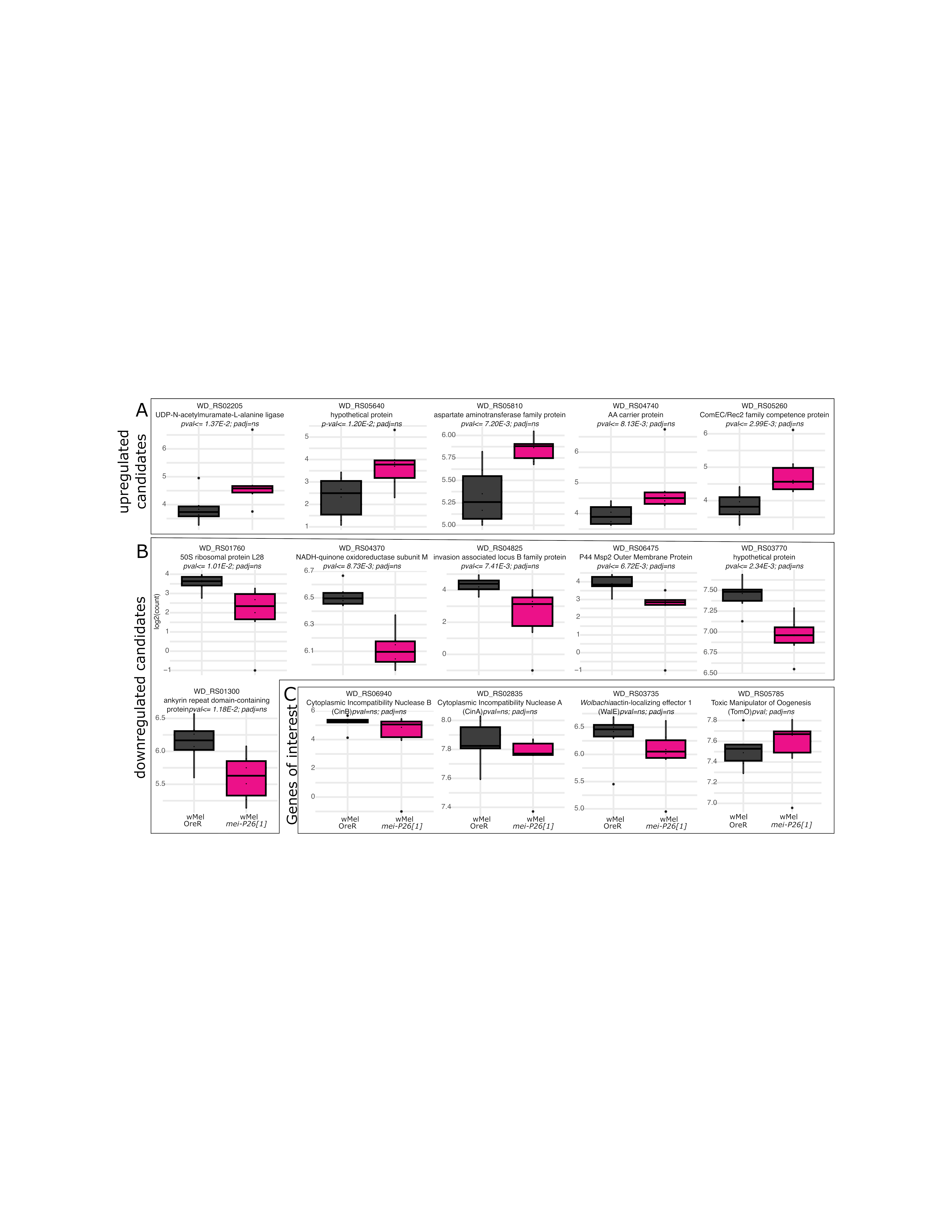

Supplement: S14 Fig — Barplots are colored by group: dark gray = wMel-infected OreR, light gray = uninfected OreR, dark pink = wMel-infected mei-P26[1], light pink = uninfected mei-P26[1]. Wald test genotype-association p-values and adjusted p-values (padj). The data underlying this figure can be found at NCBI, under BioProject number PRJNA1007602. (TIF) [file pbio.3002335.s014.tif]

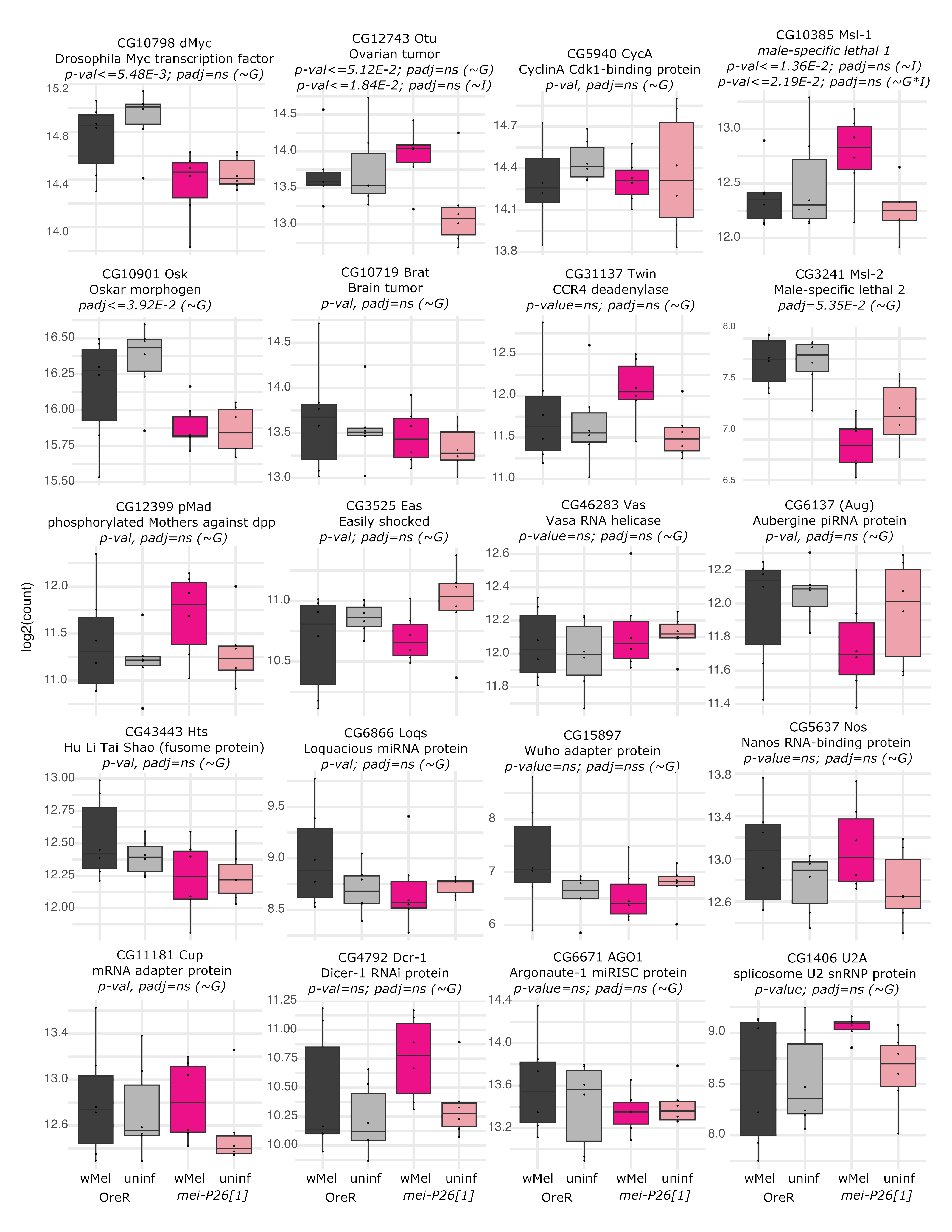

Supplement: S15 Fig — Barplots are colored by group: dark gray = wMel-infected OreR, light gray = uninfected OreR, dark pink = wMel-infected mei-P26[1], light pink = uninfected mei-P26[1]. The data underlying this figure can be found at NCBI, under BioProject number PRJNA1007602. (TIF) [file pbio.3002335.s015.tif]
