# Supplementary material for: Wolbachia endosymbionts manipulate the self-renewal and differentiation of germline stem cells to reinforce fertility of their fruit fly host
Source: PLoS Biol. 2023 Oct 24;21(10):e3002335. doi: 10.1371/journal.pbio.3002335 (PMC10597519; doi:10.1371/journal.pbio.3002335)
Supplement: S1 Table — (PDF) [file pbio.3002335.s016.pdf]

source	type	target	citations
AGO1"	physical	bam	[1]
AGO1"	physical	mei-P26	[2–4]
bam"	physical	aub	[5]
bgcn"	physical	bam	[6–14]
brat"	physical	bam	[1,3]
CG11700"	physical	bam	[15]
CG6304"	physical	mei-P26	[16]
CSN4"	physical	bam	[7]
CycA"	physical	bam	[15]
eIF4A"	physical	bam	[6]
gw"	physical	mei-P26	[4]
how"	physical	bam	[17]
how"	physical	Sxl	[18]
Mad"	physical	bam	[19]
mei-P26"	physical	aub	[5]
mei-P26"	physical	bam	[11]
mei-P26"	physical	bam	[3,10]
mei-P26"	physical	bgcn	[11]
mei-P26"	physical	bgcn	[3,10]
mei-P26"	physical	nos	[10,20]
mei-P26"	physical	nos	[4]
mei-P26"	physical	orb	[4]
mei-P26"	physical	pum	[21,22]
mei-P26"	physical	twin	[21]
mir-137"	physical	mei-P26	[23]
mir-7"	physical	bam	[24]
mir-ban"	physical	mei-P26	[23]

Myc"	physical	bam	[1]
nos"	physical	bam	[20]
nos"	physical	bam	[12]
Not1"	physical	bam	[25]
Not3"	physical	bam	[25]
otu"	physical	bam	[15,26]
pum"	physical	bam	[12]
Rbp9	physical	bam	[27]
Rcd-1"	physical	bam	[25]
Rga"	physical	bam	[25]
Su(var)205"	physical	bam	[28]
Sxl	physical	CG31908	[29]
Sxl"	physical	aub	[5]
Sxl"	physical	bam	[10]
Sxl"	physical	bgcn	[3,10]
Sxl"	physical	brat	[3]
Sxl"	physical	bru1	[30]
Sxl"	physical	CG5050	[29]
Sxl"	physical	ci	[31,32]
Sxl"	physical	cos	[31,32]
Sxl"	physical	eIF4E1	[33]
Sxl"	physical	fl(2)d	[34]
Sxl"	physical	fu	[31]
Sxl"	physical	Gs2	[35]
Sxl"	physical	hoe1	[29]
Sxl"	physical	Hrb27C	[36]
Sxl"	physical	loqs	[18]
Sxl"	physical	Lrr47	[29]
Sxl"	physical	me31B	[18]

Sxl"	physical	mei-P26	[3,10]
Sxl"	physical	msl-2	[18,29,36–43]
Sxl"	physical	N	[44]
Sxl"	physical	NetA	[37]
Sxl"	physical	NHP2	[45]
Sxl"	physical	nito	[46]
Sxl"	physical	nos	[10,20,45,47]
Sxl"	physical	Pka-C3	[37]
Sxl"	physical	Polr3E	[48]
Sxl"	physical	pps	[49]
Sxl"	physical	ptc	[32]
Sxl"	physical	RpS14a	[29]
Sxl"	physical	S-Lap3	[29]
Sxl"	physical	sca	[37]
Sxl"	physical	smo	[32]
Sxl"	physical	snf	[49,50]
Sxl"	physical	snf	[34,49,51]
Sxl"	physical	sog	[37]
Sxl"	physical	ssx	[39]
Sxl"	physical	Su(fu)	[31]
Sxl"	physical	Sxl	[33,37,46,49,52,53]
Sxl"	physical	Sxl	[32]
Sxl"	physical	tra	[35,37,49,54]
Sxl"	physical	tral	[18]
Sxl"	physical	U2af50	[34]
Sxl"	physical	Unr	[18,41,42]
Sxl"	physical	Ythdc1	[52,53]
Sxl"	physical	Ythdf	[52]

Traf6"	physical	bam	[26]
tut	physical	mei-P26	[8]
tut"	physical	bam	[8,14]
twin"	physical	bam	[14,25,55]
U2A"	physical	mei-P26	[56]
Ubi-p5E"	physical	bam	[15]
Ubi-p63E"	physical	bam	[15]
vas"	physical	mei-P26	[57]
wuho"	physical	mei-P26	[58]
wupA"	physical	Sxl	[35]
bam	suppressible	mei-P26	[11]
U2A	suppressible	mei-P26	[56]
mei-P26	suppressible	eas	[59]
mei-P26	suppressible	Dcr-1	[23]
mei-P26	suppressible	twin	[21]
mei-P26	suppressible	jus	[59]
loqs	suppressible	mei-P26	[2]
bam	enhanceable	mei-P26	[60]
vas	enhanceable	mei-P26	[57]

table S1. esyN references for *sxl* and *bam* interactions in Supplemental fig S1C and *mei-P26* interactions in Fig 9.

References:

1. Malik S. Negative regulation of diminutive cancer regulator through differentiation and microRNA pathway components in Drosophila cells. Turk J Biol. 2021;45: 180–186. doi:10.3906/biy-2012-4
2. Neumüller RA, Betschinger J, Fischer A, Bushati N, Poernbacher I, Mechtler K, et al. Mei-P26 regulates microRNAs and cell growth in the Drosophila ovarian stem cell lineage. Nature. 2008;454: 241–245. doi:10.1038/nature07014
3. Malik S, Jang W, Kim C. Protein Interaction Mapping of Translational Regulators Affecting Expression of the Critical Stem Cell Factor Nos. Dev Reprod. 2017;21: 449–456. doi:10.12717/DR.2017.21.4.449
4. Li Y, Maines JZ, Tastan OY, McKearin DM, Buszczak M. Mei-P26 regulates the maintenance of ovarian germline stem cells by promoting BMP signaling. Development. 2012;139: 1547–1556. doi:10.1242/dev.077412
5. Ma X, Zhu X, Han Y, Story B, Do T, Song X, et al. Aubergine Controls Germline Stem Cell Self-Renewal and Progeny Differentiation via Distinct Mechanisms. Dev Cell. 2017;41: 157-169.e5. doi:10.1016/j.devcel.2017.03.023
6. Shen R, Weng C, Yu J, Xie T. eIF4A controls germline stem cell self-renewal by directly inhibiting BAM function in the Drosophila ovary. Proc Natl Acad Sci. 2009;106: 11623–11628.

7. Pan L, Wang S, Lu T, Weng C, Song X, Park JK, et al. Protein competition switches the function of COP9 from self-renewal to differentiation. Nature. 2014;514: 233–236. doi:10.1038/nature13562
8. Chen D, Wu C, Zhao S, Geng Q, Gao Y, Li X, et al. Three RNA Binding Proteins Form a Complex to Promote Differentiation of Germline Stem Cell Lineage in Drosophila. Fuller MT, editor. PLoS Genet. 2014;10: e1004797. doi:10.1371/journal.pgen.1004797
9. Li Y, Minor NT, Park JK, McKearin DM, Maines JZ. Bam and Bgcn antagonize Nanos-dependent germ-line stem cell maintenance. Proc Natl Acad Sci. 2009;106: 9304–9309.
10. Li Y, Zhang Q, Carreira-Rosario A, Maines JZ, McKearin DM, Buszczak M. Mei-P26 Cooperates with Bam, Bgcn and Sxl to Promote Early Germline Development in the Drosophila Ovary. Singh SR, editor. PLoS ONE. 2013;8: e58301. doi:10.1371/journal.pone.0058301
11. Insco ML, Bailey AS, Kim J, Olivares GH, Wapinski OL, Tam CH, et al. A Self-Limiting Switch Based on Translational Control Regulates the Transition from Proliferation to Differentiation in an Adult Stem Cell Lineage. Cell Stem Cell. 2012;11: 689–700. doi:10.1016/j.stem.2012.08.012
12. Kim JY, Lee YC, Kim C. Direct Inhibition of Pumilo Activity by Bam and Bgcn in Drosophila Germ Line Stem Cell Differentiation *. J Biol Chem. 2010;285: 4741–4746. doi:10.1074/jbc.M109.002014
13. Flores HA, Bubnell JE, Aquadro CF, Barbash DA. The *Drosophila* bag of marbles gene interacts genetically with *Wolbachia* and shows female-specific effects of divergence. PLoS Genet. 2015;11: e1005453.
14. Shan L, Wu C, Chen D, Hou L, Li X, Wang L, et al. Regulators of alternative polyadenylation operate at the transition from mitosis to meiosis. J Genet Genomics. 2017;44: 95–106. doi:10.1016/j.jgg.2016.12.007
15. Ji S, Li C, Hu L, Liu K, Mei J, Luo Y, et al. Bam-dependent deubiquitinase complex can disrupt germ-line stem cell maintenance by targeting cyclin A. Proc Natl Acad Sci. 2017;114: 6316–6321. doi:10.1073/pnas.1619188114
16. Guruharsha KG, Rual J-F, Zhai B, Mintseris J, Vaidya P, Vaidya N, et al. A Protein Complex Network of Drosophila melanogaster. Cell. 2011;147: 690–703. doi:10.1016/j.cell.2011.08.047
17. Monk AC, Siddall NA, Volk T, Fraser B, Quinn LM, McLaughlin EA, et al. HOW is required for stem cell maintenance in the Drosophila testis and for the onset of transit-amplifying divisions. Cell Stem Cell. 2010;6: 348–360. doi:10.1016/j.stem.2010.02.016
18. Graindorge A, Carre C, Gebauer F. Sex-lethal promotes nuclear retention of msl2 mRNA via interactions with the STAR protein HOW. Genes Dev. 2013;27: 1421–1433. doi:10.1101/gad.214999.113
19. Malik S, Jang W, Park SY, Kim JY, Kwon K-S, Kim C. The target specificity of the RNA binding protein Pumilio is determined by distinct co-factors. Biosci Rep. 2019;39: BSR20190099. doi:10.1042/BSR20190099
20. Malik S, Jang W, Kim JY, Kim C. Mechanisms ensuring robust repression of the *Drosophila* female germline stem cell maintenance factor Nanos via posttranscriptional regulation. FASEB J. 2020;34: 11421–11430. doi:10.1096/fj.202000656R
21. Joly W, Chartier A, Rojas-Rios P, Busseau I, Simonelig M. The CCR4 Deadenylase Acts with Nanos and Pumilio in the Fine-Tuning of Mei-P26 Expression to Promote Germline Stem Cell Self-Renewal. Stem Cell Rep. 2013;1: 411–424. doi:10.1016/j.stemcr.2013.09.007
22. Flora P, Wong-Deyrup SW, Martin ET, Palumbo RJ, Nasrallah M, Oligney A, et al. Sequential Regulation of Maternal mRNAs through a Conserved cis-Acting Element in Their 3′ UTRs. Cell Rep. 2018;25: 3828-3843.e9. doi:10.1016/j.celrep.2018.12.007
23. Herranz H, Hong X, Pérez L, Ferreira A, Olivieri D, Cohen SM, et al. The miRNA machinery targets Mei-P26 and regulates Myc protein levels in the Drosophila wing. EMBO J. 2010;29: 1688–1698.
24. Pek JW, Lim AK, Kai T. Drosophila Maelstrom Ensures Proper Germline Stem Cell Lineage Differentiation by Repressing microRNA-7. Dev Cell. 2009;17: 417–424. doi:10.1016/j.devcel.2009.07.017
25. Sgromo A, Raisch T, Backhaus C, Keskeny C, Alva V, Weichenrieder O, et al. *Drosophila* Bag-of-marbles directly interacts with the CAF40 subunit of the CCR4–NOT complex to elicit repression of mRNA targets. RNA. 2018;24: 381–395. doi:10.1261/rna.064584.117
26. Ji S, Luo Y, Cai Q, Cao Z, Zhao Y, Mei J, et al. LC Domain-Mediated Coalescence Is Essential for Otu Enzymatic Activity to Extend Drosophila Lifespan. Mol Cell. 2019;74: 363-377.e5. doi:10.1016/j.molcel.2019.02.004
27. Kim-Ha J, Kim J, Kim Y-J. Requirement of RBP9, a *Drosophila* Hu Homolog, for Regulation of Cystocyte Differentiation and Oocyte Determination during Oogenesis. Mol Cell Biol. 1999;19: 2505–2514. doi:10.1128/MCB.19.4.2505
28. Casale AM, Cappucci U, Fanti L, Piacentini L. Heterochromatin protein 1 (HP1) is intrinsically required for post-transcriptional regulation of Drosophila Germline Stem Cell (GSC) maintenance. Sci Rep. 2019;9: 4372. doi:10.1038/s41598-019-40152-1
29. Medenbach J, Seiler M, Hentze MW. Translational Control via Protein-Regulated Upstream Open Reading Frames. Cell. 2011;145: 902–913. doi:10.1016/j.cell.2011.05.005

30. Wang Z, Lin H. Sex-lethal is a target of Bruno-mediated translational repression in promoting the differentiation of stem cell progeny during Drosophila oogenesis. Dev Biol. 2007;302: 160–168. doi:10.1016/j.ydbio.2006.09.016
31. Horabin JI. A positive role for Patched in Hedgehog signaling revealed by the intracellular trafficking of Sex-lethal, the Drosophila sex determination master switch. Development. 2003;130: 6101–6109. doi:10.1242/dev.00865
32. Walthall SL, Moses M, Horabin JI. A large complex containing Patched and Smoothened initiates Hedgehog signaling in Drosophila. J Cell Sci. 2007;120: 826–837. doi:10.1242/jcs.03382
33. Graham PL, Yanowitz JL, Penn JKM, Deshpande G, Schedl P. The Translation Initiation Factor eIF4E Regulates the Sex-Specific Expression of the Master Switch Gene Sxl in Drosophila melanogaster. PLOS Genet. 2011;7: e1002185. doi:10.1371/journal.pgen.1002185
34. Penn JKM, Graham P, Deshpande G, Calhoun G, Chaouki AS, Salz HK, et al. Functioning of the Drosophila Wilms'-Tumor-1-Associated Protein Homolog, Fl(2)d, in Sex-Lethal-Dependent Alternative Splicing. Genetics. 2008;178: 737–748. doi:10.1534/genetics.107.081679
35. Banerjee H, Singh R. Genomic and cDNA selection-amplification identifies transcriptome-wide binding sites for the Drosophila protein sex-lethal. PLOS ONE. 2021;16: e0250592. doi:10.1371/journal.pone.0250592
36. Szostak E, García-Beyaert M, Guitart T, Graindorge A, Coll O, Gebauer F. Hrp48 and eIF3d contribute to msl-2 mRNA translational repression. Nucleic Acids Res. 2018;46: 4099–4113. doi:10.1093/nar/gky246
37. Sandler JE, Irizarry J, Stepanik V, Dunipace L, Amrhein H, Stathopoulos A. A Developmental Program Truncates Long Transcripts to Temporally Regulate Cell Signaling. Dev Cell. 2018;47: 773-784.e6. doi:10.1016/j.devcel.2018.11.019
38. Kelley RL, Wang J, Bell L, Kuroda MI. Sex lethal controls dosage compensation in Drosophila by a non-splicing mechanism. Nature. 1997;387: 195–199. doi:10.1038/387195a0
39. Moschall R, Strauss D, García-Beyaert M, Gebauer F, Medenbach J. *Drosophila* Sister-of-Sex-lethal is a repressor of translation. RNA. 2018;24: 149–158. doi:10.1261/rna.063776.117
40. Hennig J, Militti C, Popowicz GM, Wang I, Sonntag M, Geerlof A, et al. Structural basis for the assembly of the Sxl–Unr translation regulatory complex. Nature. 2014;515: 287–290. doi:10.1038/nature13693
41. Abaza I, Gebauer F. Functional domains of Drosophila UNR in translational control. RNA. 2008;14: 482–490. doi:10.1261/rna.802908
42. Abaza I, Coll O, Patalano S, Gebauer F. Drosophila UNR is required for translational repression of male-specific lethal 2 mRNA during regulation of X-chromosome dosage compensation. Genes Dev. 2006;20: 380–389. doi:10.1101/gad.371906
43. Grskovic M. A co-repressor assembly nucleated by Sex-lethal in the 3'UTR mediates translational control of Drosophila msl-2 mRNA. EMBO J. 2003;22: 5571–5581. doi:10.1093/emboj/cdg539
44. Penn JKM, Schedl P. The master switch gene sex-lethal promotes female development by negatively regulating the N-signaling pathway. Dev Cell. 2007;12: 275–286. doi:10.1016/j.devcel.2007.01.009
45. Morita S, Ota R, Kobayashi S. Downregulation of NHP2 promotes proper cyst formation in *Drosophila* ovary. Dev Growth Differ. 2018;60: 248–259. doi:10.1111/dgd.12539
46. Yan D, Perrimon N. *spenito* is required for sex determination in *Drosophila melanogaster*. Proc Natl Acad Sci. 2015;112: 11606–11611. doi:10.1073/pnas.1515891112
47. Chau J, Kulnane LS, Salz HK. Sex-lethal enables germline stem cell differentiation by down-regulating Nanos protein levels during Drosophila oogenesis. Proc Natl Acad Sci. 2012;109: 9465–9470. doi:10.1073/pnas.1120473109
48. Dong Z, Bell LR. SIN, a novel Drosophila protein that associates with the RNA binding protein Sex-lethal. Gene. 1999;237: 421–428. doi:10.1016/S0378-1119(99)00303-0
49. Johnson ML, Nagengast AA, Salz HK. PPS, a Large Multidomain Protein, Functions with Sex-Lethal to Regulate Alternative Splicing in Drosophila. PLOS Genet. 2010;6: e1000872. doi:10.1371/journal.pgen.1000872
50. Hu J, Cui G, Li C, Liu C, Shang E, Lai L, et al. Structure and Novel Functional Mechanism of Drosophila SNF in Sex-Lethal Splicing. PLOS ONE. 2009;4: e6890. doi:10.1371/journal.pone.0006890
51. Kalifa Y, Armenti ST, Gavis ER. Glorund interactions in the regulation of gurken and oskar mRNAs. Dev Biol. 2009;326: 68–74. doi:10.1016/j.ydbio.2008.10.032
52. Kan L, Grozhik AV, Vedanayagam J, Patil DP, Pang N, Lim K-S, et al. The m6A pathway facilitates sex determination in Drosophila. Nat Commun. 2017;8: 15737. doi:10.1038/ncomms15737
53. Haussmann IU, Bodi Z, Sanchez-Moran E, Mongan NP, Archer N, Fray RG, et al. m6A potentiates Sxl alternative pre-mRNA splicing for robust Drosophila sex

determination. Nature. 2016;540: 301–304. doi:10.1038/nature20577
54. Gillingham AK, Sinka R, Torres IL, Lilley KS, Munro S. Toward a Comprehensive Map of the Effectors of Rab GTPases. Dev Cell. 2014;31: 358–373. doi:10.1016/j.devcel.2014.10.007
55. Fu Z, Geng C, Wang H, Yang Z, Weng C, Li H, et al. Twin Promotes the Maintenance and Differentiation of Germline Stem Cell Lineage through Modulation of Multiple Pathways. Cell Rep. 2015;13: 1366–1379. doi:10.1016/j.celrep.2015.10.017
56. Wu H, Sun L, Wen Y, Liu Y, Yu J, Mao F, et al. Major spliceosome defects cause male infertility and are associated with nonobstructive azoospermia in humans. Proc Natl Acad Sci. 2016;113: 4134–4139. doi:10.1073/pnas.1513682113
57. Liu N, Han H, Lasko P. Vasa promotes Drosophila germline stem cell differentiation by activating mei-P26 translation by directly interacting with a (U)-rich motif in its 3' UTR. Genes Dev. 2009;23: 2742–2752. doi:10.1101/gad.1820709
58. Rastegari E, Kajal K, Tan B-S, Huang F, Chen R-H, Hsieh T-S, et al. WD40 protein Wuho controls germline homeostasis via TRIM-NHL tumor suppressor Mei-p26 in *Drosophila*. Development. 2020;147: dev182063. doi:10.1242/dev.182063
59. Glasscock E. The mei-P26 Gene Encodes a RING Finger B-box Coiled-Coil-NHL Protein That Regulates Seizure Susceptibility in Drosophilia. Genetics. 2005;170: 1677–1689. doi:10.1534/genetics.105.043174
60. Page SL, McKim KS, Deneen B, Van Hook TL, Hawley RS. Genetic Studies of *mei-P26* Reveal a Link Between the Processes That Control Germ Cell Proliferation in Both Sexes and Those That Control Meiotic Exchange in Drosophila. Genetics. 2000;155: 1757.
