# Supplementary material for: Wolbachia endosymbionts manipulate the self-renewal and differentiation of germline stem cells to reinforce fertility of their fruit fly host
Source: PLoS Biol. 2023 Oct 24;21(10):e3002335. doi: 10.1371/journal.pbio.3002335 (PMC10597519; doi:10.1371/journal.pbio.3002335)
Supplement: S2 Table — (PDF) [file pbio.3002335.s017.pdf]

**Interaction (annotated by gene)**	**sex**	**reference**
Bam is a translational repressor in female GSCs	females	[1]
Bam and Bgcn bind the *nos* 3'-UTR and inhibit Nos translation in females	females	[2]
Dpp GSC niche signaling silences *bam* transcription in females	females	[3]
BamF localizes to the fusome/spectrosome continuously and BamC localizes to the cytoplasm in CB-> 8-cell cysts	females	[4]
Bam alters chromatin methylation to activate gene expression in the female germline	females	[5]
Bam binds Otu to deubiquitinate CycA during female TA mitoses	females	[6]
Bam interacts with Bgcn and the CCR4 deadenylase complex to repress GSC maintenance factors in the female germline	females	[7]
In males, Tut binds the *mei-P26* 3'-UTR, which when bound to Bam and Bgcn represses Mei-P26 translation	males	[8]
Bam and Bgcn bind the *mei-P26* 3'-UTR and inhibit Mei-P26 translation in male spermatocytes	males	[9]
Bam is translationally regulated by miRNA binding to its 3'-UTR in the male germline	males	[10]
Bam is translationally regulated by miR-7 in the male germline	males	[11]
Bam transcription is silenced by a histone linker protein	males	[12]
mei-P26 genetically interacts with Bam as a dominant enhancer	females	[13]
mei-P26 binds Ago1 through its NHL domain and inhibits miRNA production; loquacious knockdown suppresses mei-P26 knockdown phenotype	females	[14]
Vas activates *mei-P26* translation by 3'-UTR binding and interaction with eIF5B	females	[15]
mei-P26 down-regulates eIF4E in GSCs	females	[16]
mei-P26 inhibits Orb by Ago1-based miRNA-binding to the Orb 3'-UTR; mei-P26 also down-regulates Brat (and Bam because Brat--|pMad) expression in the GSC	females	[17]
CCR4-NOT works with Nos and Pum to deadenylate mei-P26 in the GSC; CCR4 was present in the GSCs as well as in other cells in the germarium (Figure 1C) where it was mostly cytoplasmic and accumulated in discrete foci, as reported in other cell types in the ovary and embryo (Rouget et al., 2010; Temme et al., 2004; Zaessinger et al., 2006).	females	[18]
"Mei-P26 associates with Bam, Bgcn and Sxl and nanos mRNA during early cyst development, suggesting that this protein helps to repress the translation of nanos mRNA."	females	[19]
"Mei-P26regulates PGC development"	females	[20]
Mei-P26-Bgcn-Bam-Sxl-Brat-Ago1-miR980/miR-1 --| nos 3'-UTR	females	[21]

sisR-1, a stable intronic sequence RNA, negatively regulates mei-P26 in the GSC (through deadenylation)	females	[22]
Wh regulates Mei-p26, and these proteins function together in multiple contexts to control GSC maintenance and differentiation	females	[23]
Mei-P26 structure and RNA binding targets	females	[24]
Aub represses Mei-P26 translation through deadenylation	females	[25]
"mei-P26 mutant cystoblasts fail to down regulate dMyc protein, suggesting a role of Mei-P26 in dMyc repression during the stem cell-cystoblast transition"	females	[26]
Tut binds the long isoform of the mei-P26 3'-UTR; Bam binds Tut on its N-terminus and Bgcn on its C-Terminus to regulate Mei-P26	males	[8]
Mei-P26 facilitates the accumulation of Bam, and then Bam with Bgcn represses the translation of mei-P26	males	[9]
U2A is involved in mei-P26 splicing	males	[27]
Bruno binds to the sxl mRNA 3'-UTR to repress translation	females	[28]
Bam requires Sxl for differentiation	females	[29]
Sxl is required for cell autonomous PGC fate determination	females	[30]
Sxl binds the nos 3'-UTR to down-regulate Nos translation	females	[31]
Sxl review	females	[32]
Sxl binds to 3'-UTRs to control the length distribution of all transcripts	females	[33]
SXL functions with SETDB1 in the assembly of H3K9me3 silencing islands in germ cells	females	[34]
Sxl transcription is repressed by histone lysine methyltransferase (HKMT) Eggless (Egg/dSETDB1), which catalyzes methylation of Histone H3 lysine 9 (H3K9)	females	[35]
Sxl alters poly-A lengths in the female germline	females	[36]
wMel TomO rescues Sxl function in GSC maintenance through derepression of Nos translation	females	[37]
wPip's TomO sequence rescues Sxl in D. melanogaster via nos translational derepression	females	[38]
"TomO associates with orb mRNA, inhibiting interaction with the translation repressor Cup, leading to the precocious translation of Orb"	females	[39]

table S2. Annotated references used to make fig S1A,B and the table in Fig. 9C.

References:

1. Shen R, Weng C, Yu J, Xie T. eIF4A controls germline stem cell self-renewal by directly inhibiting BAM function in the Drosophila ovary. Proc Natl Acad Sci. 2009;106: 11623–11628.
2. Li Y, Minor NT, Park JK, McKearin DM, Maines JZ. Bam and Bgcn antagonize Nanos-dependent germ-line stem cell maintenance. Proc Natl Acad Sci. 2009;106: 9304–9309.
3. Chen D, McKearin D. Dpp Signaling Silences bam Transcription Directly to Establish Asymmetric Divisions of Germline Stem Cells. Curr Biol. 2003;13: 1786–1791. doi:10.1016/j.cub.2003.09.033
4. McKearin D, Ohlstein B. A role for the Drosophila Bag-of-marbles protein in the differentiation of cystoblasts from germline stem cells. Development. 1995;121: 2937–2947.
5. Mukai M, Hira S, Nakamura K, Nakamura S, Kimura H, Sato M, et al. H3K36 Trimethylation-Mediated Epigenetic Regulation is Activated by Bam and Promotes Germ Cell Differentiation During Early Oogenesis in Drosophila. Biol Open. 2015;4: 119–124. doi:10.1242/bio.201410850
6. Ji S, Li C, Hu L, Liu K, Mei J, Luo Y, et al. Bam-dependent deubiquitinase complex can disrupt germ-line stem cell maintenance by targeting cyclin A. Proc Natl Acad Sci. 2017;114: 6316–6321. doi:10.1073/pnas.1619188114
7. Sgromo A, Raisch T, Backhaus C, Keskeny C, Alva V, Weichenrieder O, et al. *Drosophila* Bag-of-marbles directly interacts with the CAF40 subunit of the CCR4–NOT complex to elicit repression of mRNA targets. RNA. 2018;24: 381–395. doi:10.1261/rna.064584.117
8. Chen D, Wu C, Zhao S, Geng Q, Gao Y, Li X, et al. Three RNA Binding Proteins Form a Complex to Promote Differentiation of Germline Stem Cell Lineage in Drosophila. Fuller MT, editor. PLoS Genet. 2014;10: e1004797. doi:10.1371/journal.pgen.1004797
9. Insco ML, Bailey AS, Kim J, Olivares GH, Wapinski OL, Tam CH, et al. A Self-Limiting Switch Based on Translational Control Regulates the Transition from Proliferation to Differentiation in an Adult Stem Cell Lineage. Cell Stem Cell. 2012;11: 689–700. doi:10.1016/j.stem.2012.08.012
10. Eun SH, Stoiber PM, Wright HJ, McMurdie KE, Choi CH, Gan Q, et al. MicroRNAs downregulate Bag of marbles to ensure proper terminal differentiation in the Drosophila male germline. Development. 2013;140: 23–30. doi:10.1242/dev.086397
11. Pek JW, Lim AK, Kai T. Drosophila Maelstrom Ensures Proper Germline Stem Cell Lineage Differentiation by Repressing microRNA-7. Dev Cell. 2009;17: 417–424. doi:10.1016/j.devcel.2009.07.017
12. Carbonell A, Pérez-Montero S, Climent-Cantó P, Reina O, Azorín F. The Germline Linker Histone dBigH1 and the Translational Regulator Bam Form a Repressor Loop Essential for Male Germ Stem Cell Differentiation. Cell Rep. 2017;21: 3178–3189. doi:10.1016/j.celrep.2017.11.060
13. Page SL, McKim KS, Deneen B, Van Hook TL, Hawley RS. Genetic Studies of *mei-P26* Reveal a Link Between the Processes That Control Germ Cell Proliferation in Both Sexes and Those That Control Meiotic Exchange in Drosophila. Genetics. 2000;155: 1757.
14. Neumüller RA, Betschinger J, Fischer A, Bushati N, Poernbacher I, Mechtler K, et al. Mei-P26 regulates microRNAs and cell growth in the Drosophila ovarian stem cell lineage. Nature. 2008;454: 241–245. doi:10.1038/nature07014
15. Liu N, Han H, Lasko P. Vasa promotes Drosophila germline stem cell differentiation by activating mei-P26 translation by directly interacting with a (U)-rich motif in its 3’ UTR. Genes Dev. 2009;23: 2742–2752. doi:10.1101/gad.1820709
16. Song Y, Lu B. Regulation of cell growth by Notch signaling and its differential requirement in normal vs. tumor-forming stem cells in *Drosophila*. Genes Dev. 2011;25: 2644–2658. doi:10.1101/gad.171959.111
17. Li Y, Maines JZ, Tastan OY, McKearin DM, Buszczak M. Mei-P26 regulates the maintenance of ovarian germline stem cells by promoting BMP signaling. Development. 2012;139: 1547–1556. doi:10.1242/dev.077412
18. Joly W, Chartier A, Rojas-Rios P, Busseau I, Simonelig M. The CCR4 Deadenylase Acts with Nanos and Pumilio in the Fine-Tuning of Mei-P26 Expression to Promote Germline Stem Cell Self-Renewal. Stem Cell Rep. 2013;1: 411–424. doi:10.1016/j.stemcr.2013.09.007
19. Li Y, Zhang Q, Carreira-Rosario A, Maines JZ, McKearin DM, Buszczak M. Mei-P26 Cooperates with Bam, Bgcn and Sxl to Promote Early Germline Development in the Drosophila Ovary. Singh SR, editor. PLoS ONE. 2013;8: e58301. doi:10.1371/journal.pone.0058301

20. Jankovics F, Henn L, Bujna Á, Vilmos P, Spirohn K, Boutros M, et al. Functional Analysis of the Drosophila Embryonic Germ Cell Transcriptome by RNA Interference. Jennings B, editor. PLoS ONE. 2014;9: e98579. doi:10.1371/journal.pone.0098579
21. Malik S, Jang W, Kim C. Protein Interaction Mapping of Translational Regulators Affecting Expression of the Critical Stem Cell Factor Nos. Dev Reprod. 2017;21: 449–456. doi:10.12717/DR.2017.21.4.449
22. Wong JT, Akhbar F, Ng AYE, Tay ML-I, Loi GJE, Pek JW. DIP1 modulates stem cell homeostasis in Drosophila through regulation of sisR-1. Nat Commun. 2017;8: 759. doi:10.1038/s41467-017-00684-4
23. Rastegari E, Kajal K, Tan B-S, Huang F, Chen R-H, Hsieh T-S, et al. WD40 protein Wuho controls germline homeostasis via TRIM-NHL tumor suppressor Mei-p26 in *Drosophila*. Development. 2020;147: dev182063. doi:10.1242/dev.182063
24. Salerno-Kochan A, Horn A, Ghosh P, Nithin C, Kościelniak A, Meindl A, et al. Molecular insights into RNA recognition and gene regulation by the TRIM-NHL protein Mei-P26. Life Sci Alliance. 2022;5: e202201418. doi:10.26508/lsa.202201418
25. Rojas‐Ríos P, Chartier A, Pierson S, Simonelig M. Aubergine and piRNAs promote germline stem cell self‐renewal by repressing the proto‐oncogene *Cbl*. EMBO J. 2017;36: 3194–3211. doi:10.15252/embj.201797259
26. Rhiner C, Díaz B, Portela M, Poyatos JF, Fernández-Ruiz I, López-Gay JM, et al. Persistent competition among stem cells and their daughters in the *Drosophila* ovary germline niche. Development. 2009;136: 995–1006. doi:10.1242/dev.033340
27. Wu H, Sun L, Wen Y, Liu Y, Yu J, Mao F, et al. Major spliceosome defects cause male infertility and are associated with nonobstructive azoospermia in humans. Proc Natl Acad Sci. 2016;113: 4134–4139. doi:10.1073/pnas.1513682113
28. Wang Z, Lin H. Sex-lethal is a target of Bruno-mediated translational repression in promoting the differentiation of stem cell progeny during Drosophila oogenesis. Dev Biol. 2007;302: 160–168. doi:10.1016/j.ydbio.2006.09.016
29. Chau J, Kulnane LS, Salz HK. Sex-lethal Facilitates the Transition From Germline Stem Cell to Committed Daughter Cell in the Drosophila Ovary. Genetics. 2009;182: 121–132. doi:10.1534/genetics.109.100693
30. Hashiyama K, Hayashi Y, Kobayashi S. Drosophila Sex lethal Gene Initiates Female Development in Germline Progenitors. Science. 2011;333: 885–888. doi:10.1126/science.1208146
31. Chau J, Kulnane LS, Salz HK. Sex-lethal enables germline stem cell differentiation by down-regulating Nanos protein levels during Drosophila oogenesis. Proc Natl Acad Sci. 2012;109: 9465–9470. doi:10.1073/pnas.1120473109
32. Moschall R, Gaik M, Medenbach J. Promiscuity in post-transcriptional control of gene expression: *Drosophila* sex-lethal and its regulatory partnerships. FEBS Lett. 2017;591: 1471–1488. doi:10.1002/1873-3468.12652
33. Sandler JE, Irizarry J, Stepanik V, Dunipace L, Amrhein H, Stathopoulos A. A Developmental Program Truncates Long Transcripts to Temporally Regulate Cell Signaling. Dev Cell. 2018;47: 773-784.e6. doi:10.1016/j.devcel.2018.11.019
34. Smolko AE, Shapiro-Kulnane L, Salz HK. The H3K9 methyltransferase SETDB1 maintains female identity in Drosophila germ cells. Nat Commun. 2018;9: 4155. doi:10.1038/s41467-018-06697-x
35. Clough E, Tedeschi T, Hazelrigg T. Epigenetic regulation of oogenesis and germ stem cell maintenance by the Drosophila histone methyltransferase Eggless/dSetDB1. Dev Biol. 2014;388: 181–191. doi:10.1016/j.ydbio.2014.01.014
36. Gawande B, Robida MD, Rahn A, Singh R. Drosophila Sex-lethal protein mediates polyadenylation switching in the female germline. EMBO J. 2006;25: 1263–1272. doi:10.1038/sj.emboj.7601022
37. Ote M, Ueyama M, Yamamoto D. *Wolbachia* protein TomO targets nanos mRNA and restores germ stem cells in *Drosophila* sex-lethal mutants. Curr Biol. 2016;26: 2223–2232. doi:10.1016/j.cub.2016.06.054
38. Ote M, Yamamoto D. Enhancing Nanos expression via the bacterial TomO protein is a conserved strategy used by the symbiont *Wolbachia* to fuel germ stem cell maintenance in infected *Drosophila* females. Arch Insect Biochem Physiol. 2018; e21471. doi:10.1002/arch.21471
39. Ote M, Yamamoto D. The *Wolbachia* protein TomO interacts with a host RNA to induce polarization defects in *Drosophila* oocytes. Arch Insect Biochem Physiol. 2018;99: e21475. doi:10.1002/arch.21475
