# Supplementary material for: Wolbachia endosymbionts manipulate the self-renewal and differentiation of germline stem cells to reinforce fertility of their fruit fly host
Source: PLoS Biol. 2023 Oct 24;21(10):e3002335. doi: 10.1371/journal.pbio.3002335 (PMC10597519; doi:10.1371/journal.pbio.3002335)
Supplement: S5 Table — Experimental genotypes, infection statuses, and sexes are listed. The mate for each cross was OreR, of the same infection status, and of the opposite sex as the experimental fly. Males were aged 3–6 days, except for the young male CI crosses, which were aged zero days (distinguished with “-0d” and “-5d” labels). P-values <0.01 are in light green and <0.05 are in dark green for clarity. (PDF) [file pbio.3002335.s020.pdf]

category	group1	group2	n1	mean1	n2	mean2	differential egg laid/day: mean1-mean2	proportion egg laid/day: mean2/mean1	test	**p-value**
wild type (WT) fertility	WT_OreR_wMel	WT_OreR_uninf	115	27.49	164	27.69	-0.20	1.01	Wilcoxon rank sum	9.09E-01
	WT_OreR_uninf	WT_F10_OreR_uninf	""	""	102	25.62	2.07	0.93	Wilcoxon rank sum	4.78E-01
	WT_OreR_wMel	WT_F10_OreR_uninf	""	""	""	""	1.87	0.93	Wilcoxon rank sum	5.18E-01
	nos:Gal4>RFP_wMel	nos:Gal4>RFP_uninf	23	23.42	21	34.87	-11.46	1.49	Wilcoxon rank sum	7.22E-02
	WT_OreR_wMel	nos:Gal4>RFP_wMel	""	""	""	""	-4.08	0.85	Wilcoxon rank sum	3.15E-01
	WT_OreR_uninf	nos:Gal4>RFP_uninf	""	""	""	""	7.18	1.26	Wilcoxon rank sum	1.15E-01
	CyO/nos:Gal4_wMel	CyO/nos:Gal4_uninf	83	42.36	55	28.43	13.93	0.67	Wilcoxon rank sum	2.22E-03
	Sb/nos:Gal4_wMel	Sb/nos:Gal4_uninf	43	34.21	26	34.17	0.03	1.00	Wilcoxon rank sum	1.00E+00
F mei-P26 knockdown	nos:Gal4>meiP26RNAi_F_wMel	nos:Gal4>meiP26RNAi_F_uninf	66	45.89	60	28.89	17.01	0.63	Wilcoxon rank sum	1.17E-03
	mei-P26[1]_F_wMel	mei-P26[1]_F_uninf	73	16.09	45	6.09	10.00	0.38	Wilcoxon rank sum	2.82E-02
	mei-P26[1/mfs1]_F_wMel	mei-P26[1/mfs1]_F_uninf	42	8.40	37	0.18	8.22	0.02	Wilcoxon rank sum	4.71E-05
	mei-P26[mfs1]_F_wMel	mei-P26[mfs1]_F_uninf	25	2.52	19	0.00	2.52	0.00	Wilcoxon rank sum	3.04E-05
WT vs F mei-P26 knockdown	WT_OreR_wMel	nos:Gal4>meiP26RNAi_F_wMel	""	""	""	""	-18.40	1.67	Wilcoxon rank sum	6.99E-05
	WT_OreR_uninf	nos:Gal4>meiP26RNAi_F_uninf	""	""	""	""	-1.20	1.04	Wilcoxon rank sum	5.15E-01
	WT_OreR_wMel	nos:Gal4>meiP26RNAi_F_uninf	""	""	""	""	-1.40	1.05	Wilcoxon rank sum	5.29E-01
	WT_OreR_uninf	nos:Gal4>meiP26RNAi_F_wMel	""	""	""	""	18.20	1.66	Wilcoxon rank sum	3.01E-05
	nos:Gal4>RFP_wMel	nos:Gal4>meiP26RNAi_F_wMel	""	""	""	""	-22.48	1.96	Wilcoxon rank sum	2.17E-03
	nos:Gal4>RFP_uninf	nos:Gal4>meiP26RNAi_F_uninf	""	""	""	""	5.99	0.83	Wilcoxon rank sum	1.68E-01
	nos:Gal4>RFP_wMel	nos:Gal4>meiP26RNAi_F_uninf	""	""	""	""	-5.47	1.23	Wilcoxon rank sum	8.34E-01
	nos:Gal4>RFP_uninf	nos:Gal4>meiP26RNAi_F_wMel	""	""	""	""	-11.02	1.32	Wilcoxon rank sum	1.76E-01
	WT_OreR_uninf	mei-P26[1]_F_wMel	""	""	""	""	-18.20	1.66	Wilcoxon rank sum	3.01E-05
	WT_OreR_wMel	mei-P26[1]_F_wMel	""	""	""	""	11.40	0.59	Wilcoxon rank sum	5.13E-06
	WT_OreR_uninf	mei-P26[1]_F_uninf	""	""	""	""	21.60	0.22	Wilcoxon rank sum	1.03E-11
	WT_OreR_wMel	mei-P26[1/mfs1]_F_wMel	""	""	""	""	19.09	0.31	Wilcoxon rank sum	2.67E-10
	WT_OreR_uninf	mei-P26[1/mfs1]_F_uninf	""	""	""	""	27.51	0.01	Wilcoxon rank sum	< 2.2e-16
	WT_OreR_wMel	mei-P26[mfs1]_F_wMel	""	""	""	""	24.97	0.09	Wilcoxon rank sum	1.12E-10
	WT_OreR_uninf	mei-P26[mfs1]_F_uninf	""	""	""	""	27.69	0.00	Wilcoxon rank sum	6.31E-11
M mei-P26 knockdown	nos:Gal4>meiP26RNAi_M_wMel	nos:Gal4>meiP26RNAi_M_uninf	38	34.84	41	36.30	-1.46	1.04	Wilcoxon rank sum	9.10E-01
	mei-P26[1]_M_wMel	mei-P26[1]_M_uninf	35	34.64	28	33.47	1.18	0.97	Wilcoxon rank sum	9.83E-01

	mei-P26[mfs1]_M_wMel	mei-P26[mfs1]_M_uninf	21	28.79	28	2.51	26.29	0.09	Wilcoxon rank sum	6.10E-06
WT vs M mei-P26 knockdown	WT_OreR_wMel	nos:Gal4>meiP26RNAi_M_wMel	""	""	""	""	-7.35	1.27	Wilcoxon rank sum	3.58E-02
	WT_OreR_uninf	nos:Gal4>meiP26RNAi_M_uninf	""	""	""	""	-8.61	1.31	Wilcoxon rank sum	3.90E-02
	WT_OreR_wMel	mei-P26[1]_M_wMel	""	""	""	""	-7.15	1.26	Wilcoxon rank sum	2.32E-01
	WT_OreR_uninf	mei-P26[1]_M_uninf	""	""	""	""	-5.78	1.21	Wilcoxon rank sum	1.20E-01
	WT_OreR_wMel	mei-P26[mfs1]_M_wMel	""	""	""	""	-1.30	1.05	Wilcoxon rank sum	1.00E+00
	WT_OreR_uninf	mei-P26[mfs1]_M_uninf	""	""	""	""	25.18	0.09	Wilcoxon rank sum	8.59E-12

table S5. Fecundity statistics: eggs produced per female per day in single female-by-single male crosses. Experimental genotypes, infection statuses, and sexes are listed. The mate for each cross was OreR, of the same infection status, and of the opposite sex as the experimental fly. Males were aged 3-6 days, except for the young male CI crosses, which were aged zero days (distinguished with “-0d” and “-5d” labels). P-values <0.01 are in light green and <0.05 are in dark green for clarity.
