# Supplementary material for: Wolbachia endosymbionts manipulate the self-renewal and differentiation of germline stem cells to reinforce fertility of their fruit fly host
Source: PLoS Biol. 2023 Oct 24;21(10):e3002335. doi: 10.1371/journal.pbio.3002335 (PMC10597519; doi:10.1371/journal.pbio.3002335)
Supplement: S6 Table — Experimental genotypes, infection statuses, and sexes are listed. The mate for each cross was OreR, of the same infection status, and of the opposite sex as the experimental fly. Males were aged 3–6 days, except for the young male CI crosses, which were aged zero days (distinguished with “-0d” and “-5d” labels). Sample counts (n1, n2) in parentheses are for Fisher exact tests (samples with hatched eggs vs. no hatched eggs, opposed to % hatch for samples with 20 or more eggs laid). P-values <0.01 are in light green and <0.05 are in dark green for clarity. (PDF) [file pbio.3002335.s021.pdf]

category	group1(lay>=20)	group2(lay>=20)	n1	mean1	n2	mean2	differential hatch: mean1-mean2	proportion hatch/day: mean2/mean1	test	p-value
wild type (WT) fertility	WT_OreR_wMel	WT_OreR_uninf	76	88.40	104	83.18	5.22	0.94	Wilcoxon rank sum	3.46E-07
	WT_OreR_uninf	WT_F10_OreR_uninf	""	""	64	80.88	2.30	0.97	Wilcoxon rank sum	1.17E-01
	WT_OreR_wMel	WT_F10_OreR_uninf	""	""	""	""	7.51	0.92	Wilcoxon rank sum	8.98E-03
	nos:Gal4>RFP_wMel	nos:Gal4>RFP_uninf	12	88.49	16	90.50	-2.01	1.02	Wilcoxon rank sum	9.82E-01
	WT_OreR_wMel	nos:Gal4>RFP_wMel	""	""	""	""	0.09	1.00	Wilcoxon rank sum	7.15E-01
	WT_OreR_uninf	nos:Gal4>RFP_uninf	""	""	""	""	7.32	1.09	Wilcoxon rank sum	5.01E-03
	CyO/nos:Gal4_wMel	CyO/nos:Gal4_uninf	66	91.31	31	88.83	2.47	0.97	Wilcoxon rank sum	2.08E-03
	Sb/nos:Gal4_wMel	Sb/nos:Gal4_uninf	33	88.66	22	89.55	-0.89	1.01	Wilcoxon rank sum	8.37E-01
F mei-P26 knockdown	nos:Gal4>meiP26RNAi_F_wMel	nos:Gal4>meiP26RNAi_F_uninf	53	74.45	33	49.63	24.81	0.67	Wilcoxon rank sum	2.64E-08
	mei-P26[1]_F_wMel	mei-P26[1]_F_uninf	27(73)	24.91	5(45)	18.47	6.43	0.74	Fisher's exact test (with/without_hatch)	2.55E-03
	mei-P26[1/mfs1]_F_wMel	mei-P26[1/mfs1]_F_uninf	7(42)	27.74	5(35)	0.00	27.74	0.00	Wilcoxon rank sum (Fisher’s exact test)	4.20E-03 (1.00E+00)
	mei-P26[mfs1]_F_wMel	mei-P26[mfs1]_F_uninf	5(25)	0.00	5(19)	0.00	0.00	0.00	NA - no females laid >=20 eggs/day	NA
WT vs F mei-P26 knockdown	WT_OreR_wMel	nos:Gal4>meiP26RNAi_F_wMel	""	""	""	""	13.95	0.84	Wilcoxon rank sum	9.11E-07
	WT_OreR_uninf	nos:Gal4>meiP26RNAi_F_uninf	""	""	""	""	33.55	0.60	Wilcoxon rank sum	4.32E-15
	WT_OreR_wMel	nos:Gal4>meiP26RNAi_F_uninf	""	""	""	""	38.76	0.56	Wilcoxon rank sum	3.57E-14
	WT_OreR_uninf	nos:Gal4>meiP26RNAi_F_wMel	""	""	""	""	-8.73	0.90	Wilcoxon rank sum	1.38E-02
	nos:Gal4>RFP_wMel	nos:Gal4>meiP26RNAi_F_wMel	""	""	""	""	14.04	0.84	Wilcoxon rank sum	1.42E-02
	nos:Gal4>RFP_uninf	nos:Gal4>meiP26RNAi_F_uninf	""	""	""	""	40.86	0.55	Wilcoxon rank sum	1.93E-08
	nos:Gal4>RFP_wMel	nos:Gal4>meiP26RNAi_F_uninf	""	""	""	""	38.85	0.56	Wilcoxon rank sum	8.26E-07
	nos:Gal4>RFP_uninf	nos:Gal4>meiP26RNAi_F_wMel	""	""	""	""	16.05	0.82	Wilcoxon rank sum	3.56E-03
	WT_OreR_uninf	mei-P26[1]_F_wMel	""	""	""	""	8.73	0.90	Wilcoxon rank sum	1.38E-02
	WT_OreR_wMel	mei-P26[1]_F_wMel	""	""	""	""	63.49	0.28	Wilcoxon rank sum	4.82E-13
	WT_OreR_uninf	mei-P26[1]_F_uninf	""	""	""	""	64.71	0.22	Wilcoxon rank sum	2.93E-04
	WT_OreR_wMel	mei-P26[1/mfs1]_F_wMel	""	""	""	""	60.66	0.31	Wilcoxon rank sum	5.32E-05
	WT_OreR_uninf	mei-P26[1/mfs1]_F_uninf	""	""	""	""	83.18	0.00	Wilcoxon rank sum	1.97E-04
	WT_OreR_wMel	mei-P26[mfs1]_F_wMel	""	""	""	""	88.40	0.00	Wilcoxon rank sum	2.41E-04
	WT_OreR_uninf	mei-P26[mfs1]_F_uninf	""	""	""	""	83.18	0.00	Wilcoxon rank sum	1.97E-04

M mei-P26 knockdown	nos:Gal4>meiP26RNAi_M_wMel	nos:Gal4>meiP26RNAi_M_uninf	28	90.58	26	72.61	17.97	0.80	Wilcoxon rank sum	3.42E-03
	mei-P26[1]_M_wMel	mei-P26[1]_M_uninf	21	94.86	16	90.64	4.22	0.96	Wilcoxon rank sum	1.03E-01
	mei-P26[mfs1]_M_wMel	mei-P26[mfs1]_M_uninf	15	71.00	2	0.00	71.00	0.00	Wilcoxon rank sum	1.07E-01
WT vs M mei-P26 knockdown	WT_OreR_wMel	nos:Gal4>meiP26RNAi_M_wMel	""	""	""	""	-2.18	1.02	Wilcoxon rank sum	1.65E-02
	WT_OreR_uninf	nos:Gal4>meiP26RNAi_M_uninf	""	""	""	""	10.57	0.87	Wilcoxon rank sum	9.00E-01
	WT_OreR_wMel	mei-P26[1]_M_wMel	""	""	""	""	-6.46	1.07	Wilcoxon rank sum	4.72E-04
	WT_OreR_uninf	mei-P26[1]_M_uninf	""	""	""	""	-7.46	1.09	Wilcoxon rank sum	5.00E-03
	WT_OreR_wMel	mei-P26[mfs1]_M_wMel	""	""	""	""	17.39	0.80	Wilcoxon rank sum	4.01E-01
	WT_OreR_uninf	mei-P26[mfs1]_M_uninf	""	""	""	""	83.18	0.00	Wilcoxon rank sum	1.73E-02
CI crosses	WT_OreR_Dmel_reciprocal-5d	WT_OreR_wMel	10	78.17	""	""	-10.22	1.13	Wilcoxon rank sum	0.612
	WT_OreR_Dmel_reciprocal-5d	WT_OreR_uninf	""	""	""	""	-5.01	1.06	Wilcoxon rank sum	9.91E-04
	WT_OreR_Dmel_CI-0d	WT_OreR_Dmel_rescue-0d	33	66.10	33	84.03	-17.93	1.27	Wilcoxon rank sum	4.63E-04
	WT_OreR_Dmel_CI-0d	WT_OreR_Dmel_reciprocal-0d	""	""	9	93.21	-27.11	1.41	Wilcoxon rank sum	4.56E-05
	WT_OreR_Dmel_rescue-0d	WT_OreR_Dmel_reciprocal-0d	""	""	""	""	-9.18	1.11	Wilcoxon rank sum	1.25E-01
	WT_OreR_Dmel_CI-5d	WT_OreR_Dmel_rescue-5d	20	89.22	45	88.66	0.56	0.99	Wilcoxon rank sum	9.15E-01
	WT_OreR_Dmel_CI-5d	WT_OreR_Dmel_reciprocal-5d	""	""	""	""	11.05	0.88	Wilcoxon rank sum	2.09E-01
	WT_OreR_Dmel_rescue-5d	WT_OreR_Dmel_reciprocal-5d	""	""	""	""	10.49	0.88	Wilcoxon rank sum	9.72E-02
	Dsimulans_CI-0d	Dsimulans_rescue-0d	24	6.07	28	68.43	-62.36	11.27	Wilcoxon rank sum	5.64E-08
	Dsimulans_CI-5d	Dsimulans_rescue-5d	10	26.52	17	69.04	-42.51	2.60	Wilcoxon rank sum	3.63E-04

table S6. Fecundity statistics: percentage of eggs that hatched from single female-by-single male crosses that laid >= 20 eggs. Experimental genotypes, infection statuses, and sexes are listed. The mate for each cross was OreR, of the same infection status, and of the opposite sex as the experimental fly. Males were aged 3-6 days, except for the young male CI crosses, which were aged zero days (distinguished with “-0d” and “-5d” labels). Sample counts (n1,n2) in parentheses are for Fisher Exact Tests (samples with hatched eggs vs no hatched eggs, opposed to % hatch for samples with 20 or more eggs laid). P-values <0.01 are in light green and <0.05 are in dark green for clarity.
