# Supplementary material for: Wolbachia endosymbionts manipulate the self-renewal and differentiation of germline stem cells to reinforce fertility of their fruit fly host
Source: PLoS Biol. 2023 Oct 24;21(10):e3002335. doi: 10.1371/journal.pbio.3002335 (PMC10597519; doi:10.1371/journal.pbio.3002335)
Supplement: S7 Table — (PDF) [file pbio.3002335.s022.pdf]

group1	group2	test	egg lay (#/female/day) p-value	%egg hatch (>10 eggs laid) p-value	offspring (#/female/day) p-value
WT_OreR_wMel	WT_OreR_uninf	Kolmogorov-Smirnov	7.84E-02	7.06E-09	6.02E-02
meiP26RNAi_F_wMel	meiP26RNAi_F_uninf	Kolmogorov-Smirnov	5.92E-06	1.48E-11	3.87E-08
meiP261_F_wMel	meiP261_F_uninf	Kolmogorov-Smirnov	3.41E-02	6.36E-05	1.18E-04

table S7. Fecundity versus age statistics
