# Supplementary material for: Wolbachia endosymbionts manipulate the self-renewal and differentiation of germline stem cells to reinforce fertility of their fruit fly host
Source: PLoS Biol. 2023 Oct 24;21(10):e3002335. doi: 10.1371/journal.pbio.3002335 (PMC10597519; doi:10.1371/journal.pbio.3002335)
Supplement: S8 Table — (PDF) [file pbio.3002335.s023.pdf]

category	group1	group2	n1	mean1	n2	mean2	test	p-value
wild type (WT)	WT_OreR_wMel-5d	WT_OreR_uninf-5d	8	2.313	13	1.923	Wilcoxon rank sum	1.81E-01
	WT_OreR_wMel-10d	WT_OreR_uninf-10d	15	1.600	15	1.867	Wilcoxon rank sum	3.47E-01
	WT_OreR_wMel-31d	WT_OreR_uninf-31d	14	1.321	15	1.533	Wilcoxon rank sum	7.64E-01
	WT_OreR_wMel-5d	WT_OreR_wMel-10d	""	""	""	""	Wilcoxon rank sum	1.93E-02
	WT_OreR_uninf-5d	WT_OreR_uninf-10d	""	""	""	""	Wilcoxon rank sum	8.24E-01
	WT_OreR_wMel-10d	WT_OreR_wMel-31d	""	""	""	""	Wilcoxon rank sum	0.2655
	WT_OreR_uninf-10d	WT_OreR_uninf-31d	""	""	""	""	Wilcoxon rank sum	0.249
F mei-P26 knockdown	meiP26RNAi_F_wMel-5d	meiP26RNAi_F_uninf-5d	31	2.048	23	1.848	Wilcoxon rank sum	6.68E-01
	meiP261_F_wMel-5d	meiP261_F_uninf-5d	42	1.024	73	0.575	Wilcoxon rank sum	2.88E-04
	meiP26RNAi_F_wMel-5d	meiP261_F_wMel-5d	""	""	""	""	Wilcoxon rank sum	4.47E-08
	meiP26RNAi_F_wMel-5d	meiP261_F_uninf-5d	""	""	""	""	Wilcoxon rank sum	4.04E-14
	meiP26RNAi_F_uninf-5d	meiP261_F_uninf-5d	""	""	""	""	Wilcoxon rank sum	3.63E-08
	meiP26RNAi_F_uninf-5d	meiP261_F_wMel-5d	""	""	""	""	Wilcoxon rank sum	3.15E-04
	meiP261_F_wMel-10d	meiP261_F_uninf-10d	14	1.464	16	0.938	Wilcoxon rank sum	1.48E-02
	meiP261_F_wMel-5d	meiP261_F_wMel-10d	""	""	""	""	Wilcoxon rank sum	1.32E-02
	meiP261_F_uninf-5d	meiP261_F_uninf-10d	""	""	""	""	Wilcoxon rank sum	1.37E-02
WT vs F mei-P26 knockdown	WT_OreR_wMel-5d	meiP26RNAi_F_wMel-5d	""	""	""	""	Wilcoxon rank sum	1.94E-01
	WT_OreR_uninf-5d	meiP26RNAi_F_uninf-5d	""	""	""	""	Wilcoxon rank sum	1.00E+00
	WT_OreR_uninf-5d	meiP26RNAi_F_wMel-5d	""	""	""	""	Wilcoxon rank sum	6.12E-01
	WT_OreR_wMel-5d	meiP26RNAi_F_uninf-5d	""	""	""	""	Wilcoxon rank sum	2.33E-01
	WT_OreR_uninf-5d	meiP261_F_uninf-5d	""	""	""	""	Wilcoxon rank sum	3.18E-07
	WT_OreR_wMel-5d	meiP261_F_wMel-5d	""	""	""	""	Wilcoxon rank sum	1.18E-04
	WT_OreR_wMel-5d	meiP261_F_uninf-5d	""	""	""	""	Wilcoxon rank sum	3.36E-06
	WT_OreR_uninf-5d	meiP261_F_wMel-5d	""	""	""	""	Wilcoxon rank sum	2.75E-04
	WT_OreR_wMel-10d	meiP261_F_wMel-10d	""	""	""	""	Wilcoxon rank sum	6.66E-01
	WT_OreR_wMel-10d	meiP261_F_uninf-10d	""	""	""	""	Wilcoxon rank sum	1.10E-02
	WT_OreR_uninf-10d	meiP261_F_wMel-10d	""	""	""	""	Wilcoxon rank sum	1.40E-01
	WT_OreR_uninf-10d	meiP261_F_uninf-10d	""	""	""	""	Wilcoxon rank sum	1.24E-03

table S8. Germline stem cell (GSC) counts per germarium.
