# Supplementary material for: Wolbachia endosymbionts manipulate the self-renewal and differentiation of germline stem cells to reinforce fertility of their fruit fly host
Source: PLoS Biol. 2023 Oct 24;21(10):e3002335. doi: 10.1371/journal.pbio.3002335 (PMC10597519; doi:10.1371/journal.pbio.3002335)
Supplement: S9 Table — (PDF) [file pbio.3002335.s024.pdf]

category	group1	group2	n germaria	n GSCs	n pHH3+ GSCs	proportion GSCs mitotic	n germaria	n GSCs	n pHH3+ GSCs	proportion GSCs mitotic	test	p-value
wild type (WT)	WT_OreR_wMel	WT_OreR_uninf	81	179	10.000	0.056	68	143	9	0.063	Fisher's Exact Test	8.16E-01
mei-P26 knockdown	meiP261_F_wMel	meiP261_F_uninf	111	152	9.000	0.059	111	115	1	0.009	Fisher's Exact Test	4.69E-02
WT vs F mei-P26 knockdown	WT_OreR_uninf	meiP261_F_uninf	""	""		""	""	""		""	Fisher's Exact Test	4.62E-02
	WT_OreR_wMel	meiP261_F_wMel	""	""		""	""	""		""	Fisher's Exact Test	1.00E+00
	WT_OreR_wMel	meiP261_F_uninf	""	""		""	""	""		""	Fisher's Exact Test	5.51E-02
	WT_OreR_uninf	meiP261_F_wMel	""	""		""	""	""		""	Fisher's Exact Test	1.00E+00

table S9. Number of GSCs in mitosis (anti-pHH3-positive staining), per germarium.
