# Supplementary material for: Wolbachia endosymbionts manipulate the self-renewal and differentiation of germline stem cells to reinforce fertility of their fruit fly host
Source: PLoS Biol. 2023 Oct 24;21(10):e3002335. doi: 10.1371/journal.pbio.3002335 (PMC10597519; doi:10.1371/journal.pbio.3002335)
Supplement: S10 Table — (PDF) [file pbio.3002335.s025.pdf]

category	group1	group2	n germaria	avg # mitotic cystocytes / germarium	n germaria	avg # mitotic cystocytes / germarium	test	**p-value**
wild type (WT)	WT_OreR_wMel	WT_OreR_uninf	81	1.079	68	1.957	Wilcoxon Rank Sum Test	3.01E-01
mei-P26 knockdown	meiP261_F_wMel	meiP261_F_uninf	111	2.545	111	1.817	Fisher's Exact Test	1.40E-01
WT vs F mei-P26 knockdown	WT_OreR_uninf	meiP261_F_uninf	""	""	""	""	Fisher's Exact Test	9.52E-01
	WT_OreR_wMel	meiP261_F_wMel	""	""	""	""	Fisher's Exact Test	1.34E-02
	WT_OreR_wMel	meiP261_F_uninf	""	""	""	""	Fisher's Exact Test	2.64E-01
	WT_OreR_uninf	meiP261_F_wMel	""	""	""	""	Fisher's Exact Test	2.26E-01

table S10. Number of cystocytes (CC) in mitosis (anti-pHH3-positive staining), per germarium.
