# Supplementary material for: Wolbachia endosymbionts manipulate the self-renewal and differentiation of germline stem cells to reinforce fertility of their fruit fly host
Source: PLoS Biol. 2023 Oct 24;21(10):e3002335. doi: 10.1371/journal.pbio.3002335 (PMC10597519; doi:10.1371/journal.pbio.3002335)
Supplement: S11 Table — (PDF) [file pbio.3002335.s026.pdf]

category	group1	group2	n1	n2	test	region 1 - relative fluor mean1	region 1 - relative fluor mean2	differential region 1 - |mean1-mean2|	relative fluor region 1 p-value	region 2a - relative fluor mean1	region 2a - relative fluor mean2	differential region 2a - |mean1-mean2|	relative fluor region 2a p-value	region 2b - relative fluor mean1	region 2b - relative fluor mean2	differential region 2b - |mean1-mean2|	relative fluor region 2b p-value
wild type (WT)	WT_OreR_wMel-5d	WT_OreR_uninf-5d	26	40	Wilcoxon rank sum	0.644	0.639	0.005	5.02E-01	0.236	0.197	0.039	1.42E-02	0.172	0.155	0.02	2.64E-01
F mei-P26 knockdown	meiP261_F_wMel-5d	meiP261_F_uninf-5d	36	39	Wilcoxon rank sum	0.527	0.466	0.061	8.40E-03	0.222	0.236	0.014	1.77E-01	0.211	0.262	0.05	1.72E-02
WT vs F mei-P26 knockdown	WT_OreR_uninf-5d	meiP261_F_uninf-5d			Wilcoxon rank sum			0.173	5.67E-10			0.039	8.60E-04			0.11	2.30E-07
	WT_OreR_wMel-5d	meiP261_F_wMel-5d			Wilcoxon rank sum			0.117	2.87E-04			0.014	8.82E-01			0.04	2.21E-02
	WT_OreR_wMel-5d	meiP261_F_uninf-5d			Wilcoxon rank sum			0.178	9.90E-08			0.000	3.08E-01			0.09	5.33E-05
	WT_OreR_uninf-5d	meiP261_F_wMel-5d			Wilcoxon rank sum			0.112	3.78E-06			0.025	9.10E-02			0.06	1.16E-03

table S11. Sxl expression by germarium region, measured by fluorescence intensity.
