# Supplementary material for: Wolbachia endosymbionts manipulate the self-renewal and differentiation of germline stem cells to reinforce fertility of their fruit fly host
Source: PLoS Biol. 2023 Oct 24;21(10):e3002335. doi: 10.1371/journal.pbio.3002335 (PMC10597519; doi:10.1371/journal.pbio.3002335)
Supplement: S12 Table — (PDF) [file pbio.3002335.s027.pdf]

category	group1	group2	n1	n2	test	GSC - relative fluor mean 1	GSC - relative fluor mean2	differential GSC - |mean1-mean2|	relative fluor GSC p-value	CB - relative fluor mean1	CB - relative fluor mean2	differential CB - |mean1-mean2|	relative fluor CB p-value	region 2a - relative fluor mean1	region 2a - relative fluor mean2	differential region 2a - |mean1-mean2|	relative fluor region 2a p-value	region 2b - relative fluor mean1	region 2b - relative fluor mean2	differential region 2b - |mean1-mean2|	relative fluor region 2b p-value
wild type (WT)	WT_OreR_wMel-5d	WT_OreR_uninf-5d	33	29	Wilcoxon rank sum	0.102	0.089	0.012	1.72E-01	0.481	0.469	0.011	6.14E-01	0.174	0.195	0.021	1.72E-01	0.206	0.204	0.002	7.05E-01
F mei-P26 knockdown	meiP261_F_wMel-5d	meiP261_F_uninf-5d	34	39	Wilcoxon rank sum	0.056	0.069	0.012	4.25E-01	0.502	0.397	0.105	1.60E-02	0.179	0.220	0.041	6.21E-02	0.210	0.263	0.053	1.66E-01
WT vs F mei-P26 knockdown	WT_OreR_uninf-5d	meiP261_F_uninf-5d			Wilcoxon rank sum			0.021	1.42E-03			0.073	9.18E-02			0.025	1.74E-01			0.059	3.33E-02
	WT_OreR_wMel-5d	meiP261_F_wMel-5d			Wilcoxon rank sum			0.046	1.28E-06			0.021	4.58E-01			0.005	7.70E-01			0.004	6.14E-01
	WT_OreR_wMel-5d	meiP261_F_uninf-5d			Wilcoxon rank sum			0.033	4.32E-05			0.084	3.40E-02			0.046	1.99E-02			0.057	3.50E-02
	WT_OreR_uninf-5d	meiP261_F_wMel-5d			Wilcoxon rank sum			0.033	5.97E-05			0.033	3.27E-01			0.016	3.27E-01			0.006	5.15E-01

table S12. Bam expression by germarium region, measured by fluorescence intensity.
