# Supplementary material for: Wolbachia endosymbionts manipulate the self-renewal and differentiation of germline stem cells to reinforce fertility of their fruit fly host
Source: PLoS Biol. 2023 Oct 24;21(10):e3002335. doi: 10.1371/journal.pbio.3002335 (PMC10597519; doi:10.1371/journal.pbio.3002335)
Supplement: S13 Table — (PDF) [file pbio.3002335.s028.pdf]

category	group1	group2	n1	n2	test	GSC - relative Bam/pMad fluor mean1	GSC - relative Bam/pMad fluor mean2	differential Bam/pMad GSC - |mean1-mean2|	relative Bam/pMad fluor GSC p-value	CB - relative Bam/pMad fluor mean1	CB - relative Bam/pMad fluor mean2	differential Bam/pMad CB - |mean1-mean2|	relative Bam/pMad fluor CB p-value
wild type (WT)	WT_OreR_wMel-5d	WT_OreR_uninf-5d	21	29	Wilcoxon rank sum	0.467	0.593	0.126	4.43E-02	1.841	1.767	0.074	5.11E-02
F mei-P26 knockdown	meiP261_F_wMel-5d	meiP261_F_uninf-5d	20	27	Wilcoxon rank sum	0.640	1.422	0.783	9.82E-03	2.814	2.142	0.673	1.43E-03
WT vs F mei-P26 knockdown	WT_OreR_uninf-5d	meiP261_F_uninf-5d			Wilcoxon rank sum			0.829	4.04E-04			0.374	2.82E-01
	WT_OreR_wMel-5d	meiP261_F_wMel-5d			Wilcoxon rank sum			0.173	9.08E-01			0.973	2.13E-02
	WT_OreR_wMel-5d	meiP261_F_uninf-5d			Wilcoxon rank sum			0.955	2.51E-04			0.300	3.86E-01
	WT_OreR_uninf-5d	meiP261_F_wMel-5d			Wilcoxon rank sum			0.047	8.57E-02			1.047	1.22E-06

table S13. Relative Bam vs pMad expression, measured by fluorescence, in GSCs.
