# Supplementary material for: Wolbachia endosymbionts manipulate the self-renewal and differentiation of germline stem cells to reinforce fertility of their fruit fly host
Source: PLoS Biol. 2023 Oct 24;21(10):e3002335. doi: 10.1371/journal.pbio.3002335 (PMC10597519; doi:10.1371/journal.pbio.3002335)
Supplement: S14 Table — Normal cysts contain 16 germline-derived cells, 15 nurse cells and 1 oocyte. Cysts containing greater or less than 15 nurse cells were scored as tumorous or abnormal, respectively. (PDF) [file pbio.3002335.s029.pdf]

| | | | | nurse cells in cyst | | | | nurse cells in cyst | | | |
|---|---|---|---|---|---|---|---|---|---|---|---|---|
| category | group1 | group2 | n1 | <15 | 15 | >15 | n2 | <15 | 15 | >15 | test | p-value |
| wild type (WT) | WT_nos:Gal4_wMel | WT_nos:Gal4_uninf | 215 | 0 | 212 | 3 | 216 | 0 | 216 | 0 | Fisher's exact test | 1.23E-01 |
| F mei-P26 knockdown | meiP26RNAi_F_wMel | meiP26RNAi_F_uninf | 86 | 10 | 62 | 14 | 190 | 16 | 107 | 67 | Fisher's exact test | 4.46E-03 |
| | meiP261_F_wMel | meiP261_F_uninf | 444 | 37 | 189 | 218 | 124 | 8 | 47 | 69 | Fisher's exact test | 4.44E-01 |
| | meiP26RNAi_F_wMel | meiP261_F_wMel | "" | "" | "" | "" | "" | "" | "" | "" | Fisher's exact test | 2.24E-08 |
| | meiP26RNAi_F_wMel | meiP261_F_uninf | "" | "" | "" | "" | "" | "" | "" | "" | Fisher's exact test | 1.81E-08 |
| | meiP26RNAi_F_uninf | meiP261_F_uninf | "" | "" | "" | "" | "" | "" | "" | "" | Fisher's exact test | 1.63E-03 |
| | meiP26RNAi_F_uninf | meiP261_F_wMel | "" | "" | "" | "" | "" | "" | "" | "" | Fisher's exact test | 3.87E-03 |
| WT vs F mei-P26 knockdown | WT_nos:Gal4_wMel | meiP26RNAi_F_wMel | "" | "" | "" | "" | "" | "" | "" | "" | Fisher's exact test | 2.72E-12 |
| | WT_nos:Gal4_uninf | meiP26RNAi_F_uninf | "" | "" | "" | "" | "" | "" | "" | "" | Fisher's exact test | < 2.2e-16 |
| | WT_nos:Gal4_uninf | meiP26RNAi_F_wMel | "" | "" | "" | "" | "" | "" | "" | "" | Fisher's exact test | 5.94E-15 |
| | WT_nos:Gal4_wMel | meiP26RNAi_F_uninf | "" | "" | "" | "" | "" | "" | "" | "" | Fisher's exact test | < 2.2e-16 |
| | WT_nos:Gal4_uninf | meiP261_F_uninf | "" | "" | "" | "" | "" | "" | "" | "" | Fisher's exact test | < 2.2e-16 |
| | WT_nos:Gal4_wMel | meiP261_F_wMel | "" | "" | "" | "" | "" | "" | "" | "" | Fisher's exact test | < 2.2e-16 |
| | WT_nos:Gal4_wMel | meiP261_F_uninf | "" | "" | "" | "" | "" | "" | "" | "" | Fisher's exact test | < 2.2e-16 |
| | WT_nos:Gal4_uninf | meiP261_F_wMel | "" | "" | "" | "" | "" | "" | "" | "" | Fisher's exact test | < 2.2e-16 |
| F mei-P26 OE | meiP26OE_F_wMel | meiP26OE_F_uninf | 101 | 7 | 85 | 9 | 138 | 0 | 131 | 7 | Fisher's exact test | 1.63E-03 |
| mei-P26 OE vs mei-P26 knockdown | meiP26OE_F_wMel | meiP26RNAi_F_wMel | "" | "" | "" | "" | "" | "" | "" | "" | Fisher's exact test | 1.36E-01 |
| | meiP26OE_F_wMel | meiP261_F_wMel | "" | "" | "" | "" | "" | "" | "" | "" | Fisher's exact test | 1.31E-15 |
| | meiP26OE_F_wMel | meiP26RNAi_F_uninf | "" | "" | "" | "" | "" | "" | "" | "" | Fisher's exact test | 6.80E-07 |
| | meiP26OE_F_wMel | meiP261_F_uninf | "" | "" | "" | "" | "" | "" | "" | "" | Fisher's exact test | 2.87E-14 |
| | meiP26OE_F_uninf | meiP26RNAi_F_wMel | "" | "" | "" | "" | "" | "" | "" | "" | Fisher's exact test | 7.96E-07 |
| | meiP26OE_F_uninf | meiP261_F_wMel | "" | "" | "" | "" | "" | "" | "" | "" | Fisher's exact test | < 2.2e-16 |
| | meiP26OE_F_uninf | meiP26RNAi_F_uninf | "" | "" | "" | "" | "" | "" | "" | "" | Fisher's exact test | 4.19E-16 |
| | meiP26OE_F_uninf | meiP261_F_uninf | "" | "" | "" | "" | "" | "" | "" | "" | Fisher's exact test | < 2.2e-16 |
| WT vs F mei-P26 OE | meiP26OE_F_wMel | WT_nos:Gal4_wMel | "" | "" | "" | "" | "" | "" | "" | "" | Fisher's exact test | 1.23E-06 |
| | meiP26OE_F_wMel | WT_nos:Gal4_uninf | "" | "" | "" | "" | "" | "" | "" | "" | Fisher's exact test | 4.73E-09 |
| | meiP26OE_F_uninf | WT_nos:Gal4_wMel | "" | "" | "" | "" | "" | "" | "" | "" | Fisher's exact test | 5.24E-02 |
| | meiP26OE_F_uninf | WT_nos:Gal4_uninf | "" | "" | "" | "" | "" | "" | "" | "" | Fisher's exact test | 1.24E-03 |

**table S14.** Tumorous germline cyst counts. Normal cysts contain 16 germline-derived cells, 15 nurse cells and one oocyte. Cysts containing greater or less than 15 nurse cells were scored as tumorous or abnormal, respectively.
