# Supplementary material for: Wolbachia endosymbionts manipulate the self-renewal and differentiation of germline stem cells to reinforce fertility of their fruit fly host
Source: PLoS Biol. 2023 Oct 24;21(10):e3002335. doi: 10.1371/journal.pbio.3002335 (PMC10597519; doi:10.1371/journal.pbio.3002335)
Supplement: S15 Table — (PDF) [file pbio.3002335.s030.pdf]

category	group1	group2	n1	Y	M	N	n2	Y	M	N	test	p-value
wild type (WT)	WT_OreR_wMel-5d	WT_OreR_uninf-5d	53	50	2	1	64	58	6	0	Fisher's Exact Test	2.09E-01
F mei-P26 knockdown	meiP261_F_wMel-5d	meiP261_F_uninf-5d	66	40	15	11	63	16	4	43	Fisher's Exact Test	5.85E-09
WT vs F mei-P26 knockdown	WT_OreR_uninf-5d	meiP261_F_uninf-5d	""	""	""	""	""	""	""	""	Fisher's Exact Test	< 2.2e-16
	WT_OreR_wMel-5d	meiP261_F_wMel-5d	""	""	""	""	""	""	""	""	Fisher's Exact Test	5.61E-05
	WT_OreR_wMel-5d	meiP261_F_uninf-5d	""	""	""	""	""	""	""	""	Fisher's Exact Test	4.51E-16
	WT_OreR_uninf-5d	meiP261_F_wMel-5d	""	""	""	""	""	""	""	""	Fisher's Exact Test	2.97E-05

table S15. Counts of germline cysts exhibiting oocyte-specific Orb expression (Y), unclear staining (M), or no specific expression, indicating developmentally abnormal cysts lacking specified oocytes.
