# Supplementary material for: Wolbachia endosymbionts manipulate the self-renewal and differentiation of germline stem cells to reinforce fertility of their fruit fly host
Source: PLoS Biol. 2023 Oct 24;21(10):e3002335. doi: 10.1371/journal.pbio.3002335 (PMC10597519; doi:10.1371/journal.pbio.3002335)
Supplement: S16 Table — Data deposited under NCBI BioProject number PRJNA1007602. (PDF) [file pbio.3002335.s031.pdf]

protocol	Sample ID	# Read pairs	Yield (Mbases)	Mean Quality Score	% Bases >= 30	Dmel mapped reads	Dmel %	Dmel coverage (calculated)	wMel mapped reads	wMel %	wMel coverage (calculated)
ribodepletion	mei-P261uninfected-1	16566114	4970	35.28	90.31	26794060	80.87%	21.73	142	0.00%	0.02
ribodepletion	mei-P261uninfected-2	24371726	7312	35.23	90.06	39374606	80.78%	31.93	1644	0.00%	0.22
ribodepletion	mei-P261uninfected-3	20726752	6218	35.2	89.96	31465602	75.91%	25.52	130	0.00%	0.02
ribodepletion	mei-P261uninfected-4	22725501	6818	34.99	89.1	33541934	73.80%	27.20	4742	0.01%	0.65
ribodepletion	mei-P261uninfected-5	25921666	7776	35.29	90.4	41658014	80.35%	33.79	92	0.00%	0.01
ribodepletion	mei-P261uninfected-6	21289976	6387	35.38	90.8	32541844	76.43%	26.39	48	0.00%	0.01
ribodepletion	mei-P261wMel-1	30205483	9061	32.9	80.16	36362650	60.19%	29.49	57237	0.09%	7.83
ribodepletion	mei-P261wMel-3	22849556	6855	35.36	90.65	35184986	76.99%	28.54	62646	0.14%	8.57
ribodepletion	mei-P261wMel-4	26232731	7870	35.37	90.71	42053352	80.15%	34.11	118236	0.23%	16.18
ribodepletion	mei-P261wMel-5	23786557	7136	35.41	90.93	39184612	82.37%	31.78	87424	0.18%	11.96
ribodepletion	mei-P261wMel-7	26403318	7921	35.34	90.59	41693804	78.96%	33.82	96438	0.18%	13.20
ribodepletion	mei-P261wMel-8	24804635	7441	35.33	90.55	39188718	78.99%	31.78	80004	0.16%	10.95
ribodepletion	OreRF10uninfected-4	21514878	6454	35.41	90.95	33544436	77.96%	27.21	1670	0.00%	0.23
ribodepletion	OreRF10uninfected-5	21887003	6566	35.4	90.9	33751420	77.10%	27.37	978	0.00%	0.13
ribodepletion	OreRF10uninfected-6	22033197	6610	35.13	89.72	32014844	72.65%	25.97	926	0.00%	0.13
ribodepletion	OreRuninfected-1	22902974	6871	35.42	91	38142430	83.27%	30.94	1000	0.00%	0.14
ribodepletion	OreRuninfected-2	19424258	5827	35.3	90.37	32687632	84.14%	26.51	556	0.00%	0.08
ribodepletion	OreRuninfected-3	25244059	7573	35.25	90.13	39497260	78.23%	32.03	576	0.00%	0.08
ribodepletion	OreRwMelDB-1	23669962	7101	35.36	90.66	40049364	84.60%	32.48	69074	0.15%	9.45
ribodepletion	OreRwMelDB-2	21342747	6403	35.28	90.3	31878812	74.68%	25.86	129552	0.30%	17.73
ribodepletion	OreRwMelDB-3	20997126	6299	35.33	90.52	32964512	78.50%	26.74	272124	0.65%	37.23
ribodepletion	OreRwMelDB-4	26137488	7841	35.31	90.46	44312894	84.77%	35.94	186630	0.36%	25.54
ribodepletion	OreRwMelDB-6	23976948	7193	35.38	90.75	38740764	80.79%	31.42	310028	0.65%	42.42
ribodepletion	OreRwMelDB-7	23816174	7145	35.37	90.75	37387940	78.49%	30.32	228366	0.48%	31.25
poly-A	nos-meiP26RNAi-wMel-1-resub	72190715	21657	35.97	93.99	137328600	95.12%	111.38	3948	0.00%	0.54
poly-A	nos-meiP26RNAi-wMel-2-resub	65207225	19562	35.96	93.99	123616024	94.79%	100.26	2204	0.00%	0.30

poly-A	nos-meiP26RNAi-wMel-3	67418318	20225	35.87	93.54	126461294	93.79%	102.57	6748	0.01%	0.92
poly-A	nosGal4CyO-wMel-1	63388028	19016	35.92	93.81	120645078	95.16%	97.85	7462	0.01%	1.02
poly-A	nosGal4CyO-wMel-2	71148881	21345	35.81	93.18	133691906	93.95%	108.43	5078	0.00%	0.69
poly-A	nosGal4CyO-wMel-3-resub	67506892	20252	35.96	93.95	128140108	94.91%	103.93	3636	0.00%	0.50

table S16. Transcriptomic dataset generated to test the impacts of mei-P26 knockdown and *w*Mel infection. Data deposited under NCBI BioProjectnumber PRJNA992140.
