# Supplementary material for: Wolbachia endosymbionts manipulate the self-renewal and differentiation of germline stem cells to reinforce fertility of their fruit fly host
Source: PLoS Biol. 2023 Oct 24;21(10):e3002335. doi: 10.1371/journal.pbio.3002335 (PMC10597519; doi:10.1371/journal.pbio.3002335)
Supplement: S17 Table — (PDF) [file pbio.3002335.s032.pdf]

gene_id	baseMean	log2FoldChange	lfcSE	stat	pvalue	padj
Dmel_CG32581	498.502	-7.545	0.348	-21.705	1.85E-104	1.97E-100
Dmel_CG12218	12376.792	5.652	0.285	19.841	1.31E-87	7.01E-84
Dmel_CG42565	636.741	-4.964	0.276	-17.965	3.68E-72	1.31E-68
Dmel_CR34601	1403.059	8.261	0.467	17.692	4.87E-70	1.30E-66
Dmel_CG32640	167.387	-7.771	0.501	-15.517	2.67E-54	5.72E-51
Dmel_CG7408	343.022	5.184	0.347	14.940	1.80E-50	3.21E-47
Dmel_CG15601	142.969	4.618	0.317	14.557	5.24E-48	8.01E-45
Dmel_CG33801	977.281	-4.316	0.304	-14.175	1.32E-45	1.76E-42
Dmel_CR45330	212.370	-3.991	0.289	-13.796	2.70E-43	3.21E-40
Dmel_CG11825	247.162	-3.656	0.277	-13.206	8.11E-40	8.67E-37
Dmel_CG33816	11696.267	-9.588	0.748	-12.825	1.18E-37	1.15E-34
Dmel_CG32975	49.803	4.811	0.385	12.492	8.24E-36	7.34E-33
Dmel_CG7052	166.253	-3.532	0.284	-12.447	1.45E-35	1.19E-32
Dmel_CG31997	498.060	-4.245	0.349	-12.147	5.97E-34	4.56E-31
Dmel_CG32474	120.332	-7.181	0.592	-12.135	6.88E-34	4.90E-31
Dmel_CR43257	129.071	-3.145	0.261	-12.032	2.40E-33	1.60E-30
Dmel_CG42584	108.987	4.408	0.374	11.777	5.12E-32	3.22E-29
Dmel_CG8357	611.808	-2.670	0.229	-11.662	2.00E-31	1.19E-28
Dmel_CG32475	179.753	-10.041	0.863	-11.639	2.62E-31	1.47E-28
Dmel_CG2947	1490.588	1.955	0.171	11.448	2.42E-30	1.29E-27
Dmel_CG4125	840.725	3.748	0.333	11.237	2.67E-29	1.36E-26
Dmel_CR45284	114.416	9.866	0.890	11.083	1.51E-28	7.36E-26
Dmel_CR46123	510.503	-3.518	0.323	-10.877	1.48E-27	6.87E-25
Dmel_CG31683	923.718	7.381	0.681	10.838	2.28E-27	1.02E-24
Dmel_CR45631	72.040	-5.945	0.554	-10.723	7.91E-27	3.38E-24
Dmel_CG32600	181.352	3.164	0.296	10.680	1.26E-26	5.17E-24
Dmel_CG10013	137.097	-3.508	0.335	-10.471	1.17E-25	4.65E-23
Dmel_CG14233	178.085	-2.132	0.206	-10.352	4.09E-25	1.56E-22
Dmel_CG6864	56.941	5.134	0.497	10.322	5.61E-25	2.07E-22
Dmel_CG18321	353.613	-2.824	0.274	-10.309	6.39E-25	2.28E-22

Dmel_CG17684	1222.833	2.837	0.277	10.237	1.36E-24	4.69E-22
Dmel_CG13941	124.885	-2.456	0.246	-10.003	1.47E-23	4.92E-21
Dmel_CG8376	116.256	-3.033	0.304	-9.985	1.77E-23	5.75E-21
Dmel_CR45567	40.179	8.757	0.888	9.863	6.00E-23	1.89E-20
Dmel_CG9453	343.636	-2.445	0.248	-9.859	6.28E-23	1.92E-20
Dmel_CG32647	110.757	-3.051	0.313	-9.739	2.05E-22	6.09E-20
Dmel_CG34380	118.947	4.239	0.436	9.712	2.69E-22	7.77E-20
Dmel_CG18188	45.646	-3.579	0.369	-9.703	2.93E-22	8.25E-20
Dmel_CG13138	209.177	4.323	0.449	9.625	6.27E-22	1.72E-19
Dmel_CG18853	75.915	-8.396	0.873	-9.621	6.49E-22	1.74E-19
Dmel_CG33852	7295.263	4.955	0.520	9.520	1.74E-21	4.53E-19
Dmel_CG1842	581.392	2.211	0.238	9.302	1.38E-20	3.51E-18
Dmel_CG42255	42.513	-3.971	0.428	-9.277	1.74E-20	4.24E-18
Dmel_CG42699	188.299	1.987	0.214	9.278	1.72E-20	4.24E-18
Dmel_CG6269	43.176	-3.049	0.330	-9.228	2.76E-20	6.56E-18
Dmel_CG13937	148.226	4.624	0.502	9.215	3.10E-20	7.21E-18
Dmel_CG12112	1047.365	-2.129	0.232	-9.196	3.72E-20	8.46E-18
Dmel_CG17470	57.133	4.145	0.461	8.986	2.57E-19	5.73E-17
Dmel_CG5559	36.773	5.491	0.616	8.919	4.69E-19	1.02E-16
Dmel_CG15312	1267.851	1.384	0.156	8.881	6.65E-19	1.42E-16
Dmel_CG31792	38.167	-4.478	0.506	-8.851	8.70E-19	1.82E-16
Dmel_CG9871	50.560	-6.238	0.716	-8.715	2.90E-18	5.97E-16
Dmel_CG4373	307.928	-4.950	0.573	-8.646	5.35E-18	1.08E-15
Dmel_CG3616	38.041	-3.219	0.374	-8.597	8.15E-18	1.61E-15
Dmel_CG12592	187.503	-3.278	0.382	-8.580	9.47E-18	1.84E-15
Dmel_CG12505	3126.415	-2.939	0.344	-8.542	1.32E-17	2.52E-15
Dmel_CG5744	82.115	-4.651	0.545	-8.534	1.42E-17	2.66E-15
Dmel_CG3578	148.306	-1.836	0.217	-8.471	2.43E-17	4.40E-15
Dmel_CG9170	356.887	-2.386	0.282	-8.472	2.41E-17	4.40E-15
Dmel_CG9168	50.760	3.256	0.386	8.433	3.38E-17	6.03E-15
Dmel_CR34602	25.163	8.166	0.970	8.417	3.85E-17	6.75E-15
Dmel_CG12414	1841.851	1.885	0.225	8.376	5.48E-17	9.46E-15

Dmel_CG32823	73.681	3.720	0.452	8.232	1.85E-16	3.13E-14
Dmel_CR46499	109.382	-2.030	0.248	-8.197	2.46E-16	4.11E-14
Dmel_CG31776	24.796	-8.050	0.984	-8.184	2.74E-16	4.51E-14
Dmel_CG12520	87.565	-2.304	0.285	-8.082	6.36E-16	1.03E-13
Dmel_CG14855	58.734	2.874	0.356	8.081	6.44E-16	1.03E-13
Dmel_CG13579	22.440	-8.336	1.034	-8.059	7.69E-16	1.21E-13
Dmel_CG12275	39.817	-5.503	0.693	-7.944	1.96E-15	3.03E-13
Dmel_CG8821	106.295	3.716	0.473	7.864	3.71E-15	5.67E-13
Dmel_CG16755	68.579	2.852	0.364	7.839	4.54E-15	6.85E-13
Dmel_CR44264	17.002	7.192	0.922	7.803	6.05E-15	8.99E-13
Dmel_CG2893	2410.997	1.218	0.156	7.793	6.53E-15	9.56E-13
Dmel_CG7002	154.856	-3.554	0.457	-7.781	7.19E-15	1.04E-12
Dmel_CG32789	795.569	-1.531	0.197	-7.778	7.35E-15	1.05E-12
Dmel_CG31617	8715.429	2.006	0.258	7.769	7.90E-15	1.10E-12
Dmel_CG33813	8715.429	2.006	0.258	7.769	7.90E-15	1.10E-12
Dmel_CG16957	23.805	5.408	0.697	7.759	8.59E-15	1.18E-12
Dmel_CG7900	157.605	3.411	0.441	7.744	9.66E-15	1.31E-12
Dmel_CG14444	6588.810	-0.958	0.124	-7.713	1.23E-14	1.64E-12
Dmel_CG6658	157.093	-3.339	0.433	-7.707	1.29E-14	1.70E-12
Dmel_CG33868	2586.412	-2.187	0.284	-7.690	1.47E-14	1.92E-12
Dmel_CG15706	424.122	1.599	0.209	7.659	1.88E-14	2.42E-12
Dmel_CR43960	157.786	-2.537	0.334	-7.598	3.00E-14	3.82E-12
Dmel_CR46481	1090.588	-1.945	0.257	-7.572	3.68E-14	4.63E-12
Dmel_CR45323	25.859	-6.703	0.889	-7.541	4.68E-14	5.81E-12
Dmel_CG12846	131.084	-2.264	0.302	-7.490	6.87E-14	8.44E-12
Dmel_CG6890	2345.639	1.977	0.264	7.476	7.65E-14	9.30E-12
Dmel_CG18405	1312.377	1.367	0.183	7.474	7.77E-14	9.33E-12
Dmel_CG1232	80.890	-3.916	0.524	-7.467	8.21E-14	9.76E-12
Dmel_CG31865	120.039	-2.432	0.327	-7.436	1.04E-13	1.22E-11
Dmel_CG32364	249.152	-2.558	0.344	-7.434	1.06E-13	1.23E-11
Dmel_CG11144	634.856	-1.765	0.239	-7.392	1.45E-13	1.67E-11
Dmel_CG1449	290.556	-2.023	0.275	-7.345	2.05E-13	2.33E-11

Dmel_CG7665	49.711	-2.544	0.352	-7.224	5.05E-13	5.68E-11
Dmel_CG6293	2110.091	2.582	0.358	7.217	5.33E-13	5.94E-11
Dmel_CG14204	32.050	2.302	0.321	7.173	7.33E-13	8.08E-11
Dmel_CG11354	35.882	-2.429	0.341	-7.125	1.04E-12	1.14E-10
Dmel_CG14253	132.269	2.126	0.299	7.123	1.06E-12	1.14E-10
Dmel_CG11099	22.666	-5.708	0.802	-7.116	1.11E-12	1.19E-10
Dmel_CG33837	2738.319	-2.233	0.314	-7.108	1.18E-12	1.19E-10
Dmel_CG33840	2738.319	-2.233	0.314	-7.108	1.18E-12	1.19E-10
Dmel_CG33843	2738.319	-2.233	0.314	-7.108	1.18E-12	1.19E-10
Dmel_CG33846	2738.319	-2.233	0.314	-7.108	1.18E-12	1.19E-10
Dmel_CG33849	2738.319	-2.233	0.314	-7.108	1.18E-12	1.19E-10
Dmel_CG33864	2738.319	-2.233	0.314	-7.108	1.18E-12	1.19E-10
Dmel_CG33655	51.654	3.304	0.467	7.074	1.50E-12	1.50E-10
Dmel_CG32490	168.640	-2.039	0.288	-7.073	1.52E-12	1.51E-10
Dmel_CR45446	23.752	5.109	0.724	7.056	1.71E-12	1.68E-10
Dmel_CG4587	91.859	-2.577	0.366	-7.044	1.87E-12	1.82E-10
Dmel_CG8034	387.147	-1.379	0.196	-7.030	2.06E-12	1.99E-10
Dmel_CG9981	21.353	-6.048	0.861	-7.027	2.11E-12	2.02E-10
Dmel_CG14102	550.744	1.420	0.203	7.003	2.50E-12	2.37E-10
Dmel_CG6424	1178.291	-1.063	0.152	-6.977	3.01E-12	2.82E-10
Dmel_CG12548	110.057	2.956	0.424	6.971	3.14E-12	2.92E-10
Dmel_CG31279	99.274	-2.625	0.377	-6.961	3.39E-12	3.13E-10
Dmel_CG15632	24.357	-6.024	0.867	-6.950	3.65E-12	3.33E-10
Dmel_CG14808	573.015	-2.613	0.376	-6.941	3.89E-12	3.52E-10
Dmel_CG14518	13.705	6.644	0.962	6.905	5.02E-12	4.48E-10
Dmel_CR46350	65.211	2.397	0.347	6.905	5.00E-12	4.48E-10
Dmel_CR32881	15.023	6.709	0.976	6.877	6.13E-12	5.42E-10
Dmel_CG5106	180.963	-2.039	0.298	-6.852	7.29E-12	6.39E-10
Dmel_CG4573	407.261	0.986	0.144	6.838	8.02E-12	6.97E-10
Dmel_CG15673	374.682	2.079	0.305	6.808	9.91E-12	8.55E-10
Dmel_CG32261	8.393	6.641	0.976	6.801	1.04E-11	8.87E-10
Dmel_CG32540	27.797	-5.848	0.863	-6.776	1.23E-11	1.05E-09

Dmel_CG40470	117.571	2.521	0.374	6.743	1.55E-11	1.30E-09
Dmel_CG10654	255.238	-1.959	0.291	-6.723	1.78E-11	1.48E-09
Dmel_CG32319	43.380	2.630	0.396	6.640	3.15E-11	2.61E-09
Dmel_CG13876	343.928	-1.331	0.202	-6.595	4.25E-11	3.47E-09
Dmel_CG17142	69.912	-1.977	0.300	-6.595	4.25E-11	3.47E-09
Dmel_CR44603	26.607	-6.925	1.050	-6.592	4.33E-11	3.51E-09
Dmel_CG13848	572.438	-1.490	0.226	-6.590	4.40E-11	3.53E-09
Dmel_CG4116	39.770	2.830	0.430	6.578	4.75E-11	3.79E-09
Dmel_CG8321	886.301	-1.178	0.181	-6.515	7.27E-11	5.76E-09
Dmel_CG2397	133.002	-1.613	0.248	-6.499	8.08E-11	6.36E-09
Dmel_CR44441	70.571	2.873	0.444	6.472	9.70E-11	7.57E-09
Dmel_CG4666	110.138	-2.471	0.383	-6.451	1.11E-10	8.62E-09
Dmel_CG32944	92.791	-2.040	0.317	-6.441	1.19E-10	9.12E-09
Dmel_CR44649	14.040	5.807	0.902	6.440	1.19E-10	9.12E-09
Dmel_CG3592	24.833	3.723	0.579	6.435	1.24E-10	9.39E-09
Dmel_CG15267	102.975	2.512	0.391	6.432	1.26E-10	9.51E-09
Dmel_CG4181	124.651	-2.626	0.408	-6.430	1.28E-10	9.54E-09
Dmel_CG33861	849.240	-2.803	0.438	-6.394	1.61E-10	1.20E-08
Dmel_CG5130	868.620	-1.343	0.212	-6.343	2.26E-10	1.67E-08
Dmel_CG42343	28.745	-2.528	0.399	-6.331	2.43E-10	1.78E-08
Dmel_CG12405	52.075	-1.899	0.300	-6.320	2.62E-10	1.90E-08
Dmel_CR34573	15.764	5.560	0.880	6.317	2.66E-10	1.92E-08
Dmel_CG14162	1349.763	1.374	0.219	6.270	3.61E-10	2.58E-08
Dmel_CG4500	7.493	6.350	1.013	6.270	3.60E-10	2.58E-08
Dmel_CG32204	31.301	-2.860	0.456	-6.268	3.66E-10	2.59E-08
Dmel_CG11372	1894.097	1.758	0.281	6.264	3.75E-10	2.62E-08
Dmel_CG3134	31.621	-3.101	0.495	-6.265	3.73E-10	2.62E-08
Dmel_CG11160	16.059	-5.132	0.820	-6.261	3.82E-10	2.66E-08
Dmel_CG3653	339.374	2.166	0.346	6.258	3.89E-10	2.68E-08
Dmel_CG3832	660.172	1.414	0.226	6.254	4.00E-10	2.74E-08
Dmel_CR31853	15.583	-6.169	0.987	-6.248	4.17E-10	2.84E-08
Dmel_CG46315	1065.167	-0.931	0.149	-6.227	4.75E-10	3.22E-08

Dmel_CG10062	88.652	-2.498	0.402	-6.217	5.06E-10	3.41E-08
Dmel_CG45019	491.689	-1.639	0.264	-6.213	5.19E-10	3.47E-08
Dmel_CG18495	1721.586	-1.927	0.311	-6.193	5.92E-10	3.93E-08
Dmel_CG14416	20.114	3.533	0.571	6.187	6.12E-10	4.04E-08
Dmel_CG30401	80.682	-2.540	0.412	-6.167	6.96E-10	4.56E-08
Dmel_CR44285	16.737	-4.535	0.738	-6.149	7.81E-10	5.09E-08
Dmel_CR44842	51.141	2.339	0.381	6.139	8.31E-10	5.39E-08
Dmel_CG6644	396.732	-2.312	0.377	-6.137	8.42E-10	5.41E-08
Dmel_CR43957	57.161	-2.135	0.348	-6.136	8.44E-10	5.41E-08
Dmel_CG30026	144.278	2.596	0.423	6.135	8.50E-10	5.41E-08
Dmel_CG11263	812.988	1.589	0.260	6.120	9.33E-10	5.91E-08
Dmel_CG17959	18.151	3.189	0.522	6.114	9.69E-10	6.10E-08
Dmel_CG32191	18.062	3.536	0.579	6.104	1.03E-09	6.45E-08
Dmel_CG15695	15.117	-5.448	0.899	-6.058	1.38E-09	8.58E-08
Dmel_CG8964	30.624	-2.560	0.424	-6.041	1.53E-09	9.48E-08
Dmel_CR46147	58.757	-1.968	0.326	-6.035	1.59E-09	9.77E-08
Dmel_CG13426	9.974	6.113	1.016	6.018	1.77E-09	1.08E-07
Dmel_CG6352	20.044	-3.623	0.602	-6.017	1.78E-09	1.08E-07
Dmel_CG4486	56.498	2.700	0.449	6.015	1.80E-09	1.09E-07
Dmel_CG6829	1109.723	1.216	0.204	5.975	2.30E-09	1.38E-07
Dmel_CG18766	417.316	1.249	0.209	5.962	2.49E-09	1.49E-07
Dmel_CG9475	65.197	-1.919	0.322	-5.959	2.53E-09	1.51E-07
Dmel_CG4381	408.162	-1.619	0.273	-5.941	2.84E-09	1.68E-07
Dmel_CG7863	307.615	1.102	0.186	5.935	2.94E-09	1.73E-07
Dmel_CG13540	16.816	-4.164	0.703	-5.924	3.14E-09	1.84E-07
Dmel_CG11940	4570.884	1.039	0.176	5.892	3.81E-09	2.21E-07
Dmel_CG45781	99.356	2.496	0.424	5.880	4.10E-09	2.37E-07
Dmel_CG1544	56.811	3.186	0.543	5.869	4.38E-09	2.52E-07
Dmel_CR41257	19.191	3.731	0.637	5.859	4.67E-09	2.67E-07
Dmel_CG13375	172.764	-1.742	0.298	-5.852	4.85E-09	2.76E-07
Dmel_CG7084	38.686	-3.722	0.638	-5.838	5.29E-09	2.99E-07
Dmel_CG12370	119.708	-2.318	0.398	-5.825	5.71E-09	3.20E-07

Dmel_CR43635	28.969	-2.640	0.453	-5.826	5.68E-09	3.20E-07
Dmel_CG3036	780.679	-1.187	0.204	-5.821	5.86E-09	3.27E-07
Dmel_CG13617	40.995	2.463	0.425	5.801	6.60E-09	3.66E-07
Dmel_CG42639	16.055	-4.825	0.832	-5.796	6.77E-09	3.73E-07
Dmel_CR44648	7.481	5.743	0.999	5.747	9.08E-09	4.98E-07
Dmel_CG32187	11.172	6.232	1.085	5.745	9.19E-09	5.02E-07
Dmel_CG10650	150.114	-1.962	0.342	-5.741	9.43E-09	5.12E-07
Dmel_CG17836	1929.733	-1.570	0.274	-5.732	9.94E-09	5.37E-07
Dmel_CR43605	66.687	2.061	0.360	5.719	1.07E-08	5.75E-07
Dmel_CG3588	32.672	2.561	0.448	5.716	1.09E-08	5.83E-07
Dmel_CG7433	4321.842	-0.989	0.174	-5.689	1.27E-08	6.78E-07
Dmel_CG9701	11.604	-4.237	0.747	-5.676	1.38E-08	7.27E-07
Dmel_CR32886	5281.077	-1.355	0.239	-5.676	1.38E-08	7.27E-07
Dmel_CG44193	1265.153	-1.120	0.198	-5.661	1.50E-08	7.84E-07
Dmel_CG9411	11.974	-4.948	0.874	-5.661	1.50E-08	7.84E-07
Dmel_CG12986	9.202	-5.950	1.054	-5.645	1.66E-08	8.60E-07
Dmel_CG42566	568.495	-2.099	0.372	-5.643	1.67E-08	8.62E-07
Dmel_CG18789	575.054	-1.270	0.226	-5.613	1.99E-08	1.02E-06
Dmel_CR42861	395.796	-1.322	0.236	-5.607	2.06E-08	1.06E-06
Dmel_CG10246	507.936	-1.118	0.200	-5.587	2.31E-08	1.17E-06
Dmel_CG2381	70.702	2.064	0.369	5.587	2.31E-08	1.17E-06
Dmel_CG8165	279.924	0.802	0.144	5.583	2.37E-08	1.19E-06
Dmel_CG42694	322.776	1.171	0.210	5.571	2.53E-08	1.27E-06
Dmel_CR43836	1370.913	1.125	0.202	5.567	2.58E-08	1.29E-06
Dmel_CG11280	82.750	1.787	0.322	5.545	2.94E-08	1.46E-06
Dmel_CG30046	65.427	1.783	0.322	5.539	3.05E-08	1.51E-06
Dmel_CR34151	2597.926	1.281	0.232	5.528	3.24E-08	1.60E-06
Dmel_CR44105	111.289	1.812	0.328	5.522	3.35E-08	1.65E-06
Dmel_CG3926	50.891	-2.071	0.375	-5.518	3.43E-08	1.68E-06
Dmel_CG15422	16.358	-3.229	0.589	-5.478	4.30E-08	2.08E-06
Dmel_CG7970	1818.632	-0.761	0.139	-5.478	4.31E-08	2.08E-06
Dmel_CR45600	123.977	-1.441	0.264	-5.466	4.62E-08	2.22E-06

Dmel_CR42871	50.583	-2.562	0.471	-5.439	5.36E-08	2.57E-06
Dmel_CG10962	178.967	2.521	0.464	5.433	5.55E-08	2.65E-06
Dmel_CR44370	668.992	1.879	0.348	5.400	6.65E-08	3.16E-06
Dmel_CG14591	15.962	-3.474	0.643	-5.399	6.71E-08	3.17E-06
Dmel_CG45002	81.687	2.435	0.451	5.397	6.79E-08	3.20E-06
Dmel_CR43887	48.400	-2.054	0.381	-5.391	6.99E-08	3.28E-06
Dmel_CG8193	9.669	-5.178	0.963	-5.378	7.53E-08	3.52E-06
Dmel_CG10693	63.837	-2.304	0.429	-5.370	7.89E-08	3.67E-06
Dmel_CG17657	833.649	0.932	0.174	5.368	7.97E-08	3.69E-06
Dmel_CG9922	1514.484	-1.367	0.255	-5.357	8.45E-08	3.89E-06
Dmel_CG4998	35.482	-2.912	0.545	-5.344	9.07E-08	4.16E-06
Dmel_CG14615	327.742	1.131	0.212	5.326	1.00E-07	4.58E-06
Dmel_CG17669	10.096	-4.037	0.759	-5.317	1.06E-07	4.80E-06
Dmel_CG16779	55.503	-2.362	0.445	-5.311	1.09E-07	4.93E-06
Dmel_CG3548	367.754	1.676	0.319	5.258	1.46E-07	6.58E-06
Dmel_CG1629	52.870	-1.812	0.345	-5.255	1.48E-07	6.66E-06
Dmel_CG8666	1194.885	-1.041	0.198	-5.247	1.55E-07	6.94E-06
Dmel_CG13780	102.811	-3.674	0.701	-5.241	1.60E-07	7.11E-06
Dmel_CG30048	8.917	-5.534	1.060	-5.222	1.77E-07	7.84E-06
Dmel_CG45544	25.811	-2.041	0.391	-5.219	1.80E-07	7.97E-06
Dmel_CG10390	88.576	-1.834	0.352	-5.206	1.93E-07	8.49E-06
Dmel_CR43461	15.553	-2.544	0.489	-5.205	1.94E-07	8.51E-06
Dmel_CG12535	350.714	-1.351	0.260	-5.192	2.08E-07	9.08E-06
Dmel_CG14026	3581.522	0.517	0.100	5.176	2.26E-07	9.83E-06
Dmel_CR43334	902.502	1.433	0.278	5.161	2.46E-07	1.07E-05
Dmel_CG31956	7.679	-5.030	0.975	-5.159	2.48E-07	1.07E-05
Dmel_CG8256	121.600	-1.630	0.317	-5.150	2.61E-07	1.12E-05
Dmel_CG14424	11.673	4.159	0.809	5.138	2.77E-07	1.18E-05
Dmel_CR45714	39.803	2.530	0.492	5.138	2.78E-07	1.18E-05
Dmel_CG5927	130.550	3.518	0.685	5.134	2.83E-07	1.20E-05
Dmel_CG42711	9.934	-5.047	0.984	-5.130	2.90E-07	1.23E-05
Dmel_CG5022	23.972	2.232	0.436	5.117	3.10E-07	1.31E-05

Dmel_CG10559	18.084	-2.548	0.498	-5.112	3.18E-07	1.33E-05
Dmel_CG42362	214.695	-1.187	0.232	-5.107	3.28E-07	1.37E-05
Dmel_CG42363	214.695	-1.187	0.232	-5.107	3.28E-07	1.37E-05
Dmel_CG10245	245.057	-1.401	0.275	-5.096	3.47E-07	1.44E-05
Dmel_CG12582	1823.184	0.794	0.156	5.095	3.48E-07	1.44E-05
Dmel_CG3346	377.229	1.460	0.287	5.090	3.59E-07	1.48E-05
Dmel_CG34313	123.277	-1.216	0.239	-5.082	3.73E-07	1.53E-05
Dmel_CR46488	96.924	2.110	0.416	5.077	3.83E-07	1.56E-05
Dmel_CR43283	44.621	4.945	0.975	5.074	3.90E-07	1.58E-05
Dmel_CG15705	4.557	5.773	1.138	5.072	3.94E-07	1.60E-05
Dmel_CG43798	14.265	-2.669	0.527	-5.068	4.02E-07	1.62E-05
Dmel_CG1851	132.622	-1.408	0.278	-5.063	4.12E-07	1.66E-05
Dmel_CG34329	9.708	-5.152	1.018	-5.060	4.20E-07	1.68E-05
Dmel_CG11186	154.593	2.035	0.403	5.044	4.55E-07	1.82E-05
Dmel_CG1631	6.321	-5.205	1.033	-5.039	4.69E-07	1.86E-05
Dmel_CG2671	15581.812	1.147	0.228	5.039	4.67E-07	1.86E-05
Dmel_CG10247	12.494	-3.256	0.647	-5.034	4.81E-07	1.90E-05
Dmel_CG44102	63.130	2.047	0.407	5.024	5.07E-07	1.99E-05
Dmel_CG44328	42.014	-2.179	0.436	-4.999	5.75E-07	2.25E-05
Dmel_CG3546	24.816	1.961	0.392	4.996	5.84E-07	2.28E-05
Dmel_CG3767	118.430	-1.757	0.352	-4.996	5.86E-07	2.28E-05
Dmel_CG10391	295.990	1.311	0.263	4.985	6.19E-07	2.40E-05
Dmel_CG32595	13.102	-3.220	0.647	-4.979	6.38E-07	2.46E-05
Dmel_CG31174	20.644	-1.919	0.386	-4.970	6.71E-07	2.58E-05
Dmel_CG3598	12.213	4.080	0.823	4.958	7.10E-07	2.72E-05
Dmel_CR34621	154.656	1.896	0.383	4.956	7.19E-07	2.75E-05
Dmel_CG10160	13.374	-3.030	0.613	-4.944	7.64E-07	2.90E-05
Dmel_CG10287	102.780	-1.638	0.331	-4.945	7.62E-07	2.90E-05
Dmel_CG11450	84.360	1.490	0.302	4.942	7.75E-07	2.93E-05
Dmel_CG31901	28.374	-3.474	0.703	-4.939	7.84E-07	2.95E-05
Dmel_CG8051	10.915	-2.609	0.529	-4.930	8.24E-07	3.09E-05
Dmel_CG45058	655.515	1.169	0.237	4.929	8.27E-07	3.09E-05

Dmel_CG31693	14.293	-3.563	0.724	-4.921	8.63E-07	3.22E-05
Dmel_CG3022	27.256	-2.367	0.482	-4.916	8.85E-07	3.29E-05
Dmel_CG30383	49.644	-1.689	0.344	-4.913	8.98E-07	3.32E-05
Dmel_CG13916	111.331	2.194	0.447	4.905	9.33E-07	3.44E-05
Dmel_CG11205	397.986	-1.798	0.368	-4.892	1.00E-06	3.67E-05
Dmel_CG32814	302.950	-1.768	0.362	-4.887	1.02E-06	3.75E-05
Dmel_CG10695	3201.672	-1.191	0.244	-4.886	1.03E-06	3.76E-05
Dmel_CG12763	27.517	-6.787	1.391	-4.878	1.07E-06	3.89E-05
Dmel_CR46284	5.583	-5.554	1.139	-4.878	1.07E-06	3.89E-05
Dmel_CG14173	20.836	2.596	0.533	4.872	1.11E-06	3.99E-05
Dmel_CG15083	836.327	-1.186	0.244	-4.861	1.17E-06	4.21E-05
Dmel_CG6502	1593.297	0.700	0.144	4.856	1.20E-06	4.31E-05
Dmel_CG14502	161.282	1.211	0.250	4.843	1.28E-06	4.57E-05
Dmel_CG11125	345.793	1.079	0.224	4.819	1.44E-06	5.14E-05
Dmel_CG17523	9.395	-2.831	0.588	-4.816	1.46E-06	5.19E-05
Dmel_CG31718	13.955	-2.733	0.568	-4.815	1.47E-06	5.21E-05
Dmel_CG13401	706.768	0.814	0.169	4.813	1.49E-06	5.24E-05
Dmel_CG8942	20.884	-3.571	0.742	-4.813	1.49E-06	5.24E-05
Dmel_CG34002	25.029	-2.108	0.439	-4.807	1.53E-06	5.38E-05
Dmel_CG34251	4.631	-5.622	1.173	-4.793	1.64E-06	5.74E-05
Dmel_CG12473	121.417	1.006	0.210	4.788	1.69E-06	5.86E-05
Dmel_CG12500	121.417	1.006	0.210	4.788	1.69E-06	5.86E-05
Dmel_CG30101	46.664	2.599	0.543	4.783	1.73E-06	5.99E-05
Dmel_CG10794	23.361	-6.086	1.273	-4.782	1.74E-06	5.99E-05
Dmel_CG32845	27.360	-2.062	0.431	-4.780	1.75E-06	6.02E-05
Dmel_CG18095	35.302	4.346	0.910	4.775	1.80E-06	6.12E-05
Dmel_CG6798	39.400	2.450	0.513	4.774	1.80E-06	6.12E-05
Dmel_CG8561	48.662	-2.040	0.427	-4.774	1.80E-06	6.12E-05
Dmel_CR45215	6.526	3.592	0.752	4.774	1.80E-06	6.12E-05
Dmel_CG13091	80.925	2.136	0.447	4.773	1.81E-06	6.14E-05
Dmel_CG31619	134.708	-1.970	0.413	-4.773	1.82E-06	6.14E-05
Dmel_CG17604	2294.672	1.027	0.215	4.769	1.85E-06	6.23E-05

Dmel_CG10089	69.275	1.575	0.331	4.758	1.96E-06	6.56E-05
Dmel_CG12730	69.324	1.436	0.303	4.742	2.11E-06	7.06E-05
Dmel_CG17885	29.019	-1.638	0.346	-4.739	2.15E-06	7.15E-05
Dmel_CG1343	8.774	-4.947	1.046	-4.730	2.24E-06	7.42E-05
Dmel_CG14420	15.867	2.736	0.578	4.731	2.24E-06	7.42E-05
Dmel_CG14489	4874.516	0.630	0.134	4.717	2.39E-06	7.89E-05
Dmel_CG4476	2247.740	1.223	0.259	4.714	2.43E-06	7.98E-05
Dmel_CG5976	1222.502	-1.007	0.214	-4.712	2.46E-06	8.06E-05
Dmel_CR34645	9.943	4.238	0.900	4.708	2.51E-06	8.20E-05
Dmel_CG33531	10.591	3.263	0.696	4.688	2.76E-06	9.02E-05
Dmel_CG2505	44.932	3.869	0.826	4.683	2.83E-06	9.21E-05
Dmel_CG9921	462.165	-1.269	0.271	-4.676	2.92E-06	9.46E-05
Dmel_CG4780	256.282	-1.154	0.247	-4.675	2.94E-06	9.48E-05
Dmel_CG32485	670.585	0.877	0.188	4.674	2.96E-06	9.51E-05
Dmel_CG33494	73.278	-2.457	0.526	-4.674	2.96E-06	9.51E-05
Dmel_CG14584	13.563	-2.618	0.561	-4.667	3.06E-06	9.80E-05
Dmel_CR46075	89.606	1.330	0.286	4.660	3.17E-06	1.01E-04
Dmel_CG4563	4.531	-5.693	1.222	-4.658	3.19E-06	1.02E-04
Dmel_CG11700	93.380	-1.222	0.263	-4.655	3.24E-06	1.03E-04
Dmel_CR32773	602.990	1.163	0.251	4.639	3.50E-06	1.11E-04
Dmel_CG42338	533.899	-1.121	0.242	-4.637	3.53E-06	1.11E-04
Dmel_CG1483	10242.605	1.798	0.389	4.626	3.73E-06	1.17E-04
Dmel_CG43867	1609.935	1.111	0.241	4.615	3.93E-06	1.23E-04
Dmel_CR46231	7.957	-5.437	1.179	-4.613	3.97E-06	1.24E-04
Dmel_CG32459	8.115	-3.873	0.840	-4.610	4.02E-06	1.25E-04
Dmel_CG17610	1299.127	0.816	0.177	4.604	4.14E-06	1.29E-04
Dmel_CG10816	140.617	-3.652	0.794	-4.601	4.21E-06	1.30E-04
Dmel_CG1464	109.018	1.495	0.325	4.601	4.20E-06	1.30E-04
Dmel_CR44833	413.121	-0.980	0.213	-4.601	4.21E-06	1.30E-04
Dmel_CG1922	81.028	2.023	0.440	4.596	4.32E-06	1.33E-04
Dmel_CG14356	31.409	2.251	0.490	4.594	4.36E-06	1.33E-04
Dmel_CG6986	148.456	-1.258	0.274	-4.594	4.35E-06	1.33E-04

Dmel_CG13636	2287.981	3.194	0.696	4.587	4.50E-06	1.37E-04
Dmel_CG8279	98.613	1.543	0.336	4.587	4.49E-06	1.37E-04
Dmel_CR34558	120.892	1.745	0.381	4.575	4.77E-06	1.45E-04
Dmel_CG2657	34.331	-2.251	0.492	-4.573	4.81E-06	1.45E-04
Dmel_CG46313	39.798	4.693	1.027	4.568	4.93E-06	1.48E-04
Dmel_CG30382	3253.403	-1.858	0.408	-4.552	5.31E-06	1.59E-04
Dmel_CG14686	5.867	-5.206	1.144	-4.550	5.35E-06	1.60E-04
Dmel_CG18568	26.606	1.474	0.324	4.549	5.39E-06	1.61E-04
Dmel_CG8023	8.117	-4.055	0.891	-4.549	5.39E-06	1.61E-04
Dmel_CG15347	570.453	-1.578	0.348	-4.531	5.88E-06	1.75E-04
Dmel_CG14948	111.422	-1.570	0.347	-4.521	6.17E-06	1.83E-04
Dmel_CG8825	1165.276	-1.305	0.289	-4.511	6.45E-06	1.91E-04
Dmel_CG3397	15.052	-2.800	0.621	-4.507	6.58E-06	1.94E-04
Dmel_CG11094	251.870	-1.553	0.345	-4.502	6.73E-06	1.98E-04
Dmel_CG9169	13.677	2.700	0.600	4.502	6.74E-06	1.98E-04
Dmel_CG11390	112.637	-2.243	0.498	-4.500	6.78E-06	1.98E-04
Dmel_CG43326	10.654	-2.837	0.631	-4.497	6.89E-06	2.01E-04
Dmel_CG10151	33.822	-2.032	0.452	-4.492	7.05E-06	2.05E-04
Dmel_CG9093	881.441	-0.757	0.169	-4.491	7.07E-06	2.05E-04
Dmel_CG2297	30.219	-2.497	0.556	-4.487	7.24E-06	2.09E-04
Dmel_CG14931	1536.983	1.612	0.359	4.485	7.29E-06	2.10E-04
Dmel_CG2528	8.511	-4.082	0.910	-4.485	7.30E-06	2.10E-04
Dmel_CG1092	40.559	-2.093	0.467	-4.481	7.44E-06	2.13E-04
Dmel_CG14688	934.841	-0.685	0.153	-4.476	7.60E-06	2.17E-04
Dmel_CG15820	1082.505	0.912	0.204	4.477	7.59E-06	2.17E-04
Dmel_CG43088	13.320	-3.076	0.689	-4.464	8.04E-06	2.29E-04
Dmel_CG4950	13.824	-3.830	0.858	-4.461	8.16E-06	2.31E-04
Dmel_CG3323	48.498	1.884	0.423	4.457	8.31E-06	2.35E-04
Dmel_CG33481	45.632	-1.894	0.425	-4.455	8.39E-06	2.36E-04
Dmel_CG8145	489.756	-0.916	0.206	-4.455	8.39E-06	2.36E-04
Dmel_CR43264	294.406	-1.474	0.331	-4.453	8.45E-06	2.37E-04
Dmel_CG11052	8.299	4.274	0.962	4.442	8.91E-06	2.50E-04

Dmel_CG1960	4602.892	0.621	0.140	4.441	8.96E-06	2.50E-04
Dmel_CG7727	134.263	1.172	0.265	4.429	9.48E-06	2.64E-04
Dmel_CG32165	1153.177	-1.572	0.355	-4.427	9.54E-06	2.65E-04
Dmel_CG9742	1047.738	-1.639	0.371	-4.423	9.75E-06	2.70E-04
Dmel_CG43366	29.454	-2.231	0.505	-4.420	9.89E-06	2.73E-04
Dmel_CG12470	17.654	-2.391	0.542	-4.408	1.04E-05	2.87E-04
Dmel_CG6128	36.042	-2.440	0.554	-4.403	1.07E-05	2.94E-04
Dmel_CG6698	17.300	-3.573	0.812	-4.401	1.08E-05	2.96E-04
Dmel_CG1443	162.341	1.837	0.418	4.393	1.12E-05	3.05E-04
Dmel_CG41520	200.878	1.460	0.332	4.392	1.12E-05	3.07E-04
Dmel_CG18437	12.750	-2.891	0.660	-4.380	1.19E-05	3.24E-04
Dmel_CR34575	10.820	2.843	0.650	4.377	1.20E-05	3.27E-04
Dmel_CG7391	327.485	1.327	0.304	4.365	1.27E-05	3.44E-04
Dmel_CG5080	19.507	-2.449	0.562	-4.355	1.33E-05	3.60E-04
Dmel_CG14907	262.745	-1.083	0.249	-4.348	1.37E-05	3.70E-04
Dmel_CG10638	1166.946	-0.953	0.219	-4.343	1.40E-05	3.77E-04
Dmel_CG42829	4.622	-5.282	1.218	-4.337	1.45E-05	3.88E-04
Dmel_CG7918	20.384	2.296	0.530	4.334	1.46E-05	3.91E-04
Dmel_CR32914	35.179	-1.441	0.333	-4.331	1.49E-05	3.97E-04
Dmel_CG42368	30.697	1.790	0.414	4.319	1.57E-05	4.17E-04
Dmel_CG10936	307.998	1.283	0.297	4.313	1.61E-05	4.28E-04
Dmel_CG14423	8.974	3.751	0.872	4.299	1.71E-05	4.54E-04
Dmel_CR43989	55.193	-1.304	0.304	-4.297	1.73E-05	4.57E-04
Dmel_CR44183	4.078	4.873	1.134	4.296	1.74E-05	4.58E-04
Dmel_CG6044	14.793	2.702	0.630	4.289	1.79E-05	4.71E-04
Dmel_CG2812	61.353	-1.769	0.413	-4.287	1.81E-05	4.75E-04
Dmel_CG1506	66.867	-1.574	0.368	-4.273	1.93E-05	5.04E-04
Dmel_CG4620	4335.134	0.799	0.187	4.268	1.97E-05	5.14E-04
Dmel_CG7549	52.479	-1.357	0.318	-4.265	2.00E-05	5.20E-04
Dmel_CG31519	27.150	-2.340	0.550	-4.252	2.12E-05	5.50E-04
Dmel_CG42281	10048.584	0.592	0.139	4.251	2.13E-05	5.50E-04
Dmel_CR43302	6.039	3.219	0.758	4.248	2.16E-05	5.57E-04

Dmel_CG17077	839.671	0.783	0.186	4.204	2.63E-05	6.77E-04
Dmel_CG11961	335.734	-0.664	0.158	-4.201	2.66E-05	6.84E-04
Dmel_CG1969	470.276	-0.499	0.119	-4.186	2.83E-05	7.27E-04
Dmel_CG5399	18.394	-2.327	0.557	-4.180	2.92E-05	7.47E-04
Dmel_CG1873	30.188	-2.071	0.496	-4.176	2.97E-05	7.57E-04
Dmel_CG32017	173.619	1.439	0.345	4.174	3.00E-05	7.63E-04
Dmel_CG12749	4132.742	-0.630	0.151	-4.169	3.07E-05	7.79E-04
Dmel_CG7152	41.498	1.793	0.430	4.168	3.08E-05	7.80E-04
Dmel_CG31601	12.167	-3.060	0.735	-4.163	3.13E-05	7.92E-04
Dmel_CR18854	838.629	0.990	0.238	4.163	3.14E-05	7.92E-04
Dmel_CG9580	35.037	-1.839	0.442	-4.162	3.15E-05	7.93E-04
Dmel_CG31618	2229.642	-0.953	0.229	-4.154	3.26E-05	8.07E-04
Dmel_CG33814	2229.642	-0.953	0.229	-4.154	3.26E-05	8.07E-04
Dmel_CG33817	2229.642	-0.953	0.229	-4.154	3.26E-05	8.07E-04
Dmel_CG33820	2229.642	-0.953	0.229	-4.154	3.26E-05	8.07E-04
Dmel_CG33823	2229.642	-0.953	0.229	-4.154	3.26E-05	8.07E-04
Dmel_CG33826	2229.642	-0.953	0.229	-4.154	3.26E-05	8.07E-04
Dmel_CG33829	2229.642	-0.953	0.229	-4.154	3.26E-05	8.07E-04
Dmel_CG5792	3509.804	0.710	0.171	4.152	3.29E-05	8.12E-04
Dmel_CR45923	329.714	1.048	0.252	4.152	3.30E-05	8.12E-04
Dmel_CG11714	5.974	-3.867	0.934	-4.141	3.46E-05	8.50E-04
Dmel_CG8638	7.909	-2.815	0.680	-4.139	3.49E-05	8.56E-04
Dmel_CR43609	35.121	-2.292	0.554	-4.138	3.51E-05	8.58E-04
Dmel_CG10723	53.062	-1.426	0.345	-4.134	3.57E-05	8.69E-04
Dmel_CG5322	43.751	-2.396	0.580	-4.134	3.56E-05	8.69E-04
Dmel_CG9380	914.006	2.463	0.596	4.130	3.62E-05	8.80E-04
Dmel_CG34360	2556.488	0.835	0.202	4.129	3.64E-05	8.84E-04
Dmel_CR44366	13.310	-1.815	0.440	-4.122	3.76E-05	9.11E-04
Dmel_CG7395	11.002	2.961	0.719	4.120	3.79E-05	9.16E-04
Dmel_CR43898	11.698	2.122	0.515	4.116	3.85E-05	9.27E-04
Dmel_CG1004	121.543	1.633	0.397	4.112	3.93E-05	9.44E-04
Dmel_CG31288	22.525	-2.495	0.609	-4.095	4.21E-05	1.01E-03

Dmel_CG10877	88.197	-1.683	0.411	-4.093	4.26E-05	1.02E-03
Dmel_CR32896	10.122	-3.330	0.814	-4.093	4.26E-05	1.02E-03
Dmel_CG17681	14.570	-2.303	0.563	-4.090	4.31E-05	1.03E-03
Dmel_CG5338	70.237	-2.034	0.498	-4.086	4.39E-05	1.04E-03
Dmel_CG15143	100.498	1.533	0.375	4.083	4.44E-05	1.05E-03
Dmel_CG6067	10.429	-3.312	0.811	-4.084	4.43E-05	1.05E-03
Dmel_CG7882	16.945	-4.855	1.190	-4.080	4.51E-05	1.07E-03
Dmel_CG14640	9.690	-2.996	0.735	-4.075	4.60E-05	1.08E-03
Dmel_CG30170	20.719	-2.277	0.560	-4.065	4.80E-05	1.13E-03
Dmel_CG14606	111.001	4.228	1.042	4.059	4.92E-05	1.15E-03
Dmel_CG42486	7.794	-2.105	0.520	-4.053	5.06E-05	1.18E-03
Dmel_CR44914	34.591	2.238	0.552	4.051	5.09E-05	1.19E-03
Dmel_CG15599	55.457	-2.241	0.553	-4.050	5.13E-05	1.20E-03
Dmel_CG17572	7.733	2.997	0.743	4.032	5.52E-05	1.28E-03
Dmel_CG33779	7.876	2.716	0.674	4.032	5.54E-05	1.28E-03
Dmel_CG9907	59.516	-1.325	0.329	-4.031	5.56E-05	1.29E-03
Dmel_CG32313	7.303	3.037	0.754	4.029	5.60E-05	1.29E-03
Dmel_CG3008	1125.630	-0.785	0.195	-4.025	5.69E-05	1.31E-03
Dmel_CG32082	143.362	-1.201	0.299	-4.012	6.03E-05	1.38E-03
Dmel_CG32381	6.858	-3.414	0.851	-4.012	6.02E-05	1.38E-03
Dmel_CR45199	15.610	2.262	0.564	4.011	6.05E-05	1.38E-03
Dmel_CR45580	8.928	-1.996	0.499	-4.003	6.26E-05	1.43E-03
Dmel_CG3857	518.727	0.838	0.210	3.997	6.43E-05	1.47E-03
Dmel_CG15034	80.952	1.369	0.343	3.991	6.59E-05	1.50E-03
Dmel_CG1683	61.082	-1.424	0.357	-3.988	6.67E-05	1.51E-03
Dmel_CG15598	8.615	2.751	0.690	3.985	6.75E-05	1.53E-03
Dmel_CG43389	9.534	-2.635	0.664	-3.969	7.21E-05	1.63E-03
Dmel_CG7149	511.711	0.872	0.220	3.958	7.55E-05	1.70E-03
Dmel_CG7383	5.086	-4.596	1.162	-3.956	7.62E-05	1.72E-03
Dmel_CG12477	8.616	-4.050	1.027	-3.944	8.01E-05	1.80E-03
Dmel_CG6125	90.955	-1.593	0.404	-3.944	8.03E-05	1.80E-03
Dmel_CR45029	87.357	1.013	0.257	3.944	8.02E-05	1.80E-03

Dmel_CG32365	2522.157	0.876	0.222	3.939	8.17E-05	1.82E-03
Dmel_CG43395	5.634	-3.479	0.884	-3.937	8.24E-05	1.84E-03
Dmel_CG32793	17.470	2.676	0.680	3.933	8.38E-05	1.86E-03
Dmel_CG9738	2394.475	-0.619	0.157	-3.933	8.39E-05	1.86E-03
Dmel_CG17570	6.795	2.553	0.650	3.931	8.47E-05	1.87E-03
Dmel_CG31262	1828.361	0.790	0.201	3.930	8.49E-05	1.88E-03
Dmel_CG11155	1048.739	-1.266	0.323	-3.917	8.97E-05	1.98E-03
Dmel_CG34431	161.743	-1.020	0.261	-3.910	9.22E-05	2.03E-03
Dmel_CG34031	23.601	-2.008	0.514	-3.905	9.43E-05	2.07E-03
Dmel_CG9780	15.302	-2.148	0.550	-3.902	9.54E-05	2.09E-03
Dmel_CR46268	170.251	-1.026	0.263	-3.901	9.58E-05	2.10E-03
Dmel_CG14643	29.737	-2.214	0.568	-3.897	9.75E-05	2.13E-03
Dmel_CG7542	10.141	2.826	0.726	3.892	9.92E-05	2.16E-03
Dmel_CG42598	119.404	3.509	0.902	3.891	1.00E-04	2.17E-03
Dmel_CG14616	5403.289	0.544	0.140	3.890	1.00E-04	2.17E-03
Dmel_CG17907	42.096	-1.423	0.366	-3.887	1.01E-04	2.19E-03
Dmel_CG3448	358.908	-0.949	0.244	-3.888	1.01E-04	2.19E-03
Dmel_CG42335	49.366	4.755	1.224	3.884	1.03E-04	2.21E-03
Dmel_CG15150	6.838	-3.981	1.025	-3.884	1.03E-04	2.21E-03
Dmel_CR42254	371.532	0.984	0.254	3.881	1.04E-04	2.24E-03
Dmel_CG10091	216.140	-1.617	0.417	-3.876	1.06E-04	2.27E-03
Dmel_CG3796	20.569	-1.603	0.414	-3.873	1.08E-04	2.30E-03
Dmel_CR45334	5.283	-3.658	0.946	-3.869	1.09E-04	2.34E-03
Dmel_CR42910	65.924	1.696	0.439	3.866	1.11E-04	2.36E-03
Dmel_CG14639	7.664	-3.024	0.783	-3.863	1.12E-04	2.37E-03
Dmel_CR42743	4.749	-4.446	1.151	-3.863	1.12E-04	2.37E-03
Dmel_CR44348	10.906	2.269	0.588	3.862	1.13E-04	2.39E-03
Dmel_CG33658	6.283	4.239	1.100	3.855	1.16E-04	2.45E-03
Dmel_CG5392	15.491	-1.899	0.493	-3.850	1.18E-04	2.49E-03
Dmel_CR44953	22.110	-1.841	0.479	-3.845	1.21E-04	2.54E-03
Dmel_CG33111	598.892	0.612	0.159	3.841	1.23E-04	2.58E-03
Dmel_CG14933	32.287	-2.019	0.526	-3.839	1.24E-04	2.59E-03

Dmel_CG31801	8.478	-2.242	0.585	-3.833	1.27E-04	2.65E-03
Dmel_CR45124	51.575	1.671	0.436	3.832	1.27E-04	2.65E-03
Dmel_CG12910	6.836	3.245	0.847	3.832	1.27E-04	2.65E-03
Dmel_CG31015	538.599	1.076	0.281	3.831	1.28E-04	2.66E-03
Dmel_CG1743	114.738	-1.366	0.357	-3.827	1.29E-04	2.69E-03
Dmel_CG8003	1339.184	0.620	0.162	3.825	1.31E-04	2.71E-03
Dmel_CG6701	6459.136	0.625	0.163	3.823	1.32E-04	2.72E-03
Dmel_CG4608	113.986	-1.184	0.310	-3.821	1.33E-04	2.75E-03
Dmel_CG12410	544.961	0.593	0.155	3.817	1.35E-04	2.77E-03
Dmel_CG44835	2748.099	0.677	0.177	3.818	1.35E-04	2.77E-03
Dmel_CG6324	34.299	-1.676	0.439	-3.818	1.34E-04	2.77E-03
Dmel_CG9472	10.918	2.066	0.542	3.812	1.38E-04	2.82E-03
Dmel_CG13793	53.229	1.552	0.408	3.808	1.40E-04	2.86E-03
Dmel_CG14757	107.605	0.895	0.235	3.802	1.44E-04	2.93E-03
Dmel_CG18408	587.102	0.741	0.195	3.799	1.45E-04	2.94E-03
Dmel_CG34357	51.034	1.339	0.352	3.800	1.45E-04	2.94E-03
Dmel_CG5455	372.518	-1.046	0.275	-3.800	1.45E-04	2.94E-03
Dmel_CG11356	13.659	-2.148	0.566	-3.794	1.48E-04	2.99E-03
Dmel_CR45577	14.166	2.232	0.588	3.795	1.48E-04	2.99E-03
Dmel_CG5644	28.092	1.858	0.491	3.787	1.52E-04	3.08E-03
Dmel_CG42813	353.216	1.405	0.371	3.787	1.53E-04	3.08E-03
Dmel_CR44230	13.634	-2.024	0.535	-3.784	1.54E-04	3.10E-03
Dmel_CG16778	54.657	2.348	0.621	3.779	1.57E-04	3.15E-03
Dmel_CG8588	597.074	1.012	0.268	3.774	1.61E-04	3.22E-03
Dmel_CR43242	104.596	1.334	0.354	3.772	1.62E-04	3.23E-03
Dmel_CR34626	1126.535	1.548	0.411	3.768	1.64E-04	3.28E-03
Dmel_CG41434	7.270	-3.616	0.961	-3.762	1.68E-04	3.35E-03
Dmel_CG8274	6007.375	-0.557	0.148	-3.762	1.69E-04	3.35E-03
Dmel_CG3104	56.006	-1.293	0.344	-3.758	1.71E-04	3.40E-03
Dmel_CG14062	3.626	-4.929	1.313	-3.754	1.74E-04	3.44E-03
Dmel_CG42294	6.836	-3.208	0.855	-3.752	1.75E-04	3.47E-03
Dmel_CG13604	682.846	0.784	0.209	3.749	1.78E-04	3.50E-03

Dmel_CG13970	20.195	-2.312	0.617	-3.748	1.78E-04	3.51E-03
Dmel_CG11049	56.984	-1.611	0.430	-3.743	1.82E-04	3.57E-03
Dmel_CG1894	11.141	-2.082	0.557	-3.735	1.88E-04	3.68E-03
Dmel_CG10469	24.417	-1.430	0.383	-3.730	1.92E-04	3.76E-03
Dmel_CG4260	7777.907	0.530	0.142	3.727	1.93E-04	3.78E-03
Dmel_CG10868	20270.917	0.732	0.197	3.726	1.95E-04	3.80E-03
Dmel_CG9968	1175.027	-0.709	0.191	-3.710	2.07E-04	4.03E-03
Dmel_CG12800	317.337	-1.068	0.288	-3.705	2.11E-04	4.11E-03
Dmel_CG10776	280.046	0.841	0.228	3.687	2.26E-04	4.40E-03
Dmel_CG31477	8.939	-2.314	0.631	-3.669	2.43E-04	4.71E-03
Dmel_CG31721	43.300	-1.874	0.511	-3.669	2.43E-04	4.71E-03
Dmel_CG30000	890.667	-1.008	0.275	-3.665	2.48E-04	4.78E-03
Dmel_CG10440	6.298	2.592	0.708	3.662	2.50E-04	4.81E-03
Dmel_CG18522	301.389	-1.437	0.392	-3.662	2.50E-04	4.81E-03
Dmel_CG4209	16.847	-1.945	0.531	-3.661	2.51E-04	4.82E-03
Dmel_CG42316	192.031	-1.525	0.417	-3.658	2.54E-04	4.88E-03
Dmel_CG13772	15.539	-2.247	0.614	-3.657	2.55E-04	4.88E-03
Dmel_CG9339	7193.178	0.491	0.134	3.657	2.55E-04	4.88E-03
Dmel_CG12662	86.071	1.509	0.413	3.656	2.56E-04	4.89E-03
Dmel_CG3359	1017.402	-0.873	0.239	-3.654	2.58E-04	4.91E-03
Dmel_CG2679	77.051	0.973	0.266	3.654	2.59E-04	4.91E-03
Dmel_CG12190	1070.714	0.942	0.258	3.652	2.60E-04	4.92E-03
Dmel_CG15879	82.055	0.980	0.268	3.652	2.60E-04	4.92E-03
Dmel_CG17352	58.907	2.070	0.567	3.650	2.62E-04	4.96E-03
Dmel_CG9165	1988.485	-1.065	0.292	-3.647	2.65E-04	5.01E-03
Dmel_CR45510	7.744	-3.035	0.833	-3.645	2.67E-04	5.03E-03
Dmel_CG31436	22.798	-1.887	0.519	-3.639	2.74E-04	5.14E-03
Dmel_CG40305	50.491	1.110	0.305	3.639	2.74E-04	5.14E-03
Dmel_CG2641	219.749	-0.803	0.221	-3.637	2.76E-04	5.17E-03
Dmel_CG33474	26.760	-1.370	0.377	-3.634	2.79E-04	5.22E-03
Dmel_CG34120	382.219	-0.954	0.263	-3.631	2.82E-04	5.26E-03
Dmel_CG8606	869.507	0.588	0.162	3.624	2.90E-04	5.40E-03

Dmel_CG6232	43.952	-1.009	0.279	-3.622	2.92E-04	5.43E-03
Dmel_CG34411	5.697	-3.139	0.868	-3.617	2.98E-04	5.53E-03
Dmel_CG2985	15846.085	-1.395	0.386	-3.613	3.03E-04	5.60E-03
Dmel_CG33855	661.466	1.268	0.351	3.613	3.03E-04	5.60E-03
Dmel_CG33858	661.466	1.268	0.351	3.613	3.03E-04	5.60E-03
Dmel_CG18313	6.700	2.594	0.719	3.609	3.07E-04	5.67E-03
Dmel_CG34325	218.050	-1.043	0.289	-3.608	3.08E-04	5.67E-03
Dmel_CG43749	68.898	-1.747	0.484	-3.608	3.08E-04	5.67E-03
Dmel_CR45822	59.722	1.856	0.515	3.606	3.11E-04	5.71E-03
Dmel_CG31300	10.149	-2.756	0.765	-3.603	3.15E-04	5.77E-03
Dmel_CG15828	114.025	-2.543	0.706	-3.601	3.17E-04	5.79E-03
Dmel_CG7565	530.624	0.618	0.172	3.588	3.33E-04	6.08E-03
Dmel_CG13624	4837.368	0.626	0.175	3.587	3.35E-04	6.10E-03
Dmel_CR45472	12.276	2.189	0.611	3.584	3.38E-04	6.15E-03
Dmel_CG1532	1363.500	-0.736	0.205	-3.581	3.43E-04	6.22E-03
Dmel_CG1971	113.016	-0.924	0.258	-3.580	3.44E-04	6.23E-03
Dmel_CG31687	689.422	-0.814	0.228	-3.577	3.47E-04	6.28E-03
Dmel_CG7737	406.711	-0.897	0.251	-3.576	3.49E-04	6.31E-03
Dmel_CG3837	293.345	0.924	0.258	3.574	3.51E-04	6.34E-03
Dmel_CG13659	14.223	-1.963	0.550	-3.566	3.62E-04	6.52E-03
Dmel_CG8819	502.091	-0.591	0.166	-3.566	3.63E-04	6.52E-03
Dmel_CG12099	2772.921	-0.642	0.180	-3.563	3.67E-04	6.57E-03
Dmel_CG9068	9.058	3.563	1.000	3.563	3.67E-04	6.57E-03
Dmel_CG10481	27.823	-1.465	0.412	-3.559	3.72E-04	6.66E-03
Dmel_CG14307	1087.564	0.958	0.269	3.557	3.75E-04	6.70E-03
Dmel_CG31918	958.032	-0.742	0.209	-3.551	3.83E-04	6.83E-03
Dmel_CG32354	36.624	-1.592	0.448	-3.551	3.84E-04	6.83E-03
Dmel_CR46216	14.209	-2.633	0.741	-3.551	3.84E-04	6.83E-03
Dmel_CG14472	25993.883	0.315	0.089	3.549	3.87E-04	6.86E-03
Dmel_CG7466	1646.183	-0.502	0.142	-3.539	4.02E-04	7.10E-03
Dmel_CR43303	10.440	2.706	0.765	3.539	4.02E-04	7.10E-03
Dmel_CG18102	3933.133	0.395	0.112	3.538	4.03E-04	7.11E-03

Dmel_CG9652	17.219	1.772	0.501	3.537	4.05E-04	7.13E-03
Dmel_CG12066	170.368	-0.917	0.259	-3.535	4.07E-04	7.16E-03
Dmel_CG45057	104.242	-1.329	0.376	-3.532	4.12E-04	7.23E-03
Dmel_CG9925	8017.743	0.506	0.143	3.530	4.15E-04	7.28E-03
Dmel_CG2759	71.072	-1.197	0.339	-3.528	4.18E-04	7.33E-03
Dmel_CG3159	17.200	-1.981	0.562	-3.527	4.21E-04	7.35E-03
Dmel_CG40006	2376.258	0.767	0.217	3.526	4.21E-04	7.35E-03
Dmel_CG3329	5252.860	-0.707	0.201	-3.522	4.28E-04	7.45E-03
Dmel_CR45260	5.707	4.054	1.153	3.517	4.37E-04	7.60E-03
Dmel_CR44330	9.131	2.344	0.667	3.516	4.38E-04	7.60E-03
Dmel_CG15784	293.016	-1.201	0.342	-3.513	4.43E-04	7.67E-03
Dmel_CG9390	2169.006	-0.876	0.249	-3.513	4.43E-04	7.67E-03
Dmel_CG43921	462.043	0.947	0.270	3.511	4.47E-04	7.72E-03
Dmel_CG43052	15.089	1.909	0.544	3.508	4.52E-04	7.79E-03
Dmel_CG17292	1474.083	0.696	0.199	3.503	4.59E-04	7.91E-03
Dmel_CG13384	2664.424	0.482	0.138	3.500	4.65E-04	8.00E-03
Dmel_CG3565	6.277	-3.306	0.945	-3.499	4.67E-04	8.01E-03
Dmel_CG10833	21.891	-4.331	1.239	-3.496	4.72E-04	8.10E-03
Dmel_CG13539	9.683	2.242	0.642	3.495	4.74E-04	8.12E-03
Dmel_CG3879	12.492	-2.007	0.575	-3.491	4.81E-04	8.21E-03
Dmel_CG13140	9.747	-1.950	0.559	-3.485	4.93E-04	8.37E-03
Dmel_CG3209	1529.199	0.411	0.118	3.485	4.93E-04	8.37E-03
Dmel_CG4472	34.227	-1.331	0.382	-3.485	4.93E-04	8.37E-03
Dmel_CG7874	21.781	-4.907	1.408	-3.486	4.91E-04	8.37E-03
Dmel_CG34141	119.156	0.971	0.279	3.477	5.08E-04	8.60E-03
Dmel_CG31866	314.021	0.646	0.186	3.475	5.10E-04	8.63E-03
Dmel_CR44793	9.708	-2.928	0.843	-3.474	5.12E-04	8.65E-03
Dmel_CG7997	780.900	0.530	0.153	3.474	5.13E-04	8.65E-03
Dmel_CR34635	8.396	2.244	0.646	3.473	5.15E-04	8.67E-03
Dmel_CG16956	123.954	1.533	0.442	3.466	5.28E-04	8.87E-03
Dmel_CG3929	5193.590	-0.960	0.277	-3.463	5.35E-04	8.98E-03
Dmel_CG9901	6113.781	0.361	0.104	3.462	5.36E-04	8.98E-03

Dmel_CG6618	40.165	1.385	0.401	3.456	5.48E-04	9.17E-03
Dmel_CR43607	38.001	1.008	0.292	3.454	5.52E-04	9.22E-03
Dmel_CG17527	10.179	-2.272	0.658	-3.453	5.55E-04	9.27E-03
Dmel_CR44024	22.499	1.792	0.520	3.447	5.67E-04	9.45E-03
Dmel_CG3091	43.997	-1.541	0.447	-3.445	5.70E-04	9.49E-03
Dmel_CG7644	30.473	-1.781	0.517	-3.445	5.71E-04	9.49E-03
Dmel_CR45789	14.109	1.924	0.560	3.438	5.86E-04	9.72E-03
Dmel_CG30385	5.769	-2.845	0.829	-3.433	5.96E-04	9.84E-03
Dmel_CG4995	6.334	-2.402	0.700	-3.433	5.97E-04	9.84E-03
Dmel_CG6704	461.899	1.126	0.328	3.433	5.96E-04	9.84E-03
Dmel_CR43949	41.829	1.450	0.422	3.433	5.97E-04	9.84E-03
Dmel_CG10241	394.726	1.066	0.311	3.430	6.03E-04	9.87E-03
Dmel_CG10683	1174.214	0.730	0.213	3.431	6.02E-04	9.87E-03
Dmel_CG13516	62.853	-1.387	0.404	-3.430	6.03E-04	9.87E-03
Dmel_CG32350	8070.134	0.783	0.228	3.430	6.03E-04	9.87E-03
Dmel_CR32875	15.889	2.368	0.690	3.431	6.02E-04	9.87E-03
Dmel_CG10334	1524.281	0.510	0.149	3.430	6.04E-04	9.87E-03
Dmel_CG8595	16.500	1.613	0.471	3.428	6.08E-04	9.91E-03
Dmel_CG14898	511.421	0.874	0.255	3.425	6.15E-04	1.00E-02
Dmel_CG44246	646.353	0.760	0.222	3.423	6.20E-04	1.01E-02
Dmel_CG7224	1716.811	0.836	0.244	3.421	6.24E-04	1.01E-02
Dmel_CG15269	8.248	-3.161	0.924	-3.420	6.26E-04	1.01E-02
Dmel_CG40160	3039.300	0.735	0.215	3.418	6.31E-04	1.02E-02
Dmel_CR42452	1770.822	1.031	0.302	3.417	6.34E-04	1.02E-02
Dmel_CG9919	9.509	-2.393	0.701	-3.415	6.38E-04	1.03E-02
Dmel_CG16896	1701.150	0.433	0.127	3.412	6.46E-04	1.04E-02
Dmel_CG6604	39.186	1.492	0.437	3.412	6.46E-04	1.04E-02
Dmel_CG3314	19145.955	-1.176	0.345	-3.411	6.48E-04	1.04E-02
Dmel_CG14218	17.215	2.344	0.688	3.407	6.56E-04	1.05E-02
Dmel_CG13982	106.094	-1.105	0.324	-3.407	6.58E-04	1.05E-02
Dmel_CG10514	44.024	-3.242	0.952	-3.404	6.64E-04	1.06E-02
Dmel_CG4859	83.161	-1.139	0.335	-3.404	6.65E-04	1.06E-02

Dmel_CG18507	93.141	1.294	0.381	3.401	6.71E-04	1.07E-02
Dmel_CG8550	20.847	-2.053	0.604	-3.402	6.69E-04	1.07E-02
Dmel_CG9353	452.173	-1.013	0.298	-3.401	6.70E-04	1.07E-02
Dmel_CR43589	107.488	1.689	0.497	3.400	6.75E-04	1.07E-02
Dmel_CG14995	758.069	0.822	0.242	3.397	6.81E-04	1.08E-02
Dmel_CG42606	8.504	2.328	0.685	3.397	6.80E-04	1.08E-02
Dmel_CG11284	4635.783	0.581	0.171	3.390	7.00E-04	1.11E-02
Dmel_CG12342	308.203	1.065	0.314	3.389	7.01E-04	1.11E-02
Dmel_CG42365	650.988	-0.730	0.215	-3.387	7.05E-04	1.11E-02
Dmel_CG5096	38.054	-1.443	0.427	-3.382	7.18E-04	1.13E-02
Dmel_CG3984	9.224	3.722	1.101	3.382	7.20E-04	1.13E-02
Dmel_CR44756	369.555	0.923	0.273	3.378	7.30E-04	1.15E-02
Dmel_CG4700	8378.432	0.563	0.167	3.376	7.37E-04	1.15E-02
Dmel_CG9177	13081.628	0.723	0.214	3.375	7.38E-04	1.15E-02
Dmel_CG6718	2868.486	0.799	0.237	3.374	7.40E-04	1.16E-02
Dmel_CG46440	920.684	0.589	0.175	3.374	7.42E-04	1.16E-02
Dmel_CG18131	15.976	-1.772	0.525	-3.372	7.45E-04	1.16E-02
Dmel_CR44472	23.272	1.695	0.503	3.373	7.44E-04	1.16E-02
Dmel_CG8891	326.888	-1.078	0.320	-3.369	7.55E-04	1.17E-02
Dmel_CG43346	278.496	-1.055	0.313	-3.368	7.58E-04	1.17E-02
Dmel_CG14053	287.028	0.654	0.194	3.366	7.64E-04	1.18E-02
Dmel_CG4484	163.176	1.219	0.362	3.363	7.72E-04	1.19E-02
Dmel_CG7449	30.833	1.278	0.380	3.361	7.75E-04	1.20E-02
Dmel_CR45171	45.239	-1.316	0.392	-3.359	7.82E-04	1.20E-02
Dmel_CG31075	302.346	-1.178	0.351	-3.358	7.85E-04	1.21E-02
Dmel_CG31495	548.374	-0.665	0.198	-3.358	7.85E-04	1.21E-02
Dmel_CG2239	26.138	-1.554	0.463	-3.354	7.96E-04	1.22E-02
Dmel_CR34555	357.248	1.901	0.567	3.352	8.03E-04	1.23E-02
Dmel_CG30361	21.616	1.814	0.541	3.351	8.04E-04	1.23E-02
Dmel_CG5370	1911.098	-0.532	0.159	-3.349	8.11E-04	1.24E-02
Dmel_CG9331	286.658	-0.670	0.200	-3.347	8.17E-04	1.25E-02
Dmel_CG9173	16.362	1.790	0.535	3.346	8.19E-04	1.25E-02

Dmel_CG12789	42.944	-1.243	0.371	-3.345	8.22E-04	1.25E-02
Dmel_CG9586	483.605	-0.903	0.270	-3.344	8.25E-04	1.25E-02
Dmel_CR34531	17.468	-2.141	0.641	-3.343	8.30E-04	1.26E-02
Dmel_CG34235	13.980	1.569	0.470	3.341	8.34E-04	1.26E-02
Dmel_CG13855	5.891	-3.022	0.905	-3.339	8.41E-04	1.27E-02
Dmel_CG10315	393.534	-0.984	0.295	-3.333	8.58E-04	1.30E-02
Dmel_CG13833	4.924	-3.643	1.093	-3.332	8.61E-04	1.30E-02
Dmel_CG8389	831.413	0.536	0.161	3.331	8.64E-04	1.30E-02
Dmel_CR45121	50.125	1.516	0.455	3.331	8.64E-04	1.30E-02
Dmel_CG12344	38.021	-1.307	0.392	-3.330	8.69E-04	1.31E-02
Dmel_CG31666	208.145	-0.975	0.293	-3.327	8.76E-04	1.31E-02
Dmel_CG15362	501.697	-0.726	0.218	-3.327	8.79E-04	1.32E-02
Dmel_CG6282	29.876	-1.687	0.508	-3.322	8.93E-04	1.34E-02
Dmel_CG18155	80.770	1.100	0.331	3.321	8.97E-04	1.34E-02
Dmel_CG18550	29.990	-1.155	0.348	-3.321	8.97E-04	1.34E-02
Dmel_CG14644	19.112	-1.835	0.553	-3.319	9.03E-04	1.34E-02
Dmel_CG33978	236.502	-1.110	0.335	-3.316	9.12E-04	1.36E-02
Dmel_CG6953	7.399	-2.783	0.841	-3.308	9.38E-04	1.39E-02
Dmel_CG9155	396.786	-0.873	0.264	-3.308	9.39E-04	1.39E-02
Dmel_CR44841	147.190	2.036	0.616	3.304	9.53E-04	1.41E-02
Dmel_CR43278	17.357	1.816	0.550	3.303	9.56E-04	1.41E-02
Dmel_CG16727	9.877	-2.937	0.890	-3.302	9.59E-04	1.42E-02
Dmel_CG43772	8.841	2.289	0.694	3.299	9.70E-04	1.43E-02
Dmel_CG30058	14.018	1.569	0.476	3.295	9.85E-04	1.45E-02
Dmel_CG9503	341.519	-0.705	0.214	-3.294	9.86E-04	1.45E-02
Dmel_CG8453	48.480	-2.074	0.630	-3.292	9.95E-04	1.46E-02
Dmel_CG6202	3774.453	0.413	0.125	3.290	1.00E-03	1.47E-02
Dmel_CG9203	680.819	0.559	0.170	3.284	1.02E-03	1.50E-02
Dmel_CG10424	667.728	-0.482	0.147	-3.282	1.03E-03	1.51E-02
Dmel_CR34535	12.981	-2.039	0.621	-3.283	1.03E-03	1.51E-02
Dmel_CR45335	4.273	-3.693	1.126	-3.280	1.04E-03	1.51E-02
Dmel_CR46485	143.767	0.819	0.250	3.280	1.04E-03	1.51E-02

Dmel_CG5381	714.190	-0.894	0.273	-3.277	1.05E-03	1.53E-02
Dmel_CG42292	4.012	-3.931	1.201	-3.274	1.06E-03	1.54E-02
Dmel_CR45225	15.865	-1.435	0.438	-3.274	1.06E-03	1.54E-02
Dmel_CG11500	360.013	-0.970	0.297	-3.270	1.07E-03	1.55E-02
Dmel_CG32639	35.139	1.443	0.441	3.271	1.07E-03	1.55E-02
Dmel_CG30428	377.270	0.990	0.303	3.269	1.08E-03	1.56E-02
Dmel_CG5905	30.547	-1.520	0.465	-3.268	1.08E-03	1.57E-02
Dmel_CR34570	18.906	1.670	0.511	3.266	1.09E-03	1.57E-02
Dmel_CG12484	15.035	1.633	0.500	3.264	1.10E-03	1.58E-02
Dmel_CG9509	63.869	-1.305	0.400	-3.264	1.10E-03	1.58E-02
Dmel_CG12493	49.069	-1.106	0.339	-3.262	1.11E-03	1.59E-02
Dmel_CR46048	204.191	1.157	0.355	3.262	1.11E-03	1.59E-02
Dmel_CG12242	24.371	-1.881	0.577	-3.258	1.12E-03	1.61E-02
Dmel_CR33987	5.856	-3.176	0.976	-3.253	1.14E-03	1.63E-02
Dmel_CG11254	2543.570	0.494	0.152	3.253	1.14E-03	1.63E-02
Dmel_CR45052	9.726	1.810	0.557	3.252	1.15E-03	1.63E-02
Dmel_CG3180	5430.417	0.472	0.145	3.251	1.15E-03	1.64E-02
Dmel_CG13162	4958.261	0.650	0.200	3.248	1.16E-03	1.65E-02
Dmel_CG12355	88.195	1.418	0.438	3.239	1.20E-03	1.70E-02
Dmel_CG31661	694.811	1.283	0.396	3.239	1.20E-03	1.70E-02
Dmel_CG3533	709.897	0.595	0.184	3.240	1.20E-03	1.70E-02
Dmel_CG31760	6.061	-2.663	0.823	-3.235	1.22E-03	1.72E-02
Dmel_CG3526	23.555	1.393	0.431	3.234	1.22E-03	1.72E-02
Dmel_CG30345	500.338	-0.774	0.240	-3.227	1.25E-03	1.76E-02
Dmel_CG11937	394.841	1.254	0.389	3.226	1.26E-03	1.77E-02
Dmel_CG12692	6.274	-2.248	0.697	-3.226	1.26E-03	1.77E-02
Dmel_CG7938	124.712	-1.225	0.380	-3.225	1.26E-03	1.77E-02
Dmel_CG9281	6711.799	0.780	0.242	3.223	1.27E-03	1.78E-02
Dmel_CG14620	142.968	0.811	0.252	3.220	1.28E-03	1.79E-02
Dmel_CG43161	11.588	-1.841	0.572	-3.220	1.28E-03	1.79E-02
Dmel_CG12295	35.781	1.357	0.422	3.217	1.30E-03	1.81E-02
Dmel_CG32572	6.465	2.419	0.752	3.215	1.30E-03	1.82E-02

Dmel_CG3964	83.092	-1.019	0.317	-3.215	1.30E-03	1.82E-02
Dmel_CG8532	1616.996	-0.506	0.157	-3.216	1.30E-03	1.82E-02
Dmel_CG1618	539.997	0.960	0.299	3.214	1.31E-03	1.82E-02
Dmel_CG7672	11.702	1.904	0.593	3.211	1.32E-03	1.84E-02
Dmel_CG14419	12.691	1.942	0.605	3.209	1.33E-03	1.85E-02
Dmel_CG3239	15.410	-3.234	1.008	-3.208	1.34E-03	1.85E-02
Dmel_CG4821	225.884	-0.959	0.299	-3.206	1.35E-03	1.86E-02
Dmel_CG12194	17.091	-1.938	0.605	-3.204	1.36E-03	1.88E-02
Dmel_CG3252	14.207	-2.242	0.701	-3.199	1.38E-03	1.90E-02
Dmel_CG10207	23.822	-2.141	0.670	-3.197	1.39E-03	1.91E-02
Dmel_CG6449	103.030	-0.934	0.292	-3.197	1.39E-03	1.91E-02
Dmel_CG7221	353.424	0.505	0.158	3.192	1.41E-03	1.94E-02
Dmel_CR43866	19.789	1.269	0.397	3.192	1.41E-03	1.94E-02
Dmel_CR46451	4.256	-2.936	0.920	-3.192	1.42E-03	1.94E-02
Dmel_CG10630	13.277	-2.498	0.783	-3.191	1.42E-03	1.94E-02
Dmel_CG43968	34.300	-1.639	0.514	-3.190	1.42E-03	1.94E-02
Dmel_CG42326	4.987	-2.601	0.816	-3.189	1.43E-03	1.95E-02
Dmel_CG7083	1256.892	0.491	0.154	3.187	1.44E-03	1.96E-02
Dmel_CG11951	16.133	-1.770	0.556	-3.181	1.47E-03	2.00E-02
Dmel_CG33988	8.586	-1.871	0.588	-3.181	1.47E-03	2.00E-02
Dmel_CG42732	1224.002	0.612	0.193	3.180	1.47E-03	2.00E-02
Dmel_CG11255	998.369	-0.899	0.283	-3.179	1.48E-03	2.01E-02
Dmel_CG3918	1166.254	-0.917	0.289	-3.177	1.49E-03	2.02E-02
Dmel_CG10444	1982.603	-0.834	0.263	-3.176	1.49E-03	2.02E-02
Dmel_CG11387	867.178	0.889	0.280	3.172	1.51E-03	2.05E-02
Dmel_CG2857	23.133	-1.681	0.530	-3.172	1.51E-03	2.05E-02
Dmel_CG4496	194.538	0.797	0.252	3.166	1.55E-03	2.08E-02
Dmel_CG4715	932.204	1.110	0.351	3.165	1.55E-03	2.08E-02
Dmel_CG7054	954.374	0.660	0.209	3.165	1.55E-03	2.08E-02
Dmel_CR44987	153.397	0.870	0.275	3.166	1.55E-03	2.08E-02
Dmel_CG13871	15.439	2.691	0.851	3.163	1.56E-03	2.10E-02
Dmel_CG7497	146.135	-0.872	0.276	-3.162	1.57E-03	2.10E-02

Dmel_CG3176	41.451	-1.512	0.478	-3.161	1.57E-03	2.10E-02
Dmel_CR45471	11.922	1.836	0.581	3.161	1.57E-03	2.10E-02
Dmel_CG15102	2050.109	-0.525	0.166	-3.160	1.58E-03	2.11E-02
Dmel_CG44880	229.239	0.758	0.240	3.160	1.58E-03	2.11E-02
Dmel_CG30106	16.974	-2.048	0.649	-3.154	1.61E-03	2.14E-02
Dmel_CG5059	2186.489	0.800	0.254	3.152	1.62E-03	2.16E-02
Dmel_CG32373	792.364	1.121	0.356	3.148	1.65E-03	2.18E-02
Dmel_CR46029	29.522	-1.437	0.457	-3.148	1.64E-03	2.18E-02
Dmel_CG33181	2963.425	0.751	0.239	3.146	1.65E-03	2.19E-02
Dmel_CG5041	570.781	0.613	0.195	3.144	1.67E-03	2.20E-02
Dmel_CG11892	208.524	-2.690	0.856	-3.142	1.68E-03	2.22E-02
Dmel_CG8864	7.182	-3.023	0.962	-3.142	1.68E-03	2.22E-02
Dmel_CG5391	5.261	2.931	0.933	3.140	1.69E-03	2.23E-02
Dmel_CG33126	197.114	-1.147	0.366	-3.137	1.71E-03	2.24E-02
Dmel_CG9460	9.877	-1.909	0.608	-3.137	1.70E-03	2.24E-02
Dmel_CG33140	6.012	-2.506	0.800	-3.134	1.73E-03	2.27E-02
Dmel_CG5835	43.348	-0.978	0.312	-3.133	1.73E-03	2.27E-02
Dmel_CG3254	25.889	-1.471	0.470	-3.132	1.74E-03	2.28E-02
Dmel_CG34161	8.362	-2.108	0.674	-3.129	1.75E-03	2.29E-02
Dmel_CR34335	36494.636	-1.023	0.327	-3.127	1.76E-03	2.31E-02
Dmel_CG18870	2820.357	0.447	0.143	3.126	1.77E-03	2.32E-02
Dmel_CG10702	1150.706	0.814	0.261	3.124	1.78E-03	2.33E-02
Dmel_CG10734	120.828	0.968	0.310	3.121	1.80E-03	2.34E-02
Dmel_CG13784	2273.112	0.439	0.141	3.120	1.81E-03	2.35E-02
Dmel_CR32957	2001.447	0.688	0.221	3.120	1.81E-03	2.35E-02
Dmel_CG6503	26.425	-3.794	1.216	-3.119	1.81E-03	2.35E-02
Dmel_CG30334	16.024	1.724	0.553	3.119	1.82E-03	2.35E-02
Dmel_CG34331	7.516	-2.857	0.917	-3.114	1.85E-03	2.39E-02
Dmel_CG6584	2008.800	0.434	0.139	3.113	1.85E-03	2.40E-02
Dmel_CG4733	177.034	0.838	0.269	3.111	1.86E-03	2.41E-02
Dmel_CG9456	35.892	-1.475	0.475	-3.108	1.89E-03	2.43E-02
Dmel_CG9650	454.888	-0.791	0.255	-3.106	1.90E-03	2.45E-02

Dmel_CG15309	1390.190	0.451	0.145	3.105	1.90E-03	2.45E-02
Dmel_CR43962	8.553	-2.440	0.786	-3.103	1.92E-03	2.47E-02
Dmel_CG2060	1551.905	0.661	0.213	3.102	1.92E-03	2.47E-02
Dmel_CG41284	85.265	1.643	0.531	3.096	1.96E-03	2.51E-02
Dmel_CG8380	8.756	1.965	0.635	3.095	1.97E-03	2.52E-02
Dmel_CG31365	1258.248	0.778	0.251	3.094	1.98E-03	2.53E-02
Dmel_CG5612	169.169	-0.991	0.321	-3.091	2.00E-03	2.55E-02
Dmel_CG8083	13.191	-1.741	0.563	-3.091	2.00E-03	2.55E-02
Dmel_CG3705	1508.729	-0.635	0.205	-3.090	2.00E-03	2.55E-02
Dmel_CG13282	99.633	-1.054	0.341	-3.088	2.01E-03	2.56E-02
Dmel_CG16799	9.929	-2.275	0.737	-3.087	2.02E-03	2.57E-02
Dmel_CG12346	351.844	-0.783	0.254	-3.084	2.04E-03	2.59E-02
Dmel_CG10005	59.957	-1.055	0.342	-3.084	2.04E-03	2.59E-02
Dmel_CG12002	217.218	-1.329	0.432	-3.079	2.08E-03	2.63E-02
Dmel_CG12684	9.256	2.071	0.673	3.079	2.08E-03	2.63E-02
Dmel_CG17795	17.109	-1.962	0.638	-3.077	2.09E-03	2.64E-02
Dmel_CG17217	8.090	-2.152	0.700	-3.075	2.10E-03	2.65E-02
Dmel_CG6071	45.004	1.312	0.427	3.076	2.10E-03	2.65E-02
Dmel_CG18548	12.434	-1.665	0.542	-3.074	2.11E-03	2.66E-02
Dmel_CG7595	4521.269	0.400	0.130	3.072	2.13E-03	2.67E-02
Dmel_CG11634	4.279	-3.755	1.222	-3.071	2.13E-03	2.68E-02
Dmel_CG9207	300.491	-0.757	0.247	-3.069	2.15E-03	2.69E-02
Dmel_CR33686	13262.350	0.888	0.289	3.068	2.15E-03	2.70E-02
Dmel_CG17716	19.533	-1.772	0.578	-3.066	2.17E-03	2.71E-02
Dmel_CG40298	76.791	1.427	0.465	3.065	2.17E-03	2.72E-02
Dmel_CG17970	165.747	0.974	0.318	3.063	2.19E-03	2.74E-02
Dmel_CG7041	1004.052	0.604	0.197	3.063	2.19E-03	2.74E-02
Dmel_CG8339	1557.479	0.337	0.110	3.063	2.19E-03	2.74E-02
Dmel_CG32506	9.534	-1.944	0.635	-3.062	2.20E-03	2.74E-02
Dmel_CG9772	2989.847	0.486	0.159	3.060	2.21E-03	2.75E-02
Dmel_CG30047	10.082	-2.525	0.827	-3.055	2.25E-03	2.80E-02
Dmel_CG4257	9979.136	0.364	0.119	3.054	2.26E-03	2.80E-02

Dmel_CG5778	6.358	-1.972	0.646	-3.052	2.27E-03	2.81E-02
Dmel_CG6677	880.216	0.618	0.203	3.052	2.27E-03	2.81E-02
Dmel_CR45534	7.894	1.872	0.614	3.048	2.30E-03	2.85E-02
Dmel_CG1634	2307.073	-0.690	0.227	-3.047	2.31E-03	2.85E-02
Dmel_CG1806	80.643	0.842	0.276	3.046	2.32E-03	2.86E-02
Dmel_CG15576	10.175	-2.048	0.672	-3.045	2.32E-03	2.86E-02
Dmel_CG33093	9.378	2.029	0.667	3.041	2.36E-03	2.90E-02
Dmel_CG11899	1333.295	0.723	0.238	3.039	2.37E-03	2.92E-02
Dmel_CG34166	13.640	-3.258	1.073	-3.036	2.39E-03	2.94E-02
Dmel_CG6303	18054.014	0.312	0.103	3.035	2.40E-03	2.95E-02
Dmel_CG12120	10.092	-1.932	0.637	-3.034	2.41E-03	2.95E-02
Dmel_CG17524	127.121	-0.831	0.274	-3.033	2.42E-03	2.96E-02
Dmel_CG6575	11723.986	0.711	0.234	3.033	2.42E-03	2.96E-02
Dmel_CG3905	717.826	0.548	0.181	3.031	2.44E-03	2.98E-02
Dmel_CG9677	7820.096	0.424	0.140	3.030	2.45E-03	2.99E-02
Dmel_CG5904	373.382	0.587	0.194	3.028	2.46E-03	2.99E-02
Dmel_CG7913	6017.463	0.432	0.143	3.028	2.46E-03	2.99E-02
Dmel_CG3971	4591.617	0.532	0.176	3.027	2.47E-03	3.01E-02
Dmel_CG13315	109.740	-1.502	0.497	-3.026	2.48E-03	3.01E-02
Dmel_CR44115	21.000	-2.527	0.836	-3.023	2.50E-03	3.04E-02
Dmel_CG31673	855.982	-0.521	0.173	-3.022	2.51E-03	3.04E-02
Dmel_CR43018	20.119	-5.429	1.797	-3.022	2.51E-03	3.04E-02
Dmel_CG32656	29.905	-1.495	0.495	-3.021	2.52E-03	3.05E-02
Dmel_CG13663	409.022	-0.692	0.229	-3.019	2.53E-03	3.06E-02
Dmel_CG15040	6.619	-2.235	0.741	-3.018	2.54E-03	3.07E-02
Dmel_CG14275	101.798	0.985	0.326	3.017	2.55E-03	3.07E-02
Dmel_CR33674	23.624	1.375	0.456	3.016	2.56E-03	3.08E-02
Dmel_CG30195	12.984	-1.876	0.623	-3.014	2.58E-03	3.09E-02
Dmel_CG6511	1515.921	0.437	0.145	3.014	2.57E-03	3.09E-02
Dmel_CG8808	3822.949	0.618	0.205	3.012	2.59E-03	3.11E-02
Dmel_CG11064	2076.511	-1.666	0.554	-3.007	2.64E-03	3.16E-02
Dmel_CG6495	14.977	2.389	0.795	3.004	2.67E-03	3.19E-02

Dmel_CG33960	90.640	1.186	0.395	3.002	2.69E-03	3.21E-02
Dmel_CG42249	22.299	-1.121	0.374	-3.000	2.70E-03	3.22E-02
Dmel_CG9242	7363.625	0.548	0.183	3.000	2.70E-03	3.22E-02
Dmel_CG31898	1376.311	0.923	0.308	2.996	2.74E-03	3.26E-02
Dmel_CR42491	542.160	-0.920	0.307	-2.995	2.74E-03	3.26E-02
Dmel_CG2102	31.808	-1.577	0.527	-2.995	2.75E-03	3.26E-02
Dmel_CG31445	45.239	-1.471	0.491	-2.993	2.76E-03	3.27E-02
Dmel_CG43295	68.057	-0.972	0.325	-2.993	2.76E-03	3.27E-02
Dmel_CG5337	106.836	-0.633	0.212	-2.993	2.76E-03	3.27E-02
Dmel_CG10466	102.353	-1.024	0.342	-2.992	2.77E-03	3.28E-02
Dmel_CG11783	659.129	-0.599	0.200	-2.989	2.80E-03	3.30E-02
Dmel_CG3504	37.047	-1.060	0.355	-2.989	2.80E-03	3.30E-02
Dmel_CG4579	7553.916	0.337	0.113	2.989	2.80E-03	3.30E-02
Dmel_CG32146	3320.000	0.565	0.189	2.988	2.81E-03	3.31E-02
Dmel_CG6217	22.325	-1.437	0.481	-2.986	2.83E-03	3.32E-02
Dmel_CG12179	1309.413	0.444	0.149	2.983	2.85E-03	3.35E-02
Dmel_CG34323	38.530	-1.299	0.436	-2.982	2.86E-03	3.36E-02
Dmel_CG33926	47.226	-1.752	0.588	-2.981	2.87E-03	3.37E-02
Dmel_CG7447	113.824	1.091	0.366	2.979	2.89E-03	3.39E-02
Dmel_CR46083	34.947	1.067	0.360	2.967	3.01E-03	3.52E-02
Dmel_CG2706	50.078	-1.306	0.441	-2.966	3.02E-03	3.53E-02
Dmel_CG9098	194.284	0.586	0.198	2.963	3.05E-03	3.56E-02
Dmel_CG12708	73.374	1.280	0.432	2.961	3.07E-03	3.58E-02
Dmel_CG13827	209.725	0.751	0.254	2.958	3.09E-03	3.60E-02
Dmel_CG2736	76.651	-0.862	0.291	-2.959	3.09E-03	3.60E-02
Dmel_CR45179	43.006	1.321	0.447	2.958	3.10E-03	3.60E-02
Dmel_CG1950	10.505	-2.311	0.783	-2.950	3.18E-03	3.69E-02
Dmel_CG9611	723.808	0.458	0.155	2.949	3.19E-03	3.70E-02
Dmel_CR33753	103.198	1.049	0.356	2.947	3.21E-03	3.71E-02
Dmel_CG15848	101.067	1.135	0.386	2.944	3.24E-03	3.75E-02
Dmel_CG2194	15.037	-1.685	0.573	-2.939	3.29E-03	3.80E-02
Dmel_CR43493	219.103	0.871	0.296	2.940	3.28E-03	3.80E-02

Dmel_CG18372	32.607	-2.602	0.885	-2.938	3.30E-03	3.81E-02
Dmel_CG31764	398.620	-0.674	0.230	-2.938	3.31E-03	3.81E-02
Dmel_CG34392	202.441	1.223	0.417	2.935	3.34E-03	3.85E-02
Dmel_CG42600	8452.538	0.533	0.182	2.934	3.35E-03	3.85E-02
Dmel_CG42739	692.104	0.780	0.266	2.933	3.36E-03	3.86E-02
Dmel_CG46511	10.848	2.683	0.916	2.930	3.39E-03	3.89E-02
Dmel_CG8930	31.973	1.092	0.373	2.929	3.40E-03	3.90E-02
Dmel_CG11152	73.962	1.292	0.441	2.928	3.41E-03	3.91E-02
Dmel_CG14142	22.763	1.082	0.370	2.927	3.42E-03	3.91E-02
Dmel_CG10901	69851.455	0.493	0.169	2.927	3.43E-03	3.92E-02
Dmel_CG9610	35.323	-1.337	0.457	-2.926	3.43E-03	3.92E-02
Dmel_CG1112	34.251	-1.273	0.435	-2.924	3.45E-03	3.93E-02
Dmel_CG15035	33.968	1.521	0.520	2.925	3.45E-03	3.93E-02
Dmel_CG9414	984.747	-0.576	0.197	-2.924	3.45E-03	3.93E-02
Dmel_CG3757	66.606	-1.071	0.366	-2.924	3.46E-03	3.93E-02
Dmel_CG13309	45.049	-5.610	1.920	-2.922	3.47E-03	3.94E-02
Dmel_CG3694	9.047	2.078	0.711	2.922	3.48E-03	3.94E-02
Dmel_CG7660	20442.794	0.369	0.126	2.922	3.48E-03	3.94E-02
Dmel_CG42626	9.841	-1.383	0.474	-2.921	3.49E-03	3.95E-02
Dmel_CG43770	12411.707	0.490	0.168	2.921	3.49E-03	3.95E-02
Dmel_CG7910	43.394	1.649	0.565	2.919	3.51E-03	3.97E-02
Dmel_CG11852	53.225	-1.131	0.388	-2.918	3.52E-03	3.97E-02
Dmel_CG8014	7309.156	0.345	0.118	2.918	3.52E-03	3.97E-02
Dmel_CG8318	6370.629	0.435	0.149	2.915	3.55E-03	4.00E-02
Dmel_CG30339	24.402	-1.351	0.464	-2.912	3.59E-03	4.03E-02
Dmel_CG13113	571.943	1.134	0.390	2.912	3.59E-03	4.04E-02
Dmel_CG2849	5829.301	-0.601	0.207	-2.909	3.62E-03	4.06E-02
Dmel_CG40494	5617.941	0.382	0.131	2.909	3.62E-03	4.06E-02
Dmel_CG2699	9132.501	0.534	0.184	2.908	3.64E-03	4.07E-02
Dmel_CG43370	11.643	-1.723	0.593	-2.908	3.64E-03	4.07E-02
Dmel_CG42356	20.310	1.454	0.500	2.906	3.66E-03	4.09E-02
Dmel_CG3004	1347.285	0.378	0.130	2.904	3.69E-03	4.11E-02

Dmel_CG17294	289.599	-0.625	0.215	-2.903	3.69E-03	4.11E-02
Dmel_CG42357	20.533	1.583	0.545	2.903	3.69E-03	4.11E-02
Dmel_CG30156	45.744	0.920	0.317	2.900	3.74E-03	4.15E-02
Dmel_CR32900	11.168	1.445	0.498	2.900	3.73E-03	4.15E-02
Dmel_CG12605	8.184	-2.030	0.701	-2.894	3.80E-03	4.22E-02
Dmel_CG45088	63.539	1.460	0.505	2.893	3.81E-03	4.22E-02
Dmel_CG9256	405.857	-0.703	0.243	-2.893	3.81E-03	4.22E-02
Dmel_CR46094	67.098	0.875	0.302	2.894	3.81E-03	4.22E-02
Dmel_CR46090	25.206	0.995	0.344	2.893	3.82E-03	4.22E-02
Dmel_CG14341	124.607	-1.008	0.349	-2.892	3.83E-03	4.23E-02
Dmel_CG31038	332.270	0.559	0.193	2.892	3.83E-03	4.23E-02
Dmel_CG34433	25.355	1.353	0.468	2.892	3.83E-03	4.23E-02
Dmel_CG10777	10216.604	0.507	0.175	2.889	3.86E-03	4.25E-02
Dmel_CG13888	26.473	-0.993	0.344	-2.889	3.86E-03	4.25E-02
Dmel_CG4559	243.698	-0.610	0.211	-2.889	3.86E-03	4.25E-02
Dmel_CG3259	25.044	-1.221	0.423	-2.888	3.87E-03	4.25E-02
Dmel_CG15525	215.123	-0.719	0.249	-2.885	3.91E-03	4.29E-02
Dmel_CG32077	13.884	1.639	0.568	2.885	3.92E-03	4.29E-02
Dmel_CG17129	2764.223	0.763	0.264	2.884	3.93E-03	4.30E-02
Dmel_CG44195	214.639	0.979	0.339	2.884	3.93E-03	4.30E-02
Dmel_CG7607	91.260	0.863	0.299	2.883	3.93E-03	4.30E-02
Dmel_CG4170	1895.737	-0.667	0.231	-2.882	3.95E-03	4.30E-02
Dmel_CG8665	64.322	-0.946	0.328	-2.882	3.95E-03	4.30E-02
Dmel_CG17060	4727.730	0.355	0.123	2.880	3.97E-03	4.32E-02
Dmel_CG9492	5.030	-2.490	0.864	-2.880	3.97E-03	4.32E-02
Dmel_CG13317	26.761	1.699	0.590	2.879	3.99E-03	4.33E-02
Dmel_CG15739	27.147	-1.222	0.424	-2.879	3.99E-03	4.34E-02
Dmel_CG8651	11703.032	0.342	0.119	2.876	4.03E-03	4.38E-02
Dmel_CG6330	698.336	-0.683	0.238	-2.873	4.06E-03	4.40E-02
Dmel_CG6542	4210.347	0.424	0.148	2.872	4.08E-03	4.42E-02
Dmel_CG31743	245.562	0.807	0.281	2.871	4.09E-03	4.42E-02
Dmel_CG3424	18475.822	0.403	0.141	2.871	4.10E-03	4.42E-02

Dmel_CG17717	17.339	-1.491	0.520	-2.866	4.15E-03	4.48E-02
Dmel_CG44325	724.990	-0.660	0.230	-2.863	4.19E-03	4.52E-02
Dmel_CG7635	8.520	-2.027	0.708	-2.863	4.20E-03	4.52E-02
Dmel_CR45047	18.644	1.176	0.411	2.863	4.20E-03	4.52E-02
Dmel_CG11000	88.807	1.094	0.382	2.860	4.24E-03	4.56E-02
Dmel_CG10006	27.615	-1.283	0.449	-2.859	4.25E-03	4.56E-02
Dmel_CG1774	146.285	-0.977	0.342	-2.857	4.28E-03	4.58E-02
Dmel_CG31992	20913.729	0.405	0.142	2.856	4.29E-03	4.58E-02
Dmel_CG4180	1002.902	-0.407	0.143	-2.856	4.29E-03	4.58E-02
Dmel_CG7134	1948.993	0.544	0.190	2.857	4.28E-03	4.58E-02
Dmel_CG9682	8.008	-1.986	0.695	-2.855	4.30E-03	4.60E-02
Dmel_CG3544	5.634	-2.084	0.730	-2.854	4.31E-03	4.60E-02
Dmel_CG8827	140.828	-1.011	0.354	-2.854	4.31E-03	4.60E-02
Dmel_CG30343	277.129	-0.634	0.222	-2.853	4.34E-03	4.61E-02
Dmel_CG46520	3.070	-5.281	1.851	-2.853	4.34E-03	4.61E-02
Dmel_CG46522	3.070	-5.281	1.851	-2.853	4.34E-03	4.61E-02
Dmel_CG10146	15.302	-2.344	0.823	-2.849	4.39E-03	4.66E-02
Dmel_CG42854	30.574	-1.603	0.563	-2.849	4.39E-03	4.66E-02
Dmel_CG12423	221.162	-0.929	0.326	-2.845	4.44E-03	4.71E-02
Dmel_CG6490	2609.600	0.762	0.268	2.845	4.44E-03	4.71E-02
Dmel_CG8805	2531.220	0.438	0.154	2.844	4.45E-03	4.71E-02
Dmel_CG9372	9.420	-1.894	0.667	-2.840	4.52E-03	4.77E-02
Dmel_CR34607	29.650	1.410	0.497	2.838	4.53E-03	4.79E-02
Dmel_CG14193	12.461	-1.601	0.565	-2.837	4.56E-03	4.80E-02
Dmel_CG18473	60.517	1.180	0.416	2.836	4.56E-03	4.80E-02
Dmel_CG3039	3401.084	0.700	0.247	2.837	4.56E-03	4.80E-02
Dmel_CG1772	8275.607	0.661	0.233	2.833	4.61E-03	4.84E-02
Dmel_CG8205	2676.441	0.546	0.193	2.832	4.63E-03	4.86E-02
Dmel_CG6207	2745.065	0.598	0.212	2.825	4.72E-03	4.96E-02
Dmel_CG8316	213.397	-0.793	0.281	-2.825	4.73E-03	4.96E-02
Dmel_CG10479	75.907	1.041	0.369	2.821	4.79E-03	5.02E-02
Dmel_CR45758	6.430	2.623	0.930	2.820	4.81E-03	5.03E-02

Dmel_CG11128	2190.850	0.888	0.315	2.817	4.84E-03	5.06E-02
Dmel_CR46037	8285.229	-1.108	0.393	-2.817	4.84E-03	5.06E-02
Dmel_CG43079	631.857	-1.001	0.355	-2.816	4.86E-03	5.07E-02
Dmel_CG7460	93.172	1.036	0.368	2.814	4.89E-03	5.10E-02
Dmel_CG34220	261.387	1.093	0.388	2.813	4.91E-03	5.11E-02
Dmel_CG14695	5.018	-2.908	1.034	-2.812	4.93E-03	5.13E-02
Dmel_CG34455	1000.243	0.458	0.163	2.808	4.98E-03	5.18E-02
Dmel_CG15822	271.011	0.876	0.312	2.806	5.02E-03	5.21E-02
Dmel_CG14032	170.072	1.031	0.368	2.805	5.03E-03	5.21E-02
Dmel_CG2902	12.707	-1.599	0.570	-2.802	5.08E-03	5.26E-02
Dmel_CG3290	41.169	-5.759	2.055	-2.802	5.08E-03	5.26E-02
Dmel_CG10553	15.343	-2.517	0.898	-2.801	5.09E-03	5.26E-02
Dmel_CG7470	8.831	-2.032	0.725	-2.801	5.10E-03	5.27E-02
Dmel_CG32086	4.755	-2.790	0.998	-2.797	5.16E-03	5.33E-02
Dmel_CG7272	93.577	-0.653	0.234	-2.797	5.16E-03	5.33E-02
Dmel_CG3241	198.254	0.608	0.217	2.795	5.19E-03	5.35E-02
Dmel_CG11310	8.366	2.046	0.733	2.792	5.25E-03	5.40E-02
Dmel_CR45036	19.168	-1.346	0.483	-2.790	5.27E-03	5.42E-02
Dmel_CG4006	3810.911	0.486	0.174	2.789	5.29E-03	5.43E-02
Dmel_CG7363	14.727	-1.578	0.566	-2.789	5.29E-03	5.43E-02
Dmel_CG14064	16.145	1.903	0.683	2.788	5.31E-03	5.44E-02
Dmel_CG1307	973.268	-0.811	0.291	-2.787	5.32E-03	5.44E-02
Dmel_CG2767	1424.389	0.545	0.195	2.787	5.32E-03	5.44E-02

table S17. *D. melanogaster* genes Wald Test significant results for ~Genotype vs ~Genotype+Infection+Genotype*Infection
