# Supplementary material for: Wolbachia endosymbionts manipulate the self-renewal and differentiation of germline stem cells to reinforce fertility of their fruit fly host
Source: PLoS Biol. 2023 Oct 24;21(10):e3002335. doi: 10.1371/journal.pbio.3002335 (PMC10597519; doi:10.1371/journal.pbio.3002335)
Supplement: S18 Table — (PDF) [file pbio.3002335.s033.pdf]

gene_id	baseMean	log2FoldChange	lfcSE	stat	pvalue	padj
Dmel_CG43079	631.857	-1.956	0.356	-5.494	3.92E-08	2.56E-04
Dmel_CG9871	50.560	-3.292	0.610	-5.399	6.69E-08	2.56E-04
Dmel_CG7002	154.856	-2.425	0.450	-5.387	7.17E-08	2.56E-04
Dmel_CG13772	15.539	-3.435	0.646	-5.318	1.05E-07	2.80E-04
Dmel_CG10593	2009.122	2.010	0.385	5.220	1.79E-07	3.83E-04
Dmel_CG15599	55.457	-2.867	0.557	-5.151	2.59E-07	4.61E-04
Dmel_CG6533	2736.531	4.123	0.808	5.101	3.38E-07	5.16E-04
Dmel_CG9073	36.796	-2.448	0.487	-5.031	4.89E-07	6.54E-04
Dmel_CG6524	6268.890	3.938	0.791	4.976	6.48E-07	7.54E-04
Dmel_CR46481	1090.588	-1.267	0.256	-4.947	7.54E-07	7.54E-04
Dmel_CG2187	500.405	1.275	0.258	4.937	7.95E-07	7.54E-04
Dmel_CG11941	16.019	-3.213	0.653	-4.924	8.46E-07	7.54E-04
Dmel_CG8663	114.131	-1.984	0.409	-4.856	1.19E-06	9.83E-04
Dmel_CG31973	444.390	-1.252	0.260	-4.814	1.48E-06	1.13E-03
Dmel_CG42711	9.934	-4.252	0.888	-4.790	1.66E-06	1.19E-03
Dmel_CG10287	102.780	-1.574	0.330	-4.776	1.79E-06	1.19E-03
Dmel_CR32368	12.082	-3.326	0.708	-4.699	2.61E-06	1.64E-03
Dmel_CG30046	65.427	1.508	0.322	4.685	2.80E-06	1.65E-03
Dmel_CG8942	20.884	-3.404	0.728	-4.676	2.93E-06	1.65E-03
Dmel_CG34323	38.530	-2.054	0.441	-4.661	3.14E-06	1.68E-03
Dmel_CG5644	28.092	2.236	0.486	4.598	4.26E-06	2.17E-03
Dmel_CG4373	307.928	-2.564	0.564	-4.547	5.44E-06	2.58E-03
Dmel_CG13084	2952.416	3.172	0.700	4.532	5.83E-06	2.58E-03
Dmel_CG4783	18.606	-2.194	0.484	-4.531	5.87E-06	2.58E-03
Dmel_CG3157	1113.612	0.878	0.194	4.524	6.08E-06	2.58E-03
Dmel_CG1091	2685.582	0.732	0.162	4.517	6.26E-06	2.58E-03
Dmel_CG6517	5048.988	3.842	0.857	4.483	7.35E-06	2.91E-03
Dmel_CG8388	689.979	0.544	0.121	4.474	7.66E-06	2.93E-03
Dmel_CG3635	29.922	-2.392	0.543	-4.406	1.05E-05	3.89E-03
Dmel_CG32640	167.387	-1.214	0.278	-4.373	1.22E-05	4.36E-03

Dmel_CG9663	323.692	1.203	0.276	4.355	1.33E-05	4.59E-03
Dmel_CR46350	65.211	-1.765	0.407	-4.332	1.48E-05	4.90E-03
Dmel_CG5937	33.106	-2.091	0.483	-4.327	1.51E-05	4.90E-03
Dmel_CG3625	103.288	-0.979	0.228	-4.284	1.84E-05	5.60E-03
Dmel_CG32082	143.362	-1.278	0.299	-4.279	1.87E-05	5.60E-03
Dmel_CG8805	2531.220	0.658	0.154	4.278	1.89E-05	5.60E-03
Dmel_CG6542	4210.347	0.628	0.148	4.254	2.10E-05	6.06E-03
Dmel_CG34325	218.050	-1.225	0.289	-4.241	2.23E-05	6.16E-03
Dmel_CG7050	174.496	-1.607	0.379	-4.239	2.25E-05	6.16E-03
Dmel_CG7676	35.784	-1.999	0.474	-4.219	2.45E-05	6.28E-03
Dmel_CG42854	30.574	-2.400	0.569	-4.218	2.47E-05	6.28E-03
Dmel_CG13000	15.067	-2.359	0.560	-4.214	2.51E-05	6.28E-03
Dmel_CG10726	2404.031	0.799	0.190	4.212	2.53E-05	6.28E-03
Dmel_CG6178	2685.460	0.496	0.119	4.180	2.91E-05	6.96E-03
Dmel_CG14193	12.461	-2.423	0.580	-4.179	2.93E-05	6.96E-03
Dmel_CG43749	68.898	-2.003	0.484	-4.138	3.50E-05	8.10E-03
Dmel_CG2191	17.249	-2.812	0.680	-4.134	3.56E-05	8.10E-03
Dmel_CG8262	752.433	1.089	0.265	4.107	4.00E-05	8.92E-03
Dmel_CG7178	1358.215	-1.725	0.421	-4.100	4.14E-05	9.03E-03
Dmel_CG3297	3125.993	0.709	0.173	4.092	4.27E-05	9.14E-03
Dmel_CG5252	4020.919	0.503	0.124	4.074	4.63E-05	9.53E-03
Dmel_CG14808	573.015	-1.527	0.375	-4.070	4.69E-05	9.53E-03
Dmel_CG6006	523.400	-1.228	0.302	-4.065	4.80E-05	9.53E-03
Dmel_CG7660	20442.794	0.514	0.126	4.065	4.81E-05	9.53E-03
Dmel_CG16995	24.742	-1.770	0.436	-4.056	5.00E-05	9.72E-03
Dmel_CG2275	1215.191	0.896	0.222	4.036	5.43E-05	1.04E-02
Dmel_CG18870	2820.357	0.576	0.143	4.030	5.57E-05	1.05E-02
Dmel_CG32944	92.791	-1.246	0.310	-4.016	5.91E-05	1.09E-02
Dmel_CG3879	12.492	-2.318	0.578	-4.008	6.12E-05	1.09E-02
Dmel_CG4476	2247.740	1.039	0.259	4.008	6.12E-05	1.09E-02
Dmel_CG12582	1823.184	0.624	0.156	4.005	6.20E-05	1.09E-02
Dmel_CG15814	1716.320	0.639	0.160	3.995	6.47E-05	1.11E-02

Dmel_CG15173	91.496	-1.131	0.284	-3.985	6.73E-05	1.11E-02
Dmel_CG9772	2989.847	0.632	0.159	3.980	6.90E-05	1.11E-02
Dmel_CG18547	97.360	-1.483	0.373	-3.980	6.90E-05	1.11E-02
Dmel_CG6698	17.300	-3.148	0.792	-3.976	7.00E-05	1.11E-02
Dmel_CG18619	37.742	-2.011	0.506	-3.974	7.06E-05	1.11E-02
Dmel_CG12002	217.218	-1.718	0.432	-3.974	7.08E-05	1.11E-02
Dmel_CG3631	610.954	0.637	0.161	3.967	7.29E-05	1.13E-02
Dmel_CG9650	454.888	-1.001	0.255	-3.933	8.40E-05	1.27E-02
Dmel_CG9411	11.974	-2.846	0.724	-3.932	8.44E-05	1.27E-02
Dmel_CG30101	46.664	2.134	0.544	3.923	8.75E-05	1.30E-02
Dmel_CG30489	122.263	-1.002	0.256	-3.916	8.99E-05	1.32E-02
Dmel_CG6477	1858.505	1.056	0.270	3.913	9.12E-05	1.32E-02
Dmel_CG6324	34.299	-1.701	0.436	-3.903	9.51E-05	1.36E-02
Dmel_CG31989	498.990	0.777	0.200	3.893	9.90E-05	1.39E-02
Dmel_CG17078	3064.101	0.927	0.239	3.885	1.02E-04	1.41E-02
Dmel_CG7084	38.686	-2.379	0.613	-3.884	1.03E-04	1.41E-02
Dmel_CG17927	1300.359	-1.575	0.406	-3.877	1.06E-04	1.43E-02
Dmel_CG18568	26.606	1.247	0.322	3.870	1.09E-04	1.44E-02
Dmel_CG14680	22.113	-1.898	0.491	-3.867	1.10E-04	1.44E-02
Dmel_CG8193	9.669	-2.935	0.759	-3.866	1.11E-04	1.44E-02
Dmel_CG10706	358.379	-1.147	0.298	-3.848	1.19E-04	1.54E-02
Dmel_CG42512	8.618	-2.278	0.593	-3.840	1.23E-04	1.57E-02
Dmel_CG43088	13.320	-2.541	0.663	-3.834	1.26E-04	1.57E-02
Dmel_CG8233	3636.327	0.540	0.141	3.834	1.26E-04	1.57E-02
Dmel_CG10638	1166.946	-0.838	0.219	-3.826	1.30E-04	1.60E-02
Dmel_CG17117	134.383	-1.721	0.451	-3.820	1.33E-04	1.62E-02
Dmel_CR45530	16.389	-1.740	0.456	-3.813	1.37E-04	1.65E-02
Dmel_CG13954	10.767	-1.844	0.484	-3.808	1.40E-04	1.66E-02
Dmel_CG16762	7.124	-3.388	0.893	-3.796	1.47E-04	1.71E-02
Dmel_CG14796	11568.819	1.541	0.406	3.795	1.48E-04	1.71E-02
Dmel_CG13928	13.709	-1.897	0.500	-3.793	1.49E-04	1.71E-02
Dmel_CG18522	301.389	-1.485	0.392	-3.787	1.52E-04	1.73E-02

Dmel_CG10521	195.366	-1.332	0.352	-3.784	1.54E-04	1.74E-02
Dmel_CG12283	681.554	-0.997	0.265	-3.759	1.70E-04	1.89E-02
Dmel_CG2893	2410.997	0.588	0.156	3.758	1.71E-04	1.89E-02
Dmel_CG6479	2633.182	0.946	0.252	3.749	1.78E-04	1.92E-02
Dmel_CR43461	15.553	-1.690	0.451	-3.748	1.78E-04	1.92E-02
Dmel_CG33472	105.352	-1.291	0.345	-3.745	1.80E-04	1.93E-02
Dmel_CG12449	143.489	-1.377	0.369	-3.730	1.91E-04	1.98E-02
Dmel_CG9338	181.285	-1.597	0.429	-3.727	1.94E-04	1.98E-02
Dmel_CG6958	2318.760	0.411	0.110	3.727	1.94E-04	1.98E-02
Dmel_CG10553	15.343	-3.397	0.912	-3.726	1.95E-04	1.98E-02
Dmel_CG4099	50.953	-1.505	0.404	-3.726	1.95E-04	1.98E-02
Dmel_CG3812	1006.282	0.566	0.152	3.724	1.96E-04	1.98E-02
Dmel_CG42486	7.794	-1.777	0.478	-3.716	2.02E-04	2.01E-02
Dmel_CG32642	1743.930	2.684	0.723	3.713	2.05E-04	2.01E-02
Dmel_CG42492	197.428	-1.605	0.432	-3.712	2.06E-04	2.01E-02
Dmel_CG15324	366.537	1.612	0.434	3.711	2.06E-04	2.01E-02
Dmel_CG9610	35.323	-1.695	0.458	-3.704	2.12E-04	2.03E-02
Dmel_CG10795	653.543	1.082	0.292	3.703	2.13E-04	2.03E-02
Dmel_CG5338	70.237	-1.831	0.495	-3.699	2.16E-04	2.04E-02
Dmel_CG14615	327.742	0.786	0.213	3.696	2.19E-04	2.04E-02
Dmel_CG6422	4864.193	0.631	0.171	3.692	2.22E-04	2.04E-02
Dmel_CG10550	16.054	-2.006	0.543	-3.692	2.23E-04	2.04E-02
Dmel_CG32364	249.152	-1.255	0.340	-3.690	2.24E-04	2.04E-02
Dmel_CG2857	23.133	-1.953	0.530	-3.688	2.26E-04	2.04E-02
Dmel_CG12375	831.121	0.541	0.147	3.684	2.29E-04	2.04E-02
Dmel_CG11125	345.793	0.825	0.224	3.683	2.30E-04	2.04E-02
Dmel_CG32577	132.606	-1.219	0.331	-3.683	2.30E-04	2.04E-02
Dmel_CR44472	23.272	1.829	0.497	3.679	2.34E-04	2.05E-02
Dmel_CR43144	234.567	-1.219	0.332	-3.674	2.39E-04	2.08E-02
Dmel_CG31901	28.374	-2.490	0.680	-3.664	2.49E-04	2.15E-02
Dmel_CG2246	2660.559	0.526	0.144	3.658	2.54E-04	2.15E-02
Dmel_CG41106	15.890	-1.904	0.521	-3.657	2.55E-04	2.15E-02

Dmel_CG9707	1943.581	0.589	0.161	3.649	2.63E-04	2.15E-02
Dmel_CG32019	11590.256	-1.412	0.387	-3.649	2.64E-04	2.15E-02
Dmel_CG7510	2088.312	0.558	0.153	3.648	2.64E-04	2.15E-02
Dmel_CG5041	570.781	0.710	0.195	3.648	2.64E-04	2.15E-02
Dmel_CG12423	221.162	-1.190	0.326	-3.647	2.65E-04	2.15E-02
Dmel_CG10336	664.150	0.885	0.243	3.646	2.66E-04	2.15E-02
Dmel_CG18039	57.006	-1.759	0.483	-3.644	2.69E-04	2.15E-02
Dmel_CG7564	14794.411	-1.158	0.318	-3.643	2.70E-04	2.15E-02
Dmel_CG42639	16.055	-2.624	0.721	-3.640	2.73E-04	2.16E-02
Dmel_CG15432	193.546	-1.233	0.339	-3.635	2.77E-04	2.18E-02
Dmel_CG45077	281.510	-1.509	0.416	-3.627	2.86E-04	2.23E-02
Dmel_CG13364	597.882	-1.546	0.427	-3.624	2.90E-04	2.23E-02
Dmel_CR34084	11.680	-2.032	0.561	-3.621	2.93E-04	2.23E-02
Dmel_CG46462	29.749	-1.810	0.500	-3.621	2.94E-04	2.23E-02
Dmel_CG10794	23.361	-4.266	1.179	-3.617	2.98E-04	2.23E-02
Dmel_CG33474	26.760	-1.326	0.367	-3.616	2.99E-04	2.23E-02
Dmel_CR45187	109.321	-1.218	0.337	-3.616	2.99E-04	2.23E-02
Dmel_CG32677	64.186	-1.706	0.472	-3.615	3.01E-04	2.23E-02
Dmel_CG15155	16.725	-1.854	0.513	-3.612	3.03E-04	2.24E-02
Dmel_CG31198	57.349	-5.117	1.419	-3.605	3.12E-04	2.29E-02
Dmel_CG13604	682.846	0.752	0.209	3.601	3.17E-04	2.29E-02
Dmel_CG31897	145.916	-0.865	0.241	-3.596	3.24E-04	2.29E-02
Dmel_CG32474	120.332	-1.621	0.451	-3.592	3.29E-04	2.29E-02
Dmel_CG4909	2833.384	0.845	0.235	3.590	3.30E-04	2.29E-02
Dmel_CG5596	653.286	-1.482	0.413	-3.590	3.30E-04	2.29E-02
Dmel_CG17716	19.533	-2.076	0.578	-3.590	3.31E-04	2.29E-02
Dmel_CG32017	173.619	-1.268	0.353	-3.589	3.32E-04	2.29E-02
Dmel_CG5867	86.914	-1.403	0.391	-3.588	3.33E-04	2.29E-02
Dmel_CG42309	229.779	-1.694	0.472	-3.587	3.35E-04	2.29E-02
Dmel_CG7128	682.992	1.249	0.348	3.586	3.35E-04	2.29E-02
Dmel_CG43758	309.775	-1.157	0.323	-3.586	3.36E-04	2.29E-02
Dmel_CG5445	1662.056	0.563	0.157	3.579	3.46E-04	2.33E-02

Dmel_CG3613	1120.960	0.860	0.240	3.577	3.47E-04	2.33E-02
Dmel_CG10364	1000.208	1.068	0.299	3.575	3.50E-04	2.33E-02
Dmel_CG1024	621.198	0.818	0.229	3.574	3.51E-04	2.33E-02
Dmel_CG8165	279.924	0.513	0.144	3.572	3.54E-04	2.34E-02
Dmel_CG8585	391.292	-1.143	0.320	-3.569	3.58E-04	2.35E-02
Dmel_CG3171	1422.762	0.794	0.223	3.561	3.69E-04	2.41E-02
Dmel_CG18536	49.200	-1.180	0.332	-3.556	3.77E-04	2.44E-02
Dmel_CG5038	341.144	0.614	0.173	3.554	3.80E-04	2.45E-02
Dmel_CG7083	1256.892	0.546	0.154	3.550	3.85E-04	2.46E-02
Dmel_CG34445	67.998	-1.639	0.462	-3.549	3.87E-04	2.46E-02
Dmel_CG9297	38.481	-1.620	0.458	-3.539	4.01E-04	2.54E-02
Dmel_CG15443	379.439	0.676	0.191	3.537	4.05E-04	2.55E-02
Dmel_CG1825	2353.709	1.329	0.376	3.532	4.13E-04	2.56E-02
Dmel_CG43664	825.006	0.783	0.222	3.531	4.14E-04	2.56E-02
Dmel_CR46482	19457.346	-1.042	0.295	-3.531	4.14E-04	2.56E-02
Dmel_CG13977	13.266	-2.838	0.804	-3.529	4.16E-04	2.56E-02
Dmel_CG4608	113.986	-1.087	0.308	-3.525	4.24E-04	2.59E-02
Dmel_CG30195	12.984	-2.196	0.623	-3.523	4.26E-04	2.59E-02
Dmel_CG14026	3581.522	0.351	0.100	3.521	4.30E-04	2.60E-02
Dmel_CG12106	309.494	0.865	0.246	3.515	4.40E-04	2.61E-02
Dmel_CG2184	1690.958	-1.347	0.383	-3.514	4.41E-04	2.61E-02
Dmel_CR33628	2075.290	-2.268	0.646	-3.512	4.45E-04	2.61E-02
Dmel_CR33921	2075.290	-2.268	0.646	-3.512	4.45E-04	2.61E-02
Dmel_CG7997	780.900	0.535	0.152	3.511	4.46E-04	2.61E-02
Dmel_CG1455	14.264	-1.917	0.546	-3.511	4.46E-04	2.61E-02
Dmel_CG9796	5516.729	0.623	0.178	3.503	4.60E-04	2.67E-02
Dmel_CG9057	16761.326	1.152	0.329	3.502	4.62E-04	2.67E-02
Dmel_CG15138	159.816	-1.071	0.306	-3.498	4.68E-04	2.68E-02
Dmel_CG18102	3933.133	0.390	0.112	3.498	4.69E-04	2.68E-02
Dmel_CG44007	82.476	-1.583	0.453	-3.494	4.76E-04	2.71E-02
Dmel_CG9423	10906.330	0.593	0.170	3.489	4.85E-04	2.73E-02
Dmel_CG14711	1052.838	0.629	0.180	3.488	4.86E-04	2.73E-02

Dmel_CG6957	112.117	-1.274	0.366	-3.485	4.92E-04	2.75E-02
Dmel_CG6202	3774.453	0.437	0.125	3.484	4.95E-04	2.75E-02
Dmel_CG12099	2772.921	0.627	0.180	3.483	4.96E-04	2.75E-02
Dmel_CG6927	4422.478	0.656	0.189	3.479	5.04E-04	2.76E-02
Dmel_CG1894	11.141	-1.881	0.541	-3.479	5.04E-04	2.76E-02
Dmel_CG5439	666.759	0.731	0.210	3.475	5.11E-04	2.78E-02
Dmel_CG3407	971.487	0.648	0.187	3.473	5.15E-04	2.78E-02
Dmel_CG12220	492.880	-1.064	0.306	-3.473	5.16E-04	2.78E-02
Dmel_CG4622	759.225	0.889	0.256	3.471	5.18E-04	2.78E-02
Dmel_CG6658	157.093	-1.473	0.425	-3.464	5.32E-04	2.85E-02
Dmel_CG18549	1584.276	0.459	0.133	3.462	5.35E-04	2.85E-02
Dmel_CG6930	714.992	-0.856	0.247	-3.459	5.41E-04	2.87E-02
Dmel_CG2072	1813.889	0.512	0.148	3.457	5.47E-04	2.87E-02
Dmel_CG8023	8.117	-2.756	0.798	-3.454	5.51E-04	2.87E-02
Dmel_CG3163	236.299	0.760	0.220	3.454	5.52E-04	2.87E-02
Dmel_CG12110	4136.182	0.530	0.154	3.451	5.58E-04	2.87E-02
Dmel_CG32641	631.055	-0.993	0.288	-3.450	5.60E-04	2.87E-02
Dmel_CG6357	882.290	-0.763	0.221	-3.450	5.60E-04	2.87E-02
Dmel_CG9155	396.786	-0.909	0.264	-3.450	5.61E-04	2.87E-02
Dmel_CG33344	7.747	-2.076	0.602	-3.449	5.63E-04	2.87E-02
Dmel_CG8316	213.397	-0.967	0.281	-3.447	5.67E-04	2.87E-02
Dmel_CG6665	468.601	0.565	0.164	3.444	5.74E-04	2.89E-02
Dmel_CG6040	548.589	-0.925	0.269	-3.441	5.81E-04	2.92E-02
Dmel_CG4274	2581.259	1.038	0.302	3.438	5.86E-04	2.93E-02
Dmel_CG7957	723.957	0.726	0.211	3.436	5.90E-04	2.93E-02
Dmel_CG4395	13.227	2.039	0.594	3.432	5.99E-04	2.93E-02
Dmel_CG13941	124.885	-0.800	0.233	-3.431	6.02E-04	2.93E-02
Dmel_CG6121	978.359	0.918	0.268	3.430	6.04E-04	2.93E-02
Dmel_CG4951	1445.689	0.530	0.155	3.428	6.09E-04	2.93E-02
Dmel_CG34200	556.763	-1.353	0.395	-3.426	6.12E-04	2.93E-02
Dmel_CR45132	40.764	-0.987	0.288	-3.426	6.14E-04	2.93E-02
Dmel_CG7289	1206.822	0.653	0.191	3.425	6.15E-04	2.93E-02

Dmel_CG7157	83.139	-1.164	0.340	-3.424	6.17E-04	2.93E-02
Dmel_CG11737	998.601	0.956	0.279	3.423	6.18E-04	2.93E-02
Dmel_CG6398	1931.984	0.944	0.276	3.423	6.18E-04	2.93E-02
Dmel_CG4686	727.346	0.684	0.200	3.423	6.19E-04	2.93E-02
Dmel_CG18642	441.573	0.918	0.268	3.421	6.25E-04	2.94E-02
Dmel_CG15201	9.880	-2.006	0.587	-3.420	6.27E-04	2.94E-02
Dmel_CG11210	1947.088	0.651	0.191	3.417	6.33E-04	2.94E-02
Dmel_CG1664	3508.885	0.707	0.207	3.417	6.34E-04	2.94E-02
Dmel_CG6962	1700.725	0.814	0.239	3.414	6.40E-04	2.94E-02
Dmel_CG10825	890.498	0.655	0.192	3.414	6.41E-04	2.94E-02
Dmel_CG13506	177.409	-1.083	0.317	-3.413	6.41E-04	2.94E-02
Dmel_CG34392	202.441	1.421	0.416	3.413	6.43E-04	2.94E-02
Dmel_CG4620	4335.134	0.639	0.187	3.411	6.47E-04	2.94E-02
Dmel_CG44325	724.990	-0.784	0.230	-3.405	6.62E-04	2.99E-02
Dmel_CG14946	22.832	-1.745	0.513	-3.403	6.66E-04	2.99E-02
Dmel_CG17084	133.181	-1.112	0.327	-3.403	6.66E-04	2.99E-02
Dmel_CG41265	266.240	-1.196	0.352	-3.398	6.78E-04	3.03E-02
Dmel_CG17149	1566.784	0.898	0.264	3.396	6.83E-04	3.04E-02
Dmel_CG7772	674.473	0.756	0.223	3.393	6.91E-04	3.04E-02
Dmel_CG4260	7777.907	0.482	0.142	3.393	6.91E-04	3.04E-02
Dmel_CG32115	14.590	-1.894	0.558	-3.393	6.91E-04	3.04E-02
Dmel_CR42451	792.575	-1.170	0.345	-3.391	6.96E-04	3.04E-02
Dmel_CG30170	20.719	-1.855	0.547	-3.391	6.96E-04	3.04E-02
Dmel_CG4262	252.039	-0.841	0.248	-3.387	7.07E-04	3.07E-02
Dmel_CG34133	4321.118	0.566	0.167	3.383	7.18E-04	3.11E-02
Dmel_CG12942	1863.699	0.587	0.174	3.381	7.23E-04	3.12E-02
Dmel_CG5725	1904.398	0.734	0.217	3.377	7.33E-04	3.14E-02
Dmel_CG1915	6242.146	-1.045	0.310	-3.376	7.34E-04	3.14E-02
Dmel_CG33147	9.630	-2.113	0.626	-3.374	7.40E-04	3.15E-02
Dmel_CG32506	9.534	-2.114	0.627	-3.372	7.46E-04	3.16E-02
Dmel_CG4267	559.666	0.880	0.261	3.371	7.48E-04	3.16E-02
Dmel_CG12391	1629.747	0.738	0.219	3.367	7.61E-04	3.20E-02

Dmel_CG10309	2135.421	1.285	0.382	3.366	7.62E-04	3.20E-02
Dmel_CG15848	101.067	-1.331	0.396	-3.364	7.68E-04	3.21E-02
Dmel_CG1911	1431.817	0.860	0.256	3.359	7.81E-04	3.25E-02
Dmel_CG8051	10.915	-1.571	0.468	-3.354	7.96E-04	3.30E-02
Dmel_CG33103	1003.050	-1.419	0.424	-3.351	8.04E-04	3.32E-02
Dmel_CG6207	2745.065	0.708	0.211	3.350	8.07E-04	3.32E-02
Dmel_CG32452	1457.804	0.813	0.243	3.349	8.12E-04	3.33E-02
Dmel_CG10240	935.082	0.709	0.212	3.347	8.18E-04	3.34E-02
Dmel_CR43626	142.895	-1.241	0.371	-3.346	8.20E-04	3.34E-02
Dmel_CG5222	595.645	0.896	0.268	3.344	8.27E-04	3.35E-02
Dmel_CG17462	1947.213	0.930	0.279	3.337	8.46E-04	3.39E-02
Dmel_CG2765	1659.825	0.624	0.187	3.337	8.48E-04	3.39E-02
Dmel_CG4029	5627.255	0.658	0.197	3.336	8.50E-04	3.39E-02
Dmel_CG12118	916.548	0.632	0.189	3.336	8.51E-04	3.39E-02
Dmel_CG4978	3421.524	0.862	0.259	3.334	8.55E-04	3.39E-02
Dmel_CG4821	225.884	-0.994	0.298	-3.332	8.62E-04	3.39E-02
Dmel_CG3041	913.876	0.915	0.275	3.332	8.63E-04	3.39E-02
Dmel_CG9712	1406.203	0.729	0.219	3.331	8.64E-04	3.39E-02
Dmel_CG11975	684.605	0.676	0.203	3.331	8.66E-04	3.39E-02
Dmel_CG44008	16.277	-1.804	0.542	-3.329	8.70E-04	3.40E-02
Dmel_CG6536	115.126	-1.090	0.328	-3.325	8.84E-04	3.43E-02
Dmel_CG11367	593.483	1.063	0.320	3.325	8.86E-04	3.43E-02
Dmel_CG17839	101.907	-1.084	0.326	-3.324	8.88E-04	3.43E-02
Dmel_CG8282	2013.871	0.529	0.159	3.322	8.94E-04	3.44E-02
Dmel_CG11804	8263.425	0.502	0.151	3.320	9.00E-04	3.44E-02
Dmel_CG8389	831.413	0.533	0.161	3.318	9.07E-04	3.44E-02
Dmel_CG9470	1461.555	-1.099	0.331	-3.317	9.09E-04	3.44E-02
Dmel_CG42253	66.402	-1.475	0.445	-3.316	9.14E-04	3.44E-02
Dmel_CG1674	540.658	-1.334	0.402	-3.316	9.14E-04	3.44E-02
Dmel_CG8411	7127.715	0.438	0.132	3.315	9.16E-04	3.44E-02
Dmel_CG5177	88.185	-1.824	0.550	-3.315	9.18E-04	3.44E-02
Dmel_CG34438	3017.459	0.722	0.218	3.311	9.29E-04	3.47E-02

Dmel_CG5602	1070.389	0.892	0.269	3.310	9.34E-04	3.48E-02
Dmel_CG5807	2244.113	0.476	0.144	3.308	9.39E-04	3.49E-02
Dmel_CG7107	460.078	-1.355	0.410	-3.303	9.56E-04	3.54E-02
Dmel_CG10895	11736.045	0.416	0.126	3.302	9.61E-04	3.54E-02
Dmel_CG7404	1099.831	0.354	0.107	3.299	9.69E-04	3.56E-02
Dmel_CG8994	15778.334	0.610	0.185	3.297	9.79E-04	3.58E-02
Dmel_CR43314	390.005	-1.144	0.347	-3.294	9.88E-04	3.61E-02
Dmel_CG10126	206.811	-1.209	0.367	-3.293	9.91E-04	3.61E-02
Dmel_CG34431	161.743	-0.853	0.259	-3.291	9.99E-04	3.62E-02
Dmel_CG9203	680.819	0.559	0.170	3.290	1.00E-03	3.62E-02
Dmel_CG9739	548.694	-1.009	0.307	-3.288	1.01E-03	3.63E-02
Dmel_CG31469	13.182	-1.602	0.487	-3.287	1.01E-03	3.63E-02
Dmel_CG9594	2785.003	0.659	0.201	3.285	1.02E-03	3.65E-02
Dmel_CG12004	4534.896	0.695	0.212	3.284	1.03E-03	3.65E-02
Dmel_CG9432	408.363	-1.247	0.380	-3.283	1.03E-03	3.65E-02
Dmel_CG4405	155.934	-1.333	0.406	-3.281	1.03E-03	3.66E-02
Dmel_CG13403	9.694	-2.267	0.691	-3.279	1.04E-03	3.68E-02
Dmel_CG4788	695.972	0.809	0.247	3.276	1.05E-03	3.70E-02
Dmel_CG42259	266.650	-1.368	0.418	-3.273	1.06E-03	3.72E-02
Dmel_CG1019	287.029	-0.997	0.305	-3.273	1.07E-03	3.72E-02
Dmel_CG4615	903.993	0.844	0.258	3.268	1.08E-03	3.78E-02
Dmel_CG3651	2573.552	0.462	0.141	3.266	1.09E-03	3.79E-02
Dmel_CG2990	1142.823	0.655	0.201	3.265	1.10E-03	3.79E-02
Dmel_CG5180	943.481	1.088	0.333	3.264	1.10E-03	3.79E-02
Dmel_CG8440	4400.552	0.398	0.122	3.263	1.10E-03	3.80E-02
Dmel_CR46254	14.144	-1.647	0.505	-3.261	1.11E-03	3.81E-02
Dmel_CG9123	285.710	0.970	0.298	3.259	1.12E-03	3.82E-02
Dmel_CG32758	1103.252	-0.563	0.173	-3.254	1.14E-03	3.88E-02
Dmel_CG30418	100.457	-1.346	0.414	-3.247	1.16E-03	3.94E-02
Dmel_CG31926	1397.870	2.466	0.759	3.247	1.17E-03	3.94E-02
Dmel_CG4139	67.549	-1.192	0.368	-3.243	1.18E-03	3.96E-02
Dmel_CG1109	2597.079	0.493	0.152	3.243	1.18E-03	3.96E-02

Dmel_CR45941	8.758	-2.186	0.674	-3.243	1.18E-03	3.96E-02
Dmel_CG11979	478.991	-1.044	0.322	-3.240	1.19E-03	3.96E-02
Dmel_CG33556	69.075	-1.477	0.456	-3.239	1.20E-03	3.96E-02
Dmel_CG8400	3643.172	0.672	0.207	3.239	1.20E-03	3.96E-02
Dmel_CG33720	267.591	-1.014	0.313	-3.237	1.21E-03	3.96E-02
Dmel_CG34250	185.219	-1.312	0.405	-3.237	1.21E-03	3.96E-02
Dmel_CG10387	2509.834	0.619	0.191	3.237	1.21E-03	3.96E-02
Dmel_CR44953	22.110	-1.516	0.468	-3.237	1.21E-03	3.96E-02
Dmel_CG6711	1884.750	0.427	0.132	3.236	1.21E-03	3.96E-02
Dmel_CG5938	1070.366	0.735	0.227	3.234	1.22E-03	3.98E-02
Dmel_CG12275	39.817	-1.912	0.592	-3.230	1.24E-03	4.03E-02
Dmel_CG16947	157.907	1.077	0.333	3.229	1.24E-03	4.03E-02
Dmel_CG17292	1474.083	0.641	0.199	3.224	1.26E-03	4.06E-02
Dmel_CG43273	105.193	-0.861	0.267	-3.224	1.27E-03	4.06E-02
Dmel_CG32096	237.586	-0.892	0.277	-3.223	1.27E-03	4.06E-02
Dmel_CG6329	64.231	-1.629	0.505	-3.223	1.27E-03	4.06E-02
Dmel_CG31753	71.034	-1.431	0.445	-3.216	1.30E-03	4.16E-02
Dmel_CG4433	1942.289	0.732	0.228	3.213	1.31E-03	4.18E-02
Dmel_CG42584	108.987	-1.531	0.477	-3.212	1.32E-03	4.18E-02
Dmel_CG4145	1028.805	-1.141	0.355	-3.212	1.32E-03	4.18E-02
Dmel_CG10063	16.237	-1.568	0.488	-3.209	1.33E-03	4.19E-02
Dmel_CG11098	1467.654	-0.416	0.130	-3.209	1.33E-03	4.19E-02
Dmel_CG32795	1510.068	0.441	0.137	3.208	1.34E-03	4.19E-02
Dmel_CG15435	899.900	0.762	0.238	3.207	1.34E-03	4.20E-02
Dmel_CG10570	76.217	-1.661	0.518	-3.203	1.36E-03	4.23E-02
Dmel_CG42502	76.217	-1.661	0.518	-3.203	1.36E-03	4.23E-02
Dmel_CR44042	410.787	-0.827	0.259	-3.196	1.40E-03	4.33E-02
Dmel_CG17360	1450.158	0.628	0.197	3.193	1.41E-03	4.36E-02
Dmel_CG3348	150.597	-1.169	0.366	-3.191	1.42E-03	4.36E-02
Dmel_CG11007	1087.406	0.493	0.154	3.190	1.42E-03	4.36E-02
Dmel_CG5905	30.547	-1.470	0.461	-3.190	1.43E-03	4.36E-02
Dmel_CG5181	242.756	1.037	0.325	3.189	1.43E-03	4.36E-02

Dmel_CG9046	18333.540	1.340	0.420	3.189	1.43E-03	4.36E-02
Dmel_CG9836	704.353	-0.686	0.215	-3.183	1.46E-03	4.42E-02
Dmel_CG12093	455.447	0.752	0.236	3.182	1.46E-03	4.42E-02
Dmel_CG2993	277.855	0.974	0.306	3.181	1.47E-03	4.42E-02
Dmel_CG10396	15.080	-1.532	0.482	-3.181	1.47E-03	4.42E-02
Dmel_CG9984	1897.020	0.676	0.213	3.180	1.47E-03	4.42E-02
Dmel_CG3403	2318.502	0.939	0.296	3.177	1.49E-03	4.45E-02
Dmel_CG31807	141.606	-0.932	0.293	-3.176	1.49E-03	4.45E-02
Dmel_CG46339	356.917	-0.968	0.305	-3.175	1.50E-03	4.45E-02
Dmel_CG7272	93.577	-0.737	0.232	-3.174	1.50E-03	4.45E-02
Dmel_CG5939	354.903	-1.070	0.337	-3.174	1.50E-03	4.45E-02
Dmel_CG10630	13.277	-2.459	0.775	-3.173	1.51E-03	4.45E-02
Dmel_CG11723	1410.373	0.585	0.184	3.172	1.51E-03	4.45E-02
Dmel_CR45897	10.683	-1.749	0.551	-3.171	1.52E-03	4.45E-02
Dmel_CG7930	574.112	-1.313	0.414	-3.171	1.52E-03	4.45E-02
Dmel_CG31365	1258.248	0.797	0.251	3.170	1.52E-03	4.45E-02
Dmel_CG13434	94.002	-0.939	0.297	-3.166	1.55E-03	4.50E-02
Dmel_CG42599	266.112	-1.010	0.319	-3.165	1.55E-03	4.50E-02
Dmel_CG7837	1051.188	0.740	0.234	3.164	1.56E-03	4.52E-02
Dmel_CG8811	8990.635	0.483	0.153	3.161	1.57E-03	4.53E-02
Dmel_CG3836	2301.302	0.722	0.228	3.161	1.57E-03	4.53E-02
Dmel_CG2330	77.759	-1.493	0.472	-3.160	1.58E-03	4.54E-02
Dmel_CG44246	646.353	0.700	0.222	3.158	1.59E-03	4.55E-02
Dmel_CG15721	6051.069	1.209	0.383	3.155	1.61E-03	4.59E-02
Dmel_CG18507	93.141	1.199	0.380	3.154	1.61E-03	4.59E-02
Dmel_CG32212	22.799	-1.623	0.515	-3.152	1.62E-03	4.60E-02
Dmel_CG32320	96.491	-1.370	0.435	-3.152	1.62E-03	4.60E-02
Dmel_CR44291	23.840	-1.339	0.425	-3.150	1.63E-03	4.62E-02
Dmel_CG8933	5203.559	0.513	0.163	3.145	1.66E-03	4.68E-02
Dmel_CG9379	238.648	-0.827	0.263	-3.145	1.66E-03	4.68E-02
Dmel_CG3065	790.124	1.218	0.387	3.144	1.67E-03	4.68E-02
Dmel_CG4214	1980.475	0.474	0.151	3.139	1.70E-03	4.75E-02

Dmel_CG8066	345.072	-0.741	0.236	-3.134	1.72E-03	4.81E-02
Dmel_CG1448	1370.609	-0.865	0.277	-3.128	1.76E-03	4.89E-02
Dmel_CG1745	4690.633	0.672	0.215	3.128	1.76E-03	4.89E-02
Dmel_CG7999	1414.777	0.475	0.152	3.126	1.77E-03	4.89E-02
Dmel_CG8114	6061.248	0.364	0.116	3.126	1.78E-03	4.89E-02
Dmel_CG12763	27.517	-3.900	1.248	-3.125	1.78E-03	4.89E-02
Dmel_CG5083	2062.330	0.757	0.242	3.123	1.79E-03	4.92E-02
Dmel_CG1487	3370.356	0.413	0.132	3.121	1.80E-03	4.93E-02
Dmel_CG5907	136.047	-1.175	0.377	-3.121	1.80E-03	4.93E-02
Dmel_CG14162	1349.763	0.684	0.219	3.120	1.81E-03	4.93E-02
Dmel_CG30118	7132.753	0.703	0.226	3.118	1.82E-03	4.96E-02
Dmel_CG9078	3970.323	0.435	0.140	3.115	1.84E-03	4.98E-02
Dmel_CG2249	2474.631	-0.901	0.289	-3.114	1.84E-03	4.98E-02
Dmel_CG9220	125.885	-0.824	0.265	-3.113	1.85E-03	4.98E-02
Dmel_CG8548	6621.909	0.439	0.141	3.113	1.85E-03	4.98E-02
Dmel_CG8338	594.660	-1.008	0.324	-3.112	1.86E-03	4.98E-02
Dmel_CG4407	1040.887	0.405	0.130	3.111	1.86E-03	4.98E-02
Dmel_CG3671	2152.230	0.679	0.218	3.111	1.86E-03	4.98E-02
Dmel_CR46037	8285.229	-1.223	0.393	-3.111	1.87E-03	4.98E-02
Dmel_CG9342	904.312	0.552	0.178	3.106	1.90E-03	5.03E-02
Dmel_CG33722	1440.994	0.563	0.181	3.106	1.90E-03	5.03E-02
Dmel_CG12242	24.371	-1.777	0.572	-3.105	1.90E-03	5.03E-02
Dmel_CG34333	20400.941	1.614	0.520	3.104	1.91E-03	5.04E-02
Dmel_CG15431	62.781	-0.872	0.281	-3.104	1.91E-03	5.04E-02
Dmel_CG32850	1595.721	-0.790	0.255	-3.100	1.93E-03	5.08E-02
Dmel_CG1311	1395.571	0.650	0.210	3.097	1.96E-03	5.13E-02
Dmel_CG12404	1283.354	0.419	0.136	3.095	1.97E-03	5.14E-02
Dmel_CR45146	7.571	-1.851	0.598	-3.094	1.98E-03	5.15E-02
Dmel_CG2934	5007.021	0.586	0.190	3.092	1.99E-03	5.17E-02
Dmel_CG14621	2331.371	0.753	0.244	3.087	2.02E-03	5.24E-02
Dmel_CG15094	334.092	0.800	0.259	3.084	2.04E-03	5.30E-02
Dmel_CG11263	812.988	-0.804	0.261	-3.078	2.09E-03	5.38E-02

Dmel_CG5848	5306.631	0.850	0.276	3.077	2.09E-03	5.38E-02
Dmel_CG3227	1353.712	1.111	0.361	3.077	2.09E-03	5.38E-02
Dmel_CG1569	2450.503	0.625	0.203	3.075	2.10E-03	5.39E-02
Dmel_CG3961	102.114	-0.958	0.312	-3.075	2.11E-03	5.39E-02
Dmel_CG7692	1472.997	0.524	0.170	3.074	2.11E-03	5.39E-02
Dmel_CG16840	80.024	-1.618	0.526	-3.073	2.12E-03	5.40E-02
Dmel_CG5857	1494.849	0.656	0.214	3.071	2.13E-03	5.42E-02
Dmel_CG34163	207.187	-1.039	0.338	-3.069	2.15E-03	5.44E-02

table S18. *D. melanogaster* genes Wald Test significant results for ~Infection vs ~Genotype+Infection+Genotype*Infection
