# Supplementary material for: Wolbachia endosymbionts manipulate the self-renewal and differentiation of germline stem cells to reinforce fertility of their fruit fly host
Source: PLoS Biol. 2023 Oct 24;21(10):e3002335. doi: 10.1371/journal.pbio.3002335 (PMC10597519; doi:10.1371/journal.pbio.3002335)
Supplement: S19 Table — (PDF) [file pbio.3002335.s034.pdf]

gene_id	baseMean	log2FoldChange	lfcSE	stat	pvalue	padj
Dmel_CG32834	13.460	-21.521	2.735	-7.868	3.61E-15	3.64E-11
Dmel_CG2187	500.405	-2.857	0.369	-7.751	9.12E-15	4.59E-11
Dmel_CG3104	56.006	2.402	0.476	5.043	4.59E-07	1.54E-03
Dmel_CG10045	10067.445	-4.248	0.867	-4.901	9.52E-07	2.40E-03
Dmel_CG7404	1099.831	-0.694	0.152	-4.557	5.20E-06	1.05E-02
Dmel_CG5937	33.106	2.974	0.677	4.394	1.11E-05	1.86E-02
Dmel_CG9650	454.888	1.566	0.360	4.350	1.36E-05	1.96E-02
Dmel_CG8805	2531.220	-0.929	0.218	-4.270	1.95E-05	2.46E-02
Dmel_CG15599	55.457	3.314	0.789	4.200	2.67E-05	2.61E-02
Dmel_CG9155	396.786	1.554	0.373	4.164	3.12E-05	2.61E-02
Dmel_CG6178	2685.460	-0.699	0.168	-4.163	3.14E-05	2.61E-02
Dmel_CG17167	307.196	-1.705	0.411	-4.151	3.31E-05	2.61E-02
Dmel_CG13772	15.539	3.746	0.905	4.139	3.48E-05	2.61E-02
Dmel_CG8388	689.979	-0.713	0.173	-4.121	3.78E-05	2.61E-02
Dmel_CG3157	1113.612	-1.129	0.275	-4.114	3.89E-05	2.61E-02
Dmel_CG43079	631.857	2.061	0.503	4.096	4.21E-05	2.65E-02
Dmel_CG18870	2820.357	-0.823	0.202	-4.070	4.70E-05	2.78E-02
Dmel_CG18522	301.389	2.247	0.555	4.048	5.16E-05	2.89E-02
Dmel_CG12002	217.218	2.463	0.611	4.029	5.60E-05	2.93E-02
Dmel_CG31901	28.374	3.961	0.985	4.020	5.82E-05	2.93E-02
Dmel_CG10593	2009.122	-2.174	0.544	-3.993	6.52E-05	3.09E-02
Dmel_CG10573	175.250	1.302	0.327	3.985	6.75E-05	3.09E-02
Dmel_CR43461	15.553	2.695	0.680	3.965	7.34E-05	3.12E-02
Dmel_CG16762	7.124	4.634	1.172	3.955	7.66E-05	NA
Dmel_CG13871	15.439	-5.015	1.269	-3.952	7.75E-05	3.12E-02
Dmel_CG8663	114.131	2.257	0.573	3.941	8.11E-05	3.12E-02
Dmel_CG30046	65.427	-1.745	0.444	-3.929	8.54E-05	3.12E-02
Dmel_CR44115	21.000	4.551	1.163	3.913	9.12E-05	3.12E-02
Dmel_CG31973	444.390	1.434	0.367	3.906	9.40E-05	3.12E-02
Dmel_CG6542	4210.347	-0.815	0.209	-3.903	9.51E-05	3.12E-02

Dmel_CG5870	1313.514	1.321	0.339	3.894	9.84E-05	3.12E-02
Dmel_CG9610	35.323	2.525	0.648	3.893	9.90E-05	3.12E-02
Dmel_CG10726	2404.031	-1.042	0.268	-3.886	1.02E-04	3.12E-02
Dmel_CG15814	1716.320	-0.877	0.227	-3.871	1.09E-04	3.12E-02
Dmel_CG8256	121.600	1.720	0.445	3.865	1.11E-04	3.12E-02
Dmel_CG7660	20442.794	-0.690	0.179	-3.861	1.13E-04	3.12E-02
Dmel_CG9073	36.796	2.621	0.679	3.858	1.14E-04	3.12E-02
Dmel_CG32082	143.362	1.635	0.424	3.854	1.16E-04	3.12E-02
Dmel_CG9888	413.992	1.902	0.494	3.851	1.18E-04	3.12E-02
Dmel_CG9220	125.885	1.424	0.371	3.840	1.23E-04	3.18E-02
Dmel_CG9772	2989.847	-0.860	0.224	-3.833	1.27E-04	3.19E-02
Dmel_CG12283	681.554	1.430	0.375	3.819	1.34E-04	3.29E-02
Dmel_CG5252	4020.919	-0.666	0.175	-3.808	1.40E-04	3.37E-02
Dmel_CG12763	27.517	7.141	1.880	3.799	1.45E-04	3.41E-02
Dmel_CR44472	23.272	-2.646	0.702	-3.772	1.62E-04	3.69E-02
Dmel_CG43758	309.775	1.714	0.456	3.760	1.70E-04	3.69E-02
Dmel_CG2275	1215.191	-1.179	0.314	-3.754	1.74E-04	3.69E-02
Dmel_CG9559	4504.461	-1.078	0.287	-3.754	1.74E-04	3.69E-02
Dmel_CG1977	2140.679	1.065	0.284	3.752	1.76E-04	3.69E-02
Dmel_CG12375	831.121	-0.777	0.208	-3.739	1.85E-04	3.80E-02
Dmel_CG40813	5.158	8.173	2.197	3.720	1.99E-04	NA
Dmel_CG3812	1006.282	-0.794	0.215	-3.698	2.17E-04	4.33E-02
Dmel_CR46350	65.211	1.927	0.521	3.696	2.19E-04	4.33E-02
Dmel_CG3879	12.492	3.044	0.827	3.683	2.31E-04	4.43E-02
Dmel_CR45530	16.389	2.296	0.624	3.680	2.33E-04	4.43E-02
Dmel_CG12477	8.616	5.136	1.396	3.679	2.34E-04	NA
Dmel_CG17292	1474.083	-1.032	0.281	-3.674	2.39E-04	4.45E-02
Dmel_CG2807	1130.688	0.911	0.249	3.652	2.60E-04	4.76E-02
Dmel_CG9379	238.648	1.353	0.371	3.643	2.69E-04	4.79E-02
Dmel_CG17927	1300.359	2.092	0.574	3.641	2.71E-04	4.79E-02
Dmel_CG6006	523.400	1.551	0.427	3.636	2.77E-04	4.81E-02
Dmel_CG3407	971.487	-0.958	0.264	-3.625	2.89E-04	4.82E-02

Dmel_CG1462	323.655	1.723	0.476	3.621	2.94E-04	4.82E-02
Dmel_CG13000	15.067	2.743	0.759	3.615	3.00E-04	4.82E-02
Dmel_CG18375	181.968	1.050	0.291	3.614	3.01E-04	4.82E-02
Dmel_CG8262	752.433	-1.356	0.375	-3.614	3.02E-04	4.82E-02
Dmel_CG6227	371.074	1.175	0.326	3.604	3.13E-04	4.93E-02
Dmel_CG18549	1584.276	-0.675	0.188	-3.597	3.22E-04	4.93E-02
Dmel_CG5644	28.092	-2.406	0.670	-3.592	3.29E-04	4.93E-02
Dmel_CG34323	38.530	2.239	0.624	3.587	3.34E-04	4.93E-02
Dmel_CG17078	3064.101	-1.210	0.338	-3.584	3.39E-04	4.93E-02
Dmel_CR46481	1090.588	1.301	0.363	3.581	3.43E-04	4.93E-02
Dmel_CG3635	29.922	2.696	0.753	3.578	3.47E-04	4.93E-02
Dmel_CG9901	6113.781	-0.527	0.147	-3.577	3.47E-04	4.93E-02
Dmel_CG9707	1943.581	-0.814	0.228	-3.569	3.59E-04	5.02E-02
Dmel_CG3171	1422.762	-1.120	0.315	-3.552	3.82E-04	5.27E-02
Dmel_CG7002	154.856	2.295	0.648	3.539	4.02E-04	5.27E-02
Dmel_CG4620	4335.134	-0.936	0.265	-3.538	4.04E-04	5.27E-02
Dmel_CG6202	3774.453	-0.627	0.177	-3.537	4.04E-04	5.27E-02
Dmel_CG8411	7127.715	-0.661	0.187	-3.536	4.06E-04	5.27E-02
Dmel_CG11941	16.019	3.035	0.859	3.535	4.08E-04	5.27E-02
Dmel_CG12110	4136.182	-0.767	0.217	-3.531	4.14E-04	5.28E-02
Dmel_CG17839	101.907	1.620	0.459	3.526	4.22E-04	5.29E-02
Dmel_CG1024	621.198	-1.141	0.324	-3.521	4.30E-04	5.29E-02
Dmel_CG5445	1662.056	-0.783	0.223	-3.518	4.36E-04	5.29E-02
Dmel_CG16778	54.657	-3.076	0.875	-3.517	4.36E-04	5.29E-02
Dmel_CG5939	354.903	1.672	0.476	3.512	4.46E-04	5.34E-02

table S19. *D. melanogaster* genes with Wald Test significant results for ~Genotype*Infection vs ~Genotype+Infection+Genotype*Infection
