# Supplementary material for: Wolbachia endosymbionts manipulate the self-renewal and differentiation of germline stem cells to reinforce fertility of their fruit fly host
Source: PLoS Biol. 2023 Oct 24;21(10):e3002335. doi: 10.1371/journal.pbio.3002335 (PMC10597519; doi:10.1371/journal.pbio.3002335)
Supplement: S20 Table — (PDF) [file pbio.3002335.s035.pdf]

gene_id	baseMean	log2FoldChange	lfcSE	stat	pvalue	padj
WD_RS03770	149.412	0.486	0.160	3.043	2.34E-03	7.92E-01
WD_RS05260	22.714	-1.127	0.380	-2.969	2.99E-03	7.92E-01
WD_RS06475	10.609	1.217	0.449	2.710	6.72E-03	7.92E-01
WD_RS05810	49.056	-0.535	0.199	-2.687	7.20E-03	7.92E-01
WD_RS04825	14.115	1.302	0.486	2.678	7.41E-03	7.92E-01
WD_RS04740	25.586	-1.220	0.461	-2.646	8.13E-03	7.92E-01
WD_RS04370	80.236	-0.387	0.148	-2.622	8.73E-03	7.92E-01
WD_RS01760	8.222	1.263	0.491	2.572	1.01E-02	7.92E-01
WD_RS01300	60.484	0.563	0.223	2.519	1.18E-02	7.92E-01
WD_RS05640	10.502	-1.425	0.567	-2.513	1.20E-02	7.92E-01
WD_RS02205	25.001	-1.221	0.496	-2.464	1.37E-02	8.26E-01
WD_RS01390	25.646	0.699	0.303	2.306	2.11E-02	9.96E-01
WD_RS03775	1404.573	0.354	0.161	2.207	2.73E-02	9.96E-01
WD_RS00480	8.281	1.001	0.454	2.204	2.76E-02	9.96E-01
WD_RS05480	70.888	-0.398	0.181	-2.203	2.76E-02	9.96E-01
WD_RS01110	12.261	-0.982	0.447	-2.198	2.80E-02	9.96E-01
WD_RS03920	32.007	-0.685	0.313	-2.190	2.85E-02	9.96E-01
WD_RS05665	11.884	0.905	0.422	2.145	3.19E-02	9.96E-01
WD_RS05520	17.489	0.821	0.388	2.118	3.42E-02	9.96E-01
WD_RS01790	16.771	-1.521	0.738	-2.062	3.92E-02	9.96E-01
WD_RS01335	36.739	-0.448	0.219	-2.050	4.03E-02	9.96E-01
WD_RS00655	32.020	-0.467	0.228	-2.046	4.07E-02	9.96E-01
WD_RS04175	9.229	0.982	0.483	2.034	4.20E-02	9.96E-01
WD_RS01525	14.722	0.836	0.417	2.002	4.52E-02	9.96E-01
WD_RS01990	15.357	0.803	0.405	1.985	4.72E-02	9.96E-01
WD_RS02935	45.227	0.477	0.240	1.985	4.72E-02	9.96E-01
WD_RS02225	22.418	0.650	0.333	1.949	5.13E-02	9.96E-01

WD_RS04020	7.360	0.942	0.485	1.940	5.23E-02	9.96E-01
WD_RS03070	8.315	1.286	0.665	1.934	5.32E-02	9.96E-01

table S20. *w*Mel *Wolbachia* genes Wald Test significant results for ~Genotype vs ~1
