# Supplementary material for: Wolbachia endosymbionts manipulate the self-renewal and differentiation of germline stem cells to reinforce fertility of their fruit fly host
Source: PLoS Biol. 2023 Oct 24;21(10):e3002335. doi: 10.1371/journal.pbio.3002335 (PMC10597519; doi:10.1371/journal.pbio.3002335)
Supplement: Attachment — Submitted filename: R2R_meiP26rescue_PLoSBio.pdf [file pbio.3002335.s036.pdf]

Dear Lucas,

Thank you for your hard work finding reviewers and your helpful comments on our manuscript. We have cleared up the referencing issues, connections between the discussion and figures, and incorrect claims in the text. We appreciate these being brought to our attention. We also appreciate being given the time to fully address your and Reviewers' concerns. We have detailed our responses to all of the Reviewers' comments below, and provided summaries of the major points here:

We appreciate the comments and concerns you all had about the fecundity assays, and we are happy to inform you that all of the assays were performed in a highly controlled, parallel manner for comparability and reproducibility across genotypes and infection statuses. We have provided additional information about our experimental design and conditions in the Supplemental Methods. Furthermore, we provided the full 3000 cross fecundity dataset as a Supplemental table and we have plotted the data versus collection date in a Supplemental figure to illustrate the consistency of the data. Our continuous cross and control strategy was a deliberate choice: we wanted all of our data to be comparable and the fertility mutants produce few offspring per day. Small and highly replicated batches across many genetic crosses was a more achievable strategy for these mutants. Furthermore, we believe this data collection framework is more consistent with how the data would look from nature and we had no issues with statistical power - the *mei-P26* mutants and rescue/reinforcement phenotypes were consistently significant. Fortunately, we generated the nos:Gal4>RFP control data requested by Reviewer 2 at the same time as the original crosses (but had not included them for length considerations). So, these data are fully comparable with the nos:Gal4>mei-P26 RNAi datasets.

Given that Reviewer 1 asked us to provide the number of transcripts for knockdown vs wild-type *mei-P26* mutants and we did not feel that producing more fecundity data would be useful, we used the review period to perform dual-RNAseq on *mei-P26* knockdown and OreR wild-type ovaries. New fecundity data is not warranted because 1) it would be redundant with the data we previously generated and 2) I have started a new lab, so the new data would be discontinuous in time and space with the old dataset. In addition to using the ovary transcriptomes for quantifying *mei-P26* knockdown, we leveraged them to explore differential expression between the *mei-P26[1]* and OreR genotypes and *Wolbachia* infection states. We identify several key genes and pathways that are rescued in the *w*Mel-infected *mei-P26[1]* ovaries, including chorion protein transcripts, *bgcn,* and *tut*, which help to explain how *w*Mel mitigates the loss of *mei-P26* function.

Lastly, Reviewer 3 had an issue with our claim of "wild-type fertility increase", so we have tried to temper this in a couple of ways. First, we make more efforts to clarify that we demonstrate a fertility reinforcement phenotype, opposed to an elevation in fecundity. While we talk about fitness benefits hypothetically, we are clear to explain that we only demonstrate increased hatch rates, i.e., rescued development. In theory this could accumulate over the lifespan of the host, as their resources are more efficiently converted to offspring, but we do not demonstrate that. Second, we are clear to state that we are making claims regarding "wild-type" in regards to our OreR data only. The other "wild-type"-like strains are included because, while they do include transgenic elements, they do not harbor any fitness mutations. We are merely providing an illustrative link into how the functions that underlie *mei-P26* rescue could have been selected for in wild-type populations. It is beyond the scope of this paper to fully describe wild-type fitness benefits in the wMel-*D. melanogaster* association.

Thank you again for your time and constructive comments.

Best,
Shelbi

--
Shelbi Russell, PhD

Assistant Professor
Department of Biomolecular Engineering
University of California Santa Cruz
Sinsheimer Laboratories Rm. 427
1156 High St. Santa Cruz, CA 95064

##

Dear Dr Russell,

Thank you for your patience while your manuscript "Wolbachia endosymbionts manipulate GSC self-renewal and differentiation to enhance host fertility" was peer-reviewed at PLOS Biology. I apologize for the protracted review process for your study - in this case, for some reason, we had a bit of trouble lining up 3 reviewers in the initial stage and then I was at a conference all last week which delayed things further. Your manuscript has now been evaluated by the PLOS Biology editors, an Academic Editor with relevant expertise, and by several independent reviewers.

In light of the reviews, which you will find at the end of this email, we would like to invite you to revise the work to thoroughly address the reviewers' reports.

As you will see below, the reviewers find the work of potential interest and the analyses to be fairly comprehensive. However they have raised a number of important and overlapping concerns that would need to be thoroughly addressed before we can consider your manuscript for publication. The reviewers have highlighted that the writing and data presentation is a bit challenging to follow, noting, for example that the discussion and connection of the discussion to figures is loose, and that some statements do not seem to be aligned with relevant figures or statistics. Additionally, the authors raise concerns about the appropriateness of the control genotypes used in several experiments and the reviewers have noted that comparisons of infected and uninfected wt flies are needed as a contrast to what happens in the mutants. After discussion with the Academic Editor we think these trials should be done in parallel, which might require a repeat of the whole experiment on fertility
effects.

Given the extent of revision needed, we cannot make a decision about publication until we have seen the revised manuscript and your response to the reviewers' comments. Your revised manuscript is likely to be sent for further evaluation by all or a subset of the reviewers.

We expect to receive your revised manuscript within 3 months. Please email us (plosbiology@plos.org) if you have any questions or concerns, or would like to request an extension.

At this stage, your manuscript remains formally under active consideration at our journal; please notify us by email if you do not intend to submit a revision so that we may withdraw it.

IMPORTANT - SUBMITTING YOUR REVISION

Your revisions should address the specific points made by each reviewer. Please submit the following files along with your revised manuscript:

1. A 'Response to Reviewers' file - this should detail your responses to the editorial requests, present a point-by-point response to all of the reviewers' comments, and indicate the changes made to the manuscript.
**NOTE: In your point-by-point response to the reviewers, please provide the full context of each review. Do not selectively quote paragraphs or sentences to reply to. The entire set of reviewer comments should be present in full and each specific point should be responded to individually, point by point.*
You should also cite any additional relevant literature that has been published since the original submission and mention any additional citations in your response.

2. In addition to a clean copy of the manuscript, please also upload a 'track-changes' version of your manuscript that specifies the edits made. This should be uploaded as a "Revised Article with Changes Highlighted" file type.

Re-submission Checklist
When you are ready to resubmit your revised manuscript, please refer to this re-submission checklist: https://plos.io/Biology_Checklist

To submit a revised version of your manuscript, please go to https://www.editorialmanager.com/pbiology/ and log in as an Author. Click the link labelled 'Submissions Needing Revision' where you will find your submission record.

Please make sure to read the following important policies and guidelines while preparing your revision:

Published Peer Review
Please note while forming your response, if your article is accepted, you may have the opportunity to make the peer review history publicly available. The record will include editor decision letters (with reviews) and your responses to reviewer comments. If eligible, we will contact you to opt in or out. Please see here for more details:
https://blogs.plos.org/plos/2019/05/plos-journals-now-open-for-published-peer-review/

PLOS Data Policy
Please note that as a condition of publication PLOS' data policy (http://journals.plos.org/plosbiology/s/data-availability) requires that you make available all data used to draw the conclusions arrived at in your manuscript. If you have not already done so, you must include any data used in your manuscript either in appropriate repositories, within the body of the manuscript, or as supporting information (N.B. this includes any numerical values that were used to generate graphs, histograms etc.). For an example see here: http://www.plosbiology.org/article/info%3Adoi%2F10.1371%2Fjournal.pbio.1001908#s5

Blot and Gel Data Policy
We require the original, uncropped and minimally adjusted images supporting all blot and gel results reported in an article's figures or Supporting Information files. We will require these files before a manuscript can be accepted so please prepare them now, if you have not already uploaded them. Please carefully read our guidelines for how to prepare and upload this data:
https://journals.plos.org/plosbiology/s/figures#loc-blot-and-gel-reporting-requirements

Protocols deposition

To enhance the reproducibility of your results, we recommend that if applicable you deposit your laboratory protocols in protocols.io, where a protocol can be assigned its own identifier (DOI) such that it can be cited independently in the future. Additionally, PLOS ONE offers an option for publishing peer-reviewed Lab Protocol articles, which describe protocols hosted on protocols.io. Read more information on sharing protocols at https://plos.org/protocols?utm_medium=editorial-email&utm_source=authorletters&utm_campaign=protocols

Thank you again for your submission to our journal. We hope that our editorial process has been constructive thus far, and we welcome your feedback at any time. Please don't hesitate to contact us if you have any questions or comments.

Sincerely,

Lucas

Lucas Smith, Ph.D.
Associate Editor
PLOS Biology
lsmith@plos.org

REVIEWS:

Reviewer #1:
Since the early discovery that Wolbachia could rescue the sterility of some mutant alleles of the Sxl gene, additional studies have established Wolbachia as an important regulator of GSC maintenance and proliferation in the female germline. In this work, Russell et al. have now discovered that wMel is capable of rescuing several aspects of the complex oogenesis phenotype associated with mei-P26 knock-down or mutants in D. melanogaster. They performed a detailed analysis of these defects affecting GSC maintenance, germ cell proliferation and differentiation, as well as female fecundity and fertility. They also show that wMel is beneficial to the fertility of wild-type flies. One main interest of this work is to reveal the complexity of this critical host-symbiont interaction, with Wolbachia likely controlling several aspects of oogenesis through the expression of multiple proteins.

- Having confirmed that CI is very weak in D. mel, the authors speculate on the possibility that wMel is losing its ability to induce CI and instead develops a mutualistic interaction with its host based on increased female fertility. However, the authors should ***explain how they integrate in their model the fact that wMel is fully capable of inducing strong CI in D. simulans or Aedes mosquitoes.***

Host factors likely evolved away from enabling strong CI. Theory predicts that beneficial fecundity mutations will be selected for in both wMel and *D. melanogaster* populations (Turelli 2004), and this may indirectly undermine CI. The amazing ability of wMel to induce strong CI in distantly related hosts suggests that the bacterium's CI machinery is intact and functional. So, *D. melanogaster* must have evolved some compensatory mutations along the way.

We added a brief discussion of this (in bold) in the following section of the introduction: "However, in its native host, the fruit fly *Drosophila melanogaster*, wMel exhibits CI that rarely exceeds 50% mortality and is extremely sensitive to paternal age [14–16], as well as grandmother age/female titer [17,18]. Despite weak CI in its native

host, wMel is found at moderate to high infection frequencies in populations worldwide [19,20]. Other data suggest that these frequencies may be explained by some emergent beneficial function that increases host fitness [21–24]. **The molecular basis for these beneficial functions could be related to the loss of CI efficacy in *D. melanogaster*.** Given that wMel's use in non-native hosts relies on strong and efficient CI, it is essential that we learn the basis for its beneficial functions that could ultimately undermine CI function."

- I found quite surprising that experiments in Figure 1A-C do not include a wild-type control. The authors refers to "wild-type" (l. 127) but it is actually missing in the Figure.

Thank you for pointing out that this data presentation format was unclear/lacking. Control OreR wild-type and uninfected genotypes were run in parallel. We compared the values statistically, but thought it would clutter the presentation to include the plots. We've corrected this issue by adding the OreR wild-type data to Figure 1A-C.

- The text alludes on several occasions on the "nature" of the mei-P26[1] allele (eg l.220, l.352). However, the mei-P26 alleles used in this work are not described. mei-P26[1] actually contains a transposon insertion in the first intron of the gene, so the actual impact of this mutation on the production of mei-P26 product is unclear. The authors could measure the amount of transcripts produced by this allele and the RNAi, compared to a control.

Thank you for suggesting that we diagram the nature of the *mei-P26* mutants for the reader - we added a section to the supplemental methods and a supplemental figure (fig S2A) depicting the gene model for *mei-P26* and the alleles studied in this paper.

While the precise impact of these mutations on the *mei-P26* product is unknown, their genomic and phenotypic consequences have been thoroughly shown to be hypomorphic for *mei-P26[1],* mostly null for *mei-P26[fs1]*, and fully null for *mei-P26[mfs1]* (e.g., Page et al. 2000 - the alleles form a series: *mei-P26[1]* < *mei-P26[fs1]* (and other fs alleles) < *mei-P26[mfs1]*). We added a description of these aspects of the alleles in the Supplemental Methods.

We also sequenced the host transcriptomes among genotypes to estimate the effects of the *mei-P26[1]* allele and nos:Gal4>RNAi knockdown on *mei-P26* transcript length and abundance. These data are presented in the results and Supplemental fig S2B-E.

- It was previously reported in Starr & Cline Nature 2002 that Wolbachia is incapable of rescuing the sterility of homozygous mei-P26[fs1] females. The fact that a complete null of mei-P26 is not rescuable should be clearly mentioned in the text and discussed. Similarly, l. 120, "flies lacking Mei-P26" is likely an overstatement.

You are absolutely correct. We think the discrepancy between our reported rescue and Starr and Cline's lack of rescue is due to differences among the alleles and the higher detection sensitivity of our assays.

mei-P26[1] was created by the random insertion of a P{lacW} transposon in the first intron of *mei-P26*. The *mei-P26[fs1]* allele was derived from *mei-P26[1]* by the insertion of another P{lacW} in the first P{lacW} in *mei-P26's* first intron. Whereas, the *mei-P26[mfs1]* allele arose from a full deletion of the single insertion and ~2.5 kb of surrounding sequence. Thus the alleles are distinct, and as discussed above, exhibit different strengths.

We would rather not weigh in on *mei-P26[fs1]* rescue-ability because it is no longer available for study. Page et al. 2000 speculate that it may not be fully null. Our demonstration of *mei-P26[mfs1]*'s partial rescue indicates that rescue of a fully null allele is possible. Thus, it is impossible for us to know if our results are inconsistent with Starr and Cline, or if they would have picked up on wMel's partial rescue phenotype if they had counted eggs laid and hatched and investigated the resulting cytology.

This issue does deserve to be addressed in the text - thank you for prompting this. We've added the following section (bold) to the discussion so the reader knows that we considered this issue:

"Previously, wMel was shown to rescue the loss of Sxl [30] and Bam [31], two essential genes for two very different stages of early oogenesis: GSC maintenance/CB differentiation and TA-mitosis, respectively. These findings left unresolved how wMel suppresses these mechanistically and temporally distinct phenotypes**, and suggested that *mei-P26* was not involved.** We show **with an array of alleles and developmental assays** that infection with wMel rescues defects associated with mutant *mei-P26*, a gene required for both GSC maintenance and differentiation."

Additional comments:

- *I am not sure if male-killing should be considered as a reproductive parasitism (l. 63).*

Thank you for evaluating our classification of phenotypes. We looked into the literature to double check our usage, and male killing is consistently termed as a reproductive parasitism (e.g., Engelstädter and Hurst 2007, Hurst and Jiggins 2000, Perlmutter et al. 2019) or reproductive manipulation (e.g., Harumoto et al. 2018).

- *l. 70 "pipiensis"*

Thanks for catching this typo.

- *l.143-144: I had hard time to understand this Table E until I read l. 163*

We appreciate you pointing out that this table was not as interpretive as we had hoped. Given that you found the wording of "a 44% increase" more clear than a ratio of "mutant/WT of 1.44", we figured that percentages may be easier to interpret than proportions and changed the table in Fig 1E accordingly.

- *Fig 1J shows a perfect ovariole, yet fecundity is severely affected. A more global view of the ovary with several ovarioles could perhaps better reflect the reality.*

We updated this figure and added a supplemental figure to include a greater diversity of phenotypes, as suggested. This is indeed more consistent with the data, although rescue is quite complete in wMel-infected *mei-P26* RNAi flies. We also added a new supplemental figure (fig S5) with more *mei-P26[1]* infected and uninfected images, so the reader can get a better sense of the data. And, we added images for the *mei-P26[mfs1]* allele to Fig 1 to give a sense of the phenotypes in these ovarioles as well.

- *L. 201: the Wolbachia titers in somatic vs germ cells cannot really be appreciated in this image.*

Thank you for pointing this out. We added additional outlines and annotations to illustrate the somatic vs germline cell distinction in Fig 1H-H'.

- *L. 202: (Fig 1L-L')*

The uninfected image (M') serves as a control for the infected (L'), and is the reason we included it in the figure. We think the correct reference is to both images, and both need to be cited to include them in the text. We have clarified by including details about the control (**in bold**; with updated figure labels):

“In the D. melanogaster germarium, wMel is continuously present at high titers in both germline and somatic cells (Fig 1H**-H’, compared to control in Fig 1I-I’**),”

- *L. 210: A description of GSC and other germ cells could help.*

We added a description of *Drosophila* GSCs, but we are not clear what is meant by “other germ cells”:

“Positive pMad staining indicates the cell is responding to quiescent signals from the surrounding somatic GSC niche cells. Positive Hts staining requires localization to a single punctate spectrosome, opposed to a branching fusome [44].”

Do you perhaps mean the germline-derived cells that become the oocyte? We describe this process in detail in the coming paragraphs, and so do not think it is appropriate to describe here. We have also moved the germarium diagram up to Figure 1J.

- *L. 216: one extra "uninfected" word in the sentence.*

Thank you for pointing out this typo. We have removed the extra “uninfected”.

- *L. 230: I guess "PHH3" refers to phosphorylation of H3 serine 10.*

Yes, it is spelled out at its first mention in the main text, and we have now added this to the figure legend to aid the reader in interpreting the abbreviation.

- *L. 242: Not sure to understand what the authors mean by "to recover some of their GSCs" or l. 245 "increased their GSCs abundance".*

We actually probably mean “retain”. Although, there are mechanisms for recruiting GSCs in adults (e.g., Kai and Spradling 2003)… we aren’t trying to distinguish that here. “Recover” is mis-applied from the rescue discussion (i.e., wMel “recovered” GSC maintenance in mei-P26 mutants). We’ve changed the text for accuracy/clarity:

“Both infected and uninfected mei-P26[1] females appear to **retain** some of their GSCs as they age (aged/infected/hypomorph: 1.5 and aged/uninfected/hypomorph: 0.94, p=0.013-0.014, Wilcoxon rank sum test).“

- *L. 288: This diagram is beautiful but unfortunately wrong. It is known since King (1970) that the oocyte invariably forms from one of the two cells with four ring canals.*

Thank you so much for pointing this out. The specified oocyte was carelessly placed and I completely forgot about that important fact. The figures are now correct (including the striking image and final model figure).

- *L. 375: "this dominant-negative …". I don't see any obvious dominant-negative effect here. This should be clarified.*

We clarified this section to walk the reader through how overexpression of mei-P26 induces a phenotype similar to knockdown, indicating a concentration-dependent dominant negative interaction, and how wMel’s exacerbation of this phenotype suggests it contributes to these negatively-interacting elements.

“Overexpression of *mei-P26* produces tumors in both uninfected and infected ovaries, however, infected ovaries exhibit significantly more tumors than wild-type (p=1.6e-3, Fisher’s exact test; Fig 5I, table S11). **Finding that an excess of *mei-P26* recapitulates the tumorous phenotype induced by a lack of *mei-P26* indicates that this gene exerts a concentration-dependent dominant negative effect on its own**

function. Given that infection with wMel makes this dominant negative phenotype more severe, we hypothesize that wMel may make a factor that acts to enhance Mei-P26 function, potentially through a direct interaction or redundant function. “

- *L.404-405: the use of the term "wild-type" is confusing in this sentence.*

We removed the term “wild-type” from the sentence. We were attempting to indicate stocks with no known fertility mutations, i.e., with “wild-type”-like fertility. However, we understand how this was confusing since these are lab stocks with driver and balancer mutations.

- *L. 416: a color code is missing for Fig. 6B*

Thank you for pointing this out. We added a color code to the updated figure.

- *L. 557: This genotype is wrong.*

Thank you for pointing out the typo - we’ve added a semicolon and capitalized “B” to the genotype: “w[1]; Sp/Cyo; Sb/TM6B, Hu”

Reviewer #2: In this manuscript, Russell et al. demonstrate that Wolbachia infection in Drosophila melanogaster is capable of rescuing defects in germline stem cell self-renewal and differentiation. Wolbachia bacteria of arthropods are some of the world's most successful endosymbionts in terms of spread to new hosts. Although the host reproductive manipulations of Wolbachia are infamously tied to their global spread, there is doubt that these manipulations alone could fully account for their success and many have speculated or investigated other contributions of Wolbachia to host fitness. The authors show that compensation for host reproductive defects may be one such mechanism, which is an important finding for basic and applied Wolbachia research. In particular, the authors identify mei-P26 as a specific host factor where a loss of function causes various developmental defects in oocytes that are rescued by Wolbachia infection. The study is comprehensive in its analysis and presents ample evidence of an interaction between the symbiont and mei-P26. The results are important for answering key questions in the field. There are some comments and suggestions below:

Comments:

* *It would help to explain more in the main text how and why mei-P26 was chosen as a candidate. There is a section in the methods on it, but it would help at the beginning of the results or somewhere in the introduction to go into more detail on this. Since the paper focuses so much on it, it would help the reader to walk through the logic of how this candidate was identified out of all other possibilities.*

This is a great idea. We added the following statement to the last paragraph of the introduction to explain how we found *mei-P26*:

“In a previous screen, our lab identified mei-P26 as a factor that influences wMel infection intensity in D. melanogaster cell culture, potentially through its ubiquitin ligase domain [36]. Mei-P26 is a Trim-NHL protein that confers a wide range of functions through its multiple domains: its protein-binding NHL and b-box domains allow it to act as an adapter for multiple translational repressor complexes (e.g., Nanos (Nos) and Argonaute-1 (AGO1)). Its E3-ubiquitin ligase domain likely enables it to modulate proteolysis [37] (see fig S1 and S2). Given mei-P26’s in vitro role in infection [36] and its in vivo role in GSC maintenance [37], differentiation [38,39], and meiosis [39,40], we selected it as a candidate gene for wMel interaction.”

*   *How common are mei-P26 or similar GSC deficiencies in the wild? Would Wolbachia rescue of these defects be expected regularly, and would this ability commonly increase Wolbachia's fitness?*
Great question! True deficiencies should not be found at high rates in any uninfected population because they will not produce enough offspring to rise in frequency. At least 19 alleles of *mei-P26* have been sampled from nature (Flores et al. 2015) and *mei-P26* has a high Fst (measure of population differentiation)(Anderson et al. 2009). However, knowing the nature of alleles with and without *Wolbachia* requires careful experimentation, which has not yet been done. Perhaps some of these alleles are nulls that are balanced by *Wolbachia* infection? That would make for a fun future experiment.

These rescue phenotypes can increase Wolbachia fitness through the following mechanisms: if Wolbachia is able to rescue genetic or developmental errors in early embryogenesis that 1) increases the efficient use of host resources (more eggs hatch) and 2) increases the number of infected individuals (more offspring), directly increasing the Wolbachia population size.

*   *Would you expect to see mei-P26 mutants maintained in the wild with Wolbachia infection?*
Following from my answer above: yes, if *Wolbachia* can sufficiently compensate for the loss of *mei-P26* function and vertical transmission is efficient enough, then *mei-P26* knockdowns could rise to high frequencies in the wild with *Wolbachia* infection.

To address this comment and the previous comment, we've added the following (in bold) to the final paragraph of the Discussion:

"Understanding how Wolbachia interacts with host development at the molecular level is essential to deploying these bacteria in novel hosts and relying on vertical transmission to maintain them in host populations. CI has been a powerful tool for controlling host populations [76] and driving Wolbachia to high infection frequencies [3]. **The continuous presence of bacterial reproductive manipulators in host germline stem cells opens the opportunity for compensatory developmental functions to evolve, as we have shown for mei-P26. While it is not known if any of the naturally occurring mei-P26 alleles [31,75] confer loss of function that is remedied by the presence of Wolbachia, natural variation in developmental genes could have provided the selection pressure for wMel to evolve its beneficial reproductive functions.** Given that selection will favor the loss of CI if beneficial impacts on host fertility are realized [8], it is imperative that we understand the mechanisms underlying beneficial reproductive manipulations such as fertility reinforcement."

*   *Some of the main comparisons of fertility across some fly genotypes are not quite appropriate. For example, the percentages highlighted in L124-130 compare the fertility of mei-P26 lines to wild-type lines. If the question being asked is whether Wolbachia rescues a mei-P26 deficit, then the main comparison should be between a mei-P26 deficient background either with or without the symbiont. It is ok to compare to the wild-type in other cases (for example, where the question being asked is how similar the Wolbachia rescue of defects is to wild-type). However, in the experiments with fertility, it would be most appropriate to focus primarily on within-genotype comparisons.*
Thank you for your opinion and viewpoint. Indeed, the question we are asking is "*whether Wolbachia rescues a mei-P26 deficit*". However, we disagree that *" the main comparison should be between a mei-P26 deficient background either with or without the symbiont*" because fertility deficiencies are in reference to a wild-type maximum. We performed mutant, wild-type, and paired infected/uninfected crosses in parallel so that we could compare the fecundity results.

All of the within-genotype comparisons were indeed significant, and are presented Fig 1A-C. However, without rooting these mutant values to the wild-type values, it is unclear in what direction or to what degree the infection “rescued” the mutant genotype.

To further emphasize that the within-genotype comparisons were significant too, we added thorough comparisons of each to the first section of the results:
“Infection with *w*Mel *Wolbachia* significantly rescued fertility defects induced by the loss of *mei-P26* function relative to the Oregon R (OreR) wild-type strain, as measured by offspring produced per female *D. melanogaster* per day (Fig 1A-E, fig S3; p<=2.2e-16 to 2.9e-2, Wilcoxon rank sum test; see tables S2-6). Offspring production requires successful maintenance of the GSC, differentiation of a GSC daughter cell into a fully developed and fertilized egg, embryogenesis, and hatching into a first-instar larva. Breaking offspring production down into egg lay and egg hatch components (Fig 1B,C) revealed that as allelic strength increased, from *nos*-driven RNAi to hypomorphic and null alleles, fertility impacts shifted from those that impacted differentiation/development (egg hatch) to those that also impacted egg production (egg lay).

Infection with *w*Mel mediated a significant degree of fertility rescue for all allelic strengths and nos:GAL4>UAS-driven RNAi knockdowns, suggesting a robust bacterial rescue mechanism that compensates for loss of protein dosage and function. Rescue was more than complete for *nos*-driven *mei-P26* RNAi depletion (from 26% deficient offspring production in uninfected flies): *w*Mel-infected *mei-P26* RNAi females produced 44% more offspring than infected OreR wild-type females (37 versus 25 larvae/female/day, p<=1.15E-02 Wilcoxon rank sum test, table S4 and Fig 1A,E) due to a higher rate of egg production (p<=6.99E-05 Wilcoxon rank sum test, table S5 and Fig 1B,E), opposed to a higher hatch rate (Fig 1C,E and table S6). This may suggest a synergistic function between *w*Mel and low-dose *mei-P26* in the GSC. In uninfected and *w*Mel-infected *mei-P26[1]* hypomorphic females, offspring production decreased 94% and 73%, respectively, compared to OreR wild-type of the matching infection status (p<=2.26E-14 and 3.45E-12 Wilcoxon rank sum test, respectively, table S4 and Fig 1A,E). Comparing uninfected and *w*Mel-infected female *mei-P26[1]* fecundity revealed that *w*Mel infection increases the number of offspring produced per day (1.6 versus 7 larvae/female/day, p<=9.37E-05 Wilcoxon rank sum test, table S4 and Fig 1A) through increasing both the rate of egg lay (p<=2.82E-02 Wilcoxon rank sum test, table S5 and Fig 1B) and egg hatch (p<=2.55E-03 Wilcoxon rank sum test, table S6 and Fig 1C). In uninfected and *w*Mel-infected *mei-P26[1]/mei-P26[mfs1]* trans-heterozygous females, offspring production decreased 99% and 82%, respectively, relative to OreR wild-type (p<=3.40E-04 and 5.01E-10 Wilcoxon rank sum test, table S4 and Fig 1A,D). Comparing uninfected and *w*Mel-infected trans-heterozygous flies showed that infection elevates offspring production (0.2 versus 4.6 larvae/female/day, p<=1.03E-05 Fisher's exact test, table S4 and Fig 1A) through increasing the rate females lay eggs (p<=4.71E-05 Wilcoxon rank sum test; Fig 1B) and the rate those eggs hatch (p<=4.20E-03 Wilcoxon rank sum test; table S6 and Fig 1C). In females homozygous for the most severe allele, *mei-P26[mfs1]*, offspring production decreased 100% and 97% in uninfected and *w*Mel-infected flies relative to wild-type, respectively (p<=5.14E-06 Fisher's exact test, table S4 and Fig 1A). Offspring production was marginally rescued in *mei-P25[mfs1]* flies by *w*Mel infection (0 (uninfected) versus 0.76 (infected) larvae/female/day, p<=5.14E-06 Fisher's exact test, table S4, Fig 1A), in part due to infected flies laying eggs at a higher rate than uninfected flies (p<=3.04E-05 Wilcoxon rank sum test, Fig 1B). However, no female laid 20 or more eggs in one day, precluding an estimation hatch rate rescue (Fig 1C). See Supplemental Methods, tables S2-6, and fig S3 for a full description of the fecundity assays and data.”

*   *Related to this, was there a control for the RNAi knockdown line that was used throughout the paper? I did not see one in the supplemental tables. For example, were RNAi knockdown results of mei-P26 ever*

compared to knockdown of a control gene like GFP? This is an important control to determine if there is an effect of the RNAi expression system on the phenotype. The inclusion of other mei-P26 deficient fly lines mitigates the concerns of the lack of this control somewhat, however, it may be beneficial (though perhaps not strictly necessary) to conduct one of the key assays with an RNAi control if there wasn't one already.

Yes, we did run a nos>Gal4:RFP control during the same timeframe in which the other crosses were run. These data are now included in the paper (table S3-5). There is no effect of RFP expression on fecundity in regards to infection - uninfected and infected nos:Gal4>RFP fecundities are indistinguishable and are the same as OreR values, except uninfected nos:Gal4>RFP hatch rates are lower than uninfected OreR rates. Thank you for recommending that we include these data.

* *It is somewhat difficult to follow the supplemental tables without spending significant time looking at them. This is primarily because there are many features that are not explained in the captions. Though many can be inferred by spending some time studying the tables closely, it would help readers to include more detail in the captions to avoid having to do this. For example, what do the colors mean? Why were the n values so different across comparison groups? What are the unfilled boxes? What are all of the shorthand notations for genotypes? Etc. It would help the readability of the tables to include more details on the meaning of each component.*

Thank you for this feedback. Colors are used to enhance readability between lines without repeating data, but we see how these colors are confusing. Since they did not have more intrinsic meaning beyond the table, we have removed them and simplified the tables. However, we did retain the p-value color codes, and detailed their meaning in the legends. We also added full genotypes to enable easier cross-referencing with the methods section.

The n-values are large across comparison groups because of the different sampling efforts required for each genotype. All mei-P26 knockdown crosses needed to be controlled with OreR crosses performed at the same time. This resulted in more OreR crosses than any single *mei-P26* knockdown genotype.

* *It would help to include a few more details on experimental design for the fertility crosses. Since these crosses are all being compared to each other, were they all conducted at the same time using similar controls and conditions? How many separate times were the experiments conducted/repeated? This is particularly important for the CI/rescue crosses, where they need to be performed on the same day for accurate comparison and other details are important to know: were the older and younger males all the first-emerged flies from their vials, did they all have similar-aged grandmothers, etc. It would help to have more of these details in the methods.*

We performed the fecundity crosses in a highly regular and controlled manner that enabled us to 1) obtain infected OreR males from females of known age, 2) regularly have OreR virgins and males of known age to as controls for mei-P26 knockdown crosses, and 3) cope with the low levels of fertility, delayed developmental times, and reduced lifespan of uninfected *mei-P26* mutant flies. This means that most data points were collected in parallel with an OreR control and the paired infection state for the genotype. We observed no bias across our collections and have made the data fully available for reviewers and readers to access (new table S2 and fig S3). In our opinion, the fact that we observe a consistent effect across multiple genetic crosses and many fecundity cross time points strengthens the significance of our results and demonstrates its reproducibility. The following clarifying points to address your questions have been added/refined in the Supplemental Methods section:

“Overview: SLR performed the 3002 fecundity crosses continuously between October 2020 and January 2022, in batches based on their eclosion date (raw data in table S2). Infected and uninfected OreR lines were maintained continuously to 1) control the age of grandmothers of CI males, 2) regularly have OreR virgins for *mei-P26* and CI fecundity assays, 3) collect and age WT females for the aged fertility assay, and 4) obtain large sample sizes from *mei-P26* fertility mutants. The full fecundity dataset is plotted in fig S3 and contained in table 2.

**Fecundity cross category**	**Female age**	**Male age**	**Parallel control crosses**
wMel-infected mei-P26 rescue	3-7 days old	3-7 days	Uninfected and wild-type
wMel-infected OreR wild-type	3-7 days old	3-7 days	Uninfected OreR wild-type and other “wild-type” fertility stocks (e.g., nos:Gal4/CyO)
aged wMel-infected mei-P26 rescue	2-25 days old	3-7 days	Uninfected and wild-type
aged wMel-infected OreR wild-type	2-45 days old	3-7 days	Uninfected OreR wild-type
cytoplasmic incompatibility in wMel-*D.melanogaster* OreR	3-7 days old	0 or 3-7 days (from 3-7 day old mothers)	Rescue, reciprocal, and uninfected
cytoplasmic incompatibility in wRi-*D.simulans w[-]*	3-7 days old	0 or 3-7 days (from 3-7 day old mothers)	Rescue

Cross conditions: Food (vials of Bloomington’s white food recipe), laying media (grape food), and incubation conditions were made in-house in large batches monthly. Grape spoons: Approximately 1.5 mL of grape agar media (1.2x Welch’s Grape Juice Concentrate with 3% w/v agar and 0.05% w/v tegosept/methylparaben, first dissolved at 5% w/v in ethanol) was dispensed into small spoons, allowed to harden, and stored at 4°C until use. Immediately prior to use in a cross, we added ground yeast to the surface of each spoon.

Preparation of flies for fecundity crosses: Paired wMel-infected and uninfected genetic crosses were performed in parallel for the four *mei-P26* mutants (RNAi, [mfs1], [1], [mfs1/1]) to produce homozygous virgin females of both infection states for parallel fecundity crosses. Mutant genetic crosses were performed multiple (three or more) times across the 14 months to produce homozygotes from many different parents and matings. CI crosses were performed with males from infected grandmothers that were 3-7 days old. Males were aged either 0 (collected that day) or 5 days, depending on the cross. We collected males from vials over multiple days, so the majority of males were not the first-emerged.

Virgin female flies collected for fecundity crosses were transferred to fresh food and aged an average of 5 days at room temperature (from 3 to 6), or longer for the aged OreR, *mei-P26 RNAi*, and *mei-P26[1]* fecundity crosses. Males and virgin female flies were stored separately and males were aged 3-6 days. Long-term aged virgin females were kept as small groups in vials of fresh white food. Every few days the vials were inspected for mold and flies were moved on to fresh food.

Fecundity cross protocol: In the afternoon of the first day of each cross, one male and one female fly were knocked out on a CO_2 pad, added to a vial containing a grape spoon, allowed to recover, and then transferred to a 25°C constant humidity incubator on a 12 light/dark cycle to allow for courting and mating. The following

day, each spoon was replaced with a fresh spoon, and the vials were returned to 25°C for more mating. On the third day, we removed the flies from the vials, counted the number of eggs laid on the spoon's grape media, and replaced the spoon in the vial at 25°C for two days. After approximately 40 hours, we counted the number of hatched and unhatched eggs. The exact times and dates for all steps in all crosses were recorded (table S2) and plotted to show consistent results across the timeframe (fig S3).

Fecundity cross analysis: Fecundity data were parsed using perl scripts and plotted in R. Individual crosses were treated as discrete samples. Hatch rates were calculated from samples that laid 20 or more eggs (for 5-6 day old female plots) and 10 or more eggs (for age vs. hatch rate plots). Egg lay and offspring production rates were calculated from raw counts, divided by the fraction of days spent laying (metadata in table S2). Crosses with zero laid eggs (and offspring) were not rejected, as the fertility mutants often laid zero to few eggs. Our cross conditions were highly consistent and run daily, lending confidence to these zero-lay/offspring samples (figure S3). Drosophila fecundity vs female age data were fit in R using the stat_smooth loess method, formula y~x, with 0.95 level confidence interval."

* *There are some small details that would be good to include in figure captions that are missing. For example, what does each dot represent in the beeswarm boxplots? Are those hatch rates of individual females? Is this the combined data of multiple replicates?*

Thank you for this suggestion. We've added the following details (in bold) to the figure legend:
".A-C) Beeswarm boxplots of A) overall offspring production, broken into B) eggs laid and C) eggs hatched, **from single female** RNAi, hypomorphic, and null mei-P26 knockdown flies **crossed to single OreR males of the same infection state".**

* *For the fertility assays as well, a couple of minor points are unclear. It would help to explain the term "allelic strengths" (L123) the first time it is brought up, as it is unclear until later in the paragraph.*

Thank you for this helpful insight. We moved the explanation from lower in the paragraph to up before the first use of "allelic strength".

* *It may also help at the beginning of the results section to simply introduce the basic experimental setup, including which fly genotypes are being used and why, before going into the results. As is, the reader goes straight into the conclusion based on the results without being introduced to the assay that was performed.*

This is a great idea, as long as we have the space for it. We added an introductory paragraph to the results that describes the fly and Wolbachia resources and the main assays performed in the paper:

"Here, we explore the effects of wMel Wolbachia infection on the *D. melanogaster's* essential fertility gene *mei-P26* through dosage knockdown with an RNAi construct, disrupted function with a hypomorphic allele, *mei-P26[1]*, and full knockdown with a null allele, *mei-P26[mfs1]*. Using fecundity assays, immunocytochemistry, and dualRNAseq, we show that wMel can compensate for the loss of *mei-P26* to significantly rescue host fertility."

* *It is a little unclear what is meant by offspring production in Figure 1/S2/S5. Are the offspring produced not the same as the number of eggs laid or hatched?*

Offspring are the numbers of hatched larvae produced per female per day. They are directly calculated from the eggs laid per female per day and hatch rate data. We have tried to head off any misunderstanding about this with the following statements (in the original text):

From the beginning of the Results:

“Breaking offspring production down into egg lay and egg hatch components revealed that as allelic strength increased, from *nos*-driven RNAi to hypomorphic and null alleles, fertility impacts shifted from those that impacted differentiation/development (egg hatch) to those that also impacted egg production (egg lay). “

From later in the Results:
“We analyzed egg lay, egg hatch, and overall offspring production rates independently to detect wMel effects on multiple components of fecundity”

Please let us know if this is unclear and how we might clarify our meaning to the reader.

* *L155-156: It may help to add that FtsZ is a Wolbachia marker*

Thanks, we added the bolded word to the Fig1 legend:
“blue=**wMel** anti-Filamenting temperature-sensitive mutant Z (FtsZ)”

* *L179-181: It's stated here that wMel also rescues fertility impacts of mei-P26 in males, but the figure only shows a couple of significant differences, and in different genetic backgrounds across S2D-F, so it looks like the phenotype may not be as robust in males.*

Yes, you are absolutely correct, and it is well-known in the literature that *mei-P26* is less essential for male germline maintenance and differentiation. To avoid any misunderstanding, we have added the following (in bold) to the text:

“The wMel rescue mechanism is not specific to females, as the **weaker** impacts of mei-P26 loss on male fertility are mitigated by wMel-infection”

* *FigS2G,H: It would help to have a few details on these vials, maybe in the methods or in the caption. Were these vials set up on similar days with similar numbers/ages of flies? It may help to label exactly what the most important differences are between the vials, particularly for people without fly experience. Is it just the color? If so, it may help to point out the color difference in the caption so readers know what to compare between the images.*

Great suggestions! We were not exactly sure how to present a stable stock as data, so this was helpful. We added these details to the figure legend.

* *L272/Figure 3: It doesn't look like region 2a is significant between the two mei-P26 groups*

Yes, you are absolutely right. We had thought that we had described that adequately, but had not. The results section describing that figure now reads:

“ Infection with wMel increased Sxl expression in germarium region 1, **had no impact on region 2a**, and suppressed expression in region 2b relative to uninfected *mei-P26[1]*, replicating an expression pattern similar to that seen in wild-type germaria”

We also remembered to update the cartoon plot depicting protein expression across the germarium.

* *Figure 6: It would help to add details on components of the figure like color and shape meanings.*

Thank you, we added a key to convey the symbols’ meanings.

Minor comments:

There are some typos and small formatting errors in the document. For example, species names are not always italicized and the w in wMel should be italicized as well

Thank you for this suggestion. We are aware of the convention you are referring to regarding writing wMel vs *w*Mel. However, we did not think it was in modern use at high rates. For example, these papers published this year did not italicized the "w" in strain names:

Vancaester E, Blaxter M. Phylogenomic analysis of Wolbachia genomes from the Darwin Tree of Life biodiversity genomics project. Teixeira L, editor. PLoS Biol. 2023;21: e3001972. doi:10.1371/journal.pbio.3001972

Liu B, Ren Y-S, Su C-Y, Abe Y, Zhu D-H. Pangenomic analysis of Wolbachia provides insight into the evolution of host adaptation and cytoplasmic incompatibility factor genes. Front Microbiol. 2023;14: 1084839. doi:10.3389/fmicb.2023.1084839

On the other hand, these papers did:

Hussain M, Zhang G, Leitner M, Hedges LM, Asgari S. Wolbachia RNase HI contributes to virus blocking in the mosquito Aedes aegypti. iScience. 2023;26: 105836. doi:10.1016/j.isci.2022.105836

Dou W, Sun B, Miao Y, Huang D, Xiao J. Single-cell transcriptome sequencing reveals *Wolbachia* -mediated modification in early stages of *Drosophila* spermatogenesis. Proc R Soc B. 2023;290: 20221963. doi:10.1098/rspb.2022.1963

Additionally, the materials being produced by the Bordenstein Lab's Wolbachia project are advising the italicized convention:
https://www.vanderbilt.edu/wolbachiaproject/wp-content/uploads/sites/267/2021/10/Lab5_III_WolbachiaNaming.pdf

So, we changed the strain names as suggested. Thank you again for pointing this out. Many journal fonts make the italic "w" difficult to see.

"pipientis" is misspelled in the introduction (L70)

Thank you, this is corrected.

Some of the references to figures or tables are incorrect. These should be doublechecked throughout the document, for example:

- *L137: should say Fig S2E-G, not S3E-G*
- *L141: is S3 correct here?*
- *L179: missing table reference?*
- *L181: should be Table S3 not S4*

Table S1/S2 titles: should "females" be plural?

The sentences in L168-171 seem to potentially be duplicates

Thank you very much for catching these errors. We have made these corrections and gone through the manuscript very carefully to find any additional errors.

Reviewer #3: Russell et al. have investigated how Wolbachia bacteria affect Drosophila fertility. They make a major discovery in finding that Wolbachia infection substantially rescues infertility caused by the mei-P26 mutation, and provide insight relating this to previous interactions observed with other germline genes. The data largely support the authors' conclusions (excepting point 4 below) but there are substantial issues with data presentation and organization that made this manuscript challenging to evaluate.

1) Results are often not connected to the relevant figure/table, or cite an incorrect table/figure. A wide range of figures is often cited at beginning of paragraphs with specific results that follow being not cited, leaving the reader to wade through multiple figures and tables to find the data. For examples, lines 124-130 appear to match to Fig 1E, but this is not stated. Lines 140-141 appear to match to Fig 1D but is cited as S3. Line 160 'differentiation defects' appears to refer to Fig 1H-K but is not cited. Data for the results in the paragraph starting on L160 are difficult to find. L244-246 - where is data?
Thank you and the other reviewers for catching these errors. Many of these were in the paragraph you refer to below, and have been removed. With all of the new and revised supplemental material, we have gone through carefully to make sure all elements are cited in the appropriate places.

2) Data presentation is confusing. Line 160/161 is redundant with L120/121. L161-164 is redundant with L124-125. L168-171 says the same thing twice.
Thank you (and Reviewer 2) for pointing out the redundancies between these paragraphs. We were trying to highlight the elevated fertility in wMel-infected *mei-P26* RNAi females, but ended up being redundant. We've reduced these two paragraphs to one and deleted the redundancies.

There is confusion in some places whether the result being discussed is of mutant relative to wild type, or mutant with and without Wolbachia infection. For example, L166-167 appears to be making a statement about RNAi knockdown phenotype of mei-P26. But following sentence on Line 168-169 instead provides data on RNAi with and without infection.
This whole paragraph has been an issue, and we decided to remove it. We have tried to clarify exactly which genotype, infection state, and comparison is being made elsewhere in the text.

The authors sometime start a paragraph stating a result, then give background/motivation on why doing the experiment, and then give more results. Which is confusing. Such as paragraphs on p. 15.
We agree that this is confusing. Thank you for pointing these out. We have cleaned up this writing, which was the result of the authors' different stylistic opinions.

What's the difference between Fig 1G and S2A?
Nothing, these were redundant. S2A has been removed.

L378. What are the 'differences among these tumor phenotypes'?
We were referring to the morphological differences between tumors that result from nurse cell overamplification, versus stem cell loss, versus a combination of the two. This was explained earlier in the section "Mei-P26-deficient flies over-proliferate nurse cells and partially-differentiate GSCs to produce tumorous germline cysts". Also, we changed this paragraph due to Reviewer 2's comments, so further clarification is no longer warranted.

L178 - "across the fly lifespan". Does not seem to match Fig S2A-C, which shows effects reversing around day 30.
Thanks for spotting this clarification. We updated the results accordingly:

"Infection with wMel elevates the number of offspring produced per day across the fly lifespan in *mei-P26[1]* hypomorphic and *nos*-driven RNAi-knockdown females (p=1.5e-11 to 3.4e-2, Kolmogorov-Smirnov Test; Fig 1F,G; table S6). However, the underlying number of eggs laid per day and the hatch rate do vary considerably with age for both infection states and genotypes (fig S4A-D; table S6)."

L214-214. Unclear what 'young' means here.
Thank you for asking about this - we quantified GSC numbers in 4-7 day old and 10-13 day old females, which are the "young" and "aged" groups, respectively. We see that these designations are a bit confusing in this paragraph, so we removed them. However, they are retained in the next paragraph, which discusses the aged vs young data. So, we introduce the terms earlier in the section.

L434. 'differences … are insignificant'. In Table S4, some results are significant and some are not.
This was confusingly written. We have rephrased these results to hopefully be clearer about what was significant and what was not about the OreR lifespan fecundity plots:

"We measured OreR fertility across its lifetime (~50 days), and found that wMel may produce higher lifetime fecundity relative to uninfected flies. While maintaining uninfected levels of egg production (Fig 6B, table S6), wMel maintains elevated OreR egg hatch rates as flies age (p=7.1e-9 Kolmogorov-Smirnov Test; Fig 6C; table S6), culminating in age ranges in which infected flies appear to produce an excess of offspring relative to uninfected (~20-30 days old; Fig 6A)."

LL547. FS2 should be FS1?
We do not see the indicated item "FS2" on line 547

Fig 1. Should emphasize that all data is from days 2-3 only (I think).
Thank you for pointing this out. We added this info to the figure legend to make sure it is clear.

Fig6A - what is parent age?
Three to seven days for both males and females, like the other standard (not aged) crosses. This plot is now a part of Fig 1C, but this information has been clarified throughout.

Fig 6B,C - presumably is also OR-R?
Yes, thank you. This has been clarified.

3) Comparisons of mutants vs wild type.
At many places mutant genotypes are compared for fertility phenotypes to wild type. One potential issue is experimental design. Presumably with and without Wolbachia comparisons within a genotype were done in parallel (at same time). Is that also true for different genotypes (e.g. OR-R and mei-P26 mutants)? If not, then that weakens the comparative power among genotypes. A second issue is the different genetic backgrounds. Different wild type backgrounds can have wide variation in fertility, and that will also occur here since mutants and wild type are different backgrounds. It therefore is unclear what the biological meaning is that some mutant conditions have higher fertility than wild-type (eg. L163-164). For these reasons, I would suggest de-emphasizing comparisons of fertility between mutants and wild-type, and focusing on paired comparisons of fertility phenotypes with and without Wolbachia within individual genotypes.

Thank you for expressing these concerns. Indeed, fecundity assays have lots of potential for error and bias. However, we took much care to design our workflow to minimize bias, increase reproducibility, and continuously run controls, while completing more than 3000 single female-by-male crosses. We have added many extra details to our methods section, as described in response to Reviewer 2's comments above. We also added plots of the fecundity collections by time, so you can see that the results were consistent and controlled across the timeframe, and included the full fecundity dataset as a supplemental table.

To address your specific concerns:
Yes, we performed OreR and mutant crosses in parallel, alongside paired infected and uninfected crosses. Given that we used OreR males in all of our mutant female crosses and the mutant-vs-OreR fecundity data are paired, it seems reasonable to include them as a comparison point for estimating rescue values.

We understand the concern with different, uncontrolled backgrounds. That is why we reproduced these experiments with three different *mei-P26* knockdowns (RNAi, the mei-P26[1] hypomorphic allele, and the null *mei-P26[mfs1]* allele. Finding elevated fertility in the infected version of all of these mutants indicates that *mei-P26* is indeed being rescued. While we cannot say for sure that some of the variance among rescue proportions is not also due to some other underlying variation, our conclusions are still supported.

4) Wolbachia effects in wild type.
I wouldn't call Gal4-expressing lines "wild type". At some places the authors seem to agree (lines 403-405), but at other places including in the Highlights and Abstract, they conflate the genotypes. This is important to clear up because a major claim of the paper is that Wolbachia increase fitness even in wild type individuals. If I understand all the data on this point, in true wild type (OR-R), only egg hatch rate is significantly increased by Wolbachia infection (S5C). But egg lay is somewhat reduced (S5B). Thus, total offspring is not different (S5A), which is the phenotype that ultimately matters for fitness. So the general conclusion that fitness is increased by infection in wild type lines does not seem to be established.
Wild-type effects are a very important point that we do not want to over state - thank you for these comments. We completely agree that we have not shown a wild type "fitness" increase because we only show significant increases in hatch rate, not offspring production. We postulate that wMel is increasing developmental resilience, which could increase fitness over the fly lifetime. However, we see that we have been a little heavy handed with the fitness references. Mei-P26 mutant fitness is certainly increased by wMel infection, but OreR wild-type is not so clear-cut. The idea with the wild-type evidence is to provide a link between wMel's rescue of mutant fertility and how those capabilities may have been selected for in nature, in wild-type flies.

We have clarified this though the text in the following ways:

Discussion: "Here, we demonstrate that wMel infection can **reinforce host fertility** in both OreR wild-type and *mei-P26* mutants.." (replaced "elevate host fitness")

Introduction: "We report that *D. melanogaster* flies infected with the wMel strain of *Wolbachia* are resilient to mutations in the essential fertility gene, *meiotic-P26,* and exhibit **reinforced** fertility" (replaced "enhanced")

Abstract: "In OreR individuals with wild-type fertility, wMel infection elevates lifetime egg hatch rates."

Title: *Wolbachia* endosymbionts manipulate GSC self-renewal and differentiation to **reinforce** host fertility (replaced "enhanced")

Minor points.
L395. Not sure 'recapitulates' is correct word here.
You might be right… we changed it to “rescues”.

L462-3. Better to say 'rescue the PARTIAL loss'. The description of Sxl and Bam with the 'respectively' is confusing.
Good point. We changed the text.

L585-590. How were long-term experiments with collections over 60 days done?
Flies were collected immediately after eclosion and aged as virgins on fresh food that was replaced every few days. These details and more have been added to the Supplemental Methods.
