# Supplementary material for: Wolbachia endosymbionts manipulate the self-renewal and differentiation of germline stem cells to reinforce fertility of their fruit fly host
Source: PLoS Biol. 2023 Oct 24;21(10):e3002335. doi: 10.1371/journal.pbio.3002335 (PMC10597519; doi:10.1371/journal.pbio.3002335)
Supplement: Attachment — Submitted filename: R2R2_meiP26rescue_PLoSBio.pdf [file pbio.3002335.s037.pdf]

Dear Lucas,

Thank you and the reviewers for the prompt turn-around and your encouraging comments. We have addressed all of the remaining issues and released the underlying datasets to the public.

We scanned the current records for all of our cited references, and none have been retracted as of August 29, 2023. So, we did not make any changes to the reference list.

We appreciate the suggestion to deposit our protocols in protocols.io, and we have begun the process.

All of the required documents and full resolution figures are attached to our submission. Please let us know if you need anything else.

Best,
Shelbi

--
Shelbi Russell, PhD

Assistant Professor
Department of Biomolecular Engineering
University of California Santa Cruz
Sinsheimer Laboratories Rm. 427
1156 High St. Santa Cruz, CA 95064

##

Dear Dr Russell,

Thank you for your patience while we considered your revised manuscript "Wolbachia endosymbionts manipulate GSC self-renewal and differentiation to reinforce host fertility" for publication as a Research Article at PLOS Biology. This revised version of your manuscript has been evaluated by the PLOS Biology editors, the Academic Editor and the original reviewers.

The reviewers are largely satisfied by the revision and have all suggested that we accept your study. However I note they raise a number of minor suggestions that we think should be addressed before publication. Additionally, before we can accept your study, we need you to address a number of editorial requests detailed below. We therefore would like to invite one last revision that we do not think will take very long.

**EDITORIAL REQUESTS:

1) TITLE: We suggest that the title be edited to spell out GSC and specify the host, as this will make the study more broadly accessible. If you agree, we suggest you change the title to something like:

"Wolbachia endosymbionts manipulate the self-renewal and differentiation of germline stem cells to reinforce fertility of their fruit fly host"

This title sounds good to us. Thank you for the suggestion.

2) ABSTRACT: Similarly, GSC should be defined in the abstract

Agreed. It is updated.

3) FINANCIAL DISCLOSURES: Please update the financial disclosures statement, in our editorial manager system, to describe the role of any sponsors or funders in the study design, data collection and analysis, decision to publish, or preparation of the manuscript. If the funders had no role in any of the above, include this sentence at the end of your statement: "The funders had no role in study design, data collection and analysis, decision to publish, or preparation of the manuscript."

We updated our disclosure statement with the suggested text to indicate that the funders had no role in the scientific elements of the paper.

4) DATA: Thank you for providing data on NCBI and Dryad related to your manuscript. For some reason, I could not access these datasets to verify that they meet our requirements. Can you provide me with a reviewer code? (sorry if I missed this somewhere)

>>Please do take a look at our Data Policy, which requires that all data be made available without restriction, and make sure that your data provided on these repositories meets our requirements. Note that we do not require all raw data. Rather, we ask that all individual quantitative observations that underlie the data summarized in the figures and results of your paper be made available either as a deposition on a repository or as a supplementary excel file.

>>Please also ensure that figure legends in your manuscript include information on where the underlying data can be found. For example, to each figure legend you can add the sentence "the data underlying this figure can be found at ____"

We have made the data on Dryad and NCBI fully available (as of August 22nd). Our apologies for not making it easier to access prior to now. At the time of this resubmission, the Dryad data is still in review. Please use this link to access it, if it is still not public by the time you want to look at it: https://datadryad.org/stash/share/_mXMysm3z2eEn1PUosGXT0pydK54dJ2WCy5mLqOklEE.

We have also added information to each figure legend about where to find the source data. Thank you for this suggestion.

As you address these items, please take this last chance to review your reference list to ensure that it is complete and correct. If you have cited papers that have been retracted, please include the rationale for doing so in the manuscript text, or remove these references and replace them with relevant current references. Any changes to the reference list should be mentioned in the cover letter that accompanies your revised manuscript.

We expect to receive your revised manuscript within two weeks.

To submit your revision, please go to https://www.editorialmanager.com/pbiology/ and log in as an Author. Click the link labelled 'Submissions Needing Revision' to find your submission record. Your revised submission must include the following:

- a cover letter that should detail your responses to any editorial requests, if applicable, and whether changes have been made to the reference list

- a Response to Reviewers file that provides a detailed response to the reviewers' comments (if applicable)
- a track-changes file indicating any changes that you have made to the manuscript.

NOTE: If Supporting Information files are included with your article, note that these are not copyedited and will be published as they are submitted. Please ensure that these files are legible and of high quality (at least 300 dpi) in an easily accessible file format. For this reason, please be aware that any references listed in an SI file will not be indexed. For more information, see our Supporting Information guidelines:
https://journals.plos.org/plosbiology/s/supporting-information

Published Peer Review History
Please note that you may have the opportunity to make the peer review history publicly available. The record will include editor decision letters (with reviews) and your responses to reviewer comments. If eligible, we will contact you to opt in or out. Please see here for more details:
https://blogs.plos.org/plos/2019/05/plos-journals-now-open-for-published-peer-review/

Press
Should you, your institution's press office or the journal office choose to press release your paper, please ensure you have opted out of Early Article Posting on the submission form. We ask that you notify us as soon as possible if you or your institution is planning to press release the article.

Protocols deposition
To enhance the reproducibility of your results, we recommend that if applicable you deposit your laboratory protocols in protocols.io, where a protocol can be assigned its own identifier (DOI) such that it can be cited independently in the future. Additionally, PLOS ONE offers an option for publishing peer-reviewed Lab Protocol articles, which describe protocols hosted on protocols.io. Read more information on sharing protocols at
https://plos.org/protocols?utm_medium=editorial-email&utm_source=authorletters&utm_campaign=protocols

Please do not hesitate to contact me should you have any questions.

Sincerely,

Luke

Lucas Smith, Ph.D.
Senior Editor,
lsmith@plos.org,
PLOS Biology

Reviewer remarks:

Reviewer #1: My main requests have been addressed. As for my minor comment on "other germ cells", I meant nurse cells and the oocyte.

Thank you and thank you for clarifying your comment.

Reviewer #2: The authors have thoroughly addressed comments by the reviewers. In particular, they have made many clarifying statements regarding the methods, addressed concerns with figure/table citations in the text, and addressed concerns with the fertility assays. The new details on the methods for the fertility assays are helpful. The authors have also added comparisons of fertility within genotype which, paired with the comparisons of fertility to controls (and their justification for this is good), establish their points well. This addresses the questions raised by reviewers. I have only a couple of very minor suggestions remaining:

I suggest a few very minor clarifying details on the caption of Figure S4H,I (the stock images): 1) I would label the dates that each individual vial received adults (presumably older on the left to newer on the right of each image?). This would help readers know which vials should be compared to which other vials between images. 2) I would also suggest pointing out the mold growth is visible by different food colors/consistencies between comparably-aged vials in H and I. 3) I would clarify that the sentence starting with "Infection enables stable robust stock persistence..." is referring to the Wolbachia infection as opposed to the mold.

These are great suggestions. 1) We added the dates the vials were started to the figure, as well as the date the images were taken. 2) We added the following to the figure legend (bold): “Mold growth **(green food vs tan/brown food)** is uninhibited in the uninfected stocks due to embryo and larval death, which both feeds and fails to stop mold.” 3) We changed the next sentence to say (bold): “Infection enables stable robust stock persistence **because larval production outruns mold growth**.”

Also, very minor: if the authors have agreed to go with the convention of italicizing the w in wMel, then this should be also applied to supplemental documents (supplemental figure captions etc.)

Thank you for catching this. We’ve updated the uses of *w*Mel in the supplemental.

Reviewer #3: The authors have clarified many of the concerns and queries about the fertility assays. They have also added extensive discussion of new RNA-seq experiments. What does "dual-RNAseq" mean - examining both Dmel and wMel genes, or combining Or-R and mei-P26 in a single analysis?

Dual RNAseq means that we sequenced the RNA from both host and symbiont at the same time. This required that we use a less standard approach. Poly-T-tailed primers will not work on non-A-tailed bacterial transcripts. So, we needed to deplete the rRNA and then use random hexamer primers to amplify both bacterial and eukaryotic cDNA simultaneously.

We have added clarifying phrases to help the reader interpret these data and analyses (bold):
“Using fly fecundity assays, immunocytochemistry, and dualRNAseq **of both host and bacterial transcripts**, we show that *w*Mel can compensate for the loss of *mei-P26* to significantly rescue host fertility.”

“Exploring these phenotypes through dual-RNAseq **quantification of eukaryotic and bacterial transcripts** revealed that wMel infection rescues and offsets many gene expression changes induced by mei-P26 loss at the mRNA level.”

I didn't see the following in Fig 1, referring to the time frame of the eggs/offspring being analyzed.
Fig 1. Should emphasize that all data is from days 2-3 only (I think).
Thank you for pointing this out. We added this info to the figure legend to make sure it is clear.

Thank you for making sure this is addressed. We did mention these details in the second sentence of Fig 1: “female flies crossed to single OreR males of the same infection state and age (3-7 days old)”.

In case you mean for us to include the ages of the flies sampled for the cytological data, which were aged the same as the fecundity data, we've amended following the statement:
"Confocal mean projections of *D. melanogaster* ovarioles and germaria (3-7 days old)."
